# Improving Triplet–Triplet Annihilation Upconversion Output by a Triplet Mediator Approach: Mechanistic Insights on Homo and Hetero-Annihilation in Three-Component Systems

**DOI:** 10.1021/jacs.5c09906

**Published:** 2025-10-24

**Authors:** Sunil Kumar Kandappa, Victor Gray

**Affiliations:** Department of Chemistry, Ångström Laboratory, Uppsala University, Box 532, SE-751 20 Uppsala, Sweden

## Abstract

Triplet–triplet annihilation photon upconversion (TTA-UC) is a promising strategy for converting low-energy photons into higher-energy emission, with potential applications in solar energy harvesting, bioimaging, and photocatalysis. A challenge in TTA-UC systems is minimizing the reabsorption of upconverted photons by the annihilator molecules. To address this, we present a mediator-assisted TTA-UC approach utilizing a neutral mediator molecule to facilitate upconversion in the ultraviolet (UV) and visible regions. Our study introduces a general protocol, and through detailed kinetic modeling, we elucidate the underlying mechanism, highlighting the role of hetero-TTA (triplet–triplet annihilation between the mediator and annihilator). Notably, we report the first estimation of a hetero-TTA rate constant, which exceeds the homo-TTA rate by a factor of 2. This work broadens the design space for TTA-UC systems by enabling the use of neutral, noncovalently linked mediators, expanding beyond the conventional reliance on charged or covalently tethered species.

## Graphical abstract

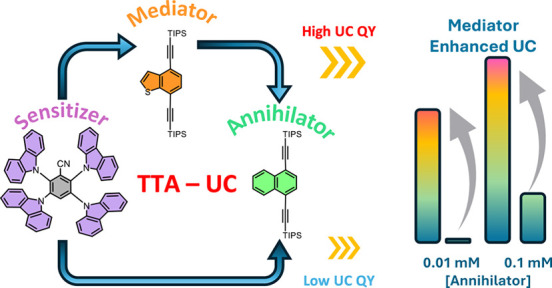

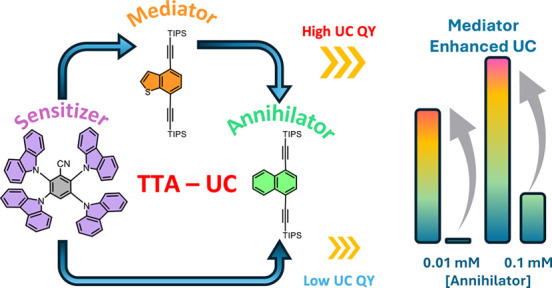

## Introduction

Triplet–triplet annihilation photon upconversion (TTA-UC), a process where the energy of two low-energy photons is combined into a photon of high energy, has gained significant attention in recent decades in the context of solar energy harvesting^1–4^ and water splitting.^5–11^ TTA-UC offers a way to harvest photons with energy lower than the band gap energy, which in a single-junction solar cell otherwise would be unutilized. Likewise, high-energy photons could be beneficial for photosensitization in water splitting, which often relies on materials with high band gap energies. Several other applications^12^ include bioimaging,^13^ photodynamic therapy,^14^ photoredox catalysis,^15^ photocatalytic degradation of volatile organic compounds,^16^ in designing soft actuators to induce photomechanical effects,^17^ OLEDs,^18,19^ 3D printing,^20,21^ and in energy storage devices.^22^

A typical TTA-UC system consists of a triplet sensitizer molecule that acts as an energy donor and an annihilator molecule that acts as an energy acceptor. The sensitizer molecule absorbs the incoming low-energy photons and eventually populates its triplet state after intersystem crossing (ISC). Triplet formation is followed by triplet energy transfer (TET) to an annihilator molecule. The triplet excited annihilator can combine its triplet energy with that of an adjacent triplet excited annihilator molecule through triplet–triplet annihilation (TTA). TTA populates a higher energy state, which ideally relaxes to the first singlet excited state (S_1_), that eventually emits upconverted light. The efficiency of the intermolecular TTA process in solutions is curtailed by the diffusion limit of annihilator and sensitizer molecules. However, with the long triplet lifetime and achievable mM concentrations of organic molecules in deaerated organic solvents, TET and TTA can proceed efficiently. Intramolecular TTA-UC is possible when two or more annihilator molecules are linked together, and it has gained interest as a means to overcome the diffusion limit.^23–27^ However, this approach is associated with the challenge of proper molecular design to achieve efficient TTA, triplet migration, and sensitization.^28^

To ensure an efficient TTA-UC process in solution, a long triplet lifetime of the sensitizer and annihilator is essential. The triplet energy transfer efficiency (η_TET_) is given by

$$\eta_{\mathrm{TET}}=1-\left[\frac{1}{1+\tau_{0}k_{Q}[Q]}\right]$$ 1

where τ_0_ is the triplet lifetime of the unquenched sensitizer, and *k*_Q_ is the bimolecular quenching rate constant with a quencher of concentration [*Q*]. To maximize η_TET_, a typical TTA-UC system usually uses a high concentration of annihilator. However, the high concentration of annihilator molecules subsequently causes an intrinsic challenge, where upconverted light will get reabsorbed by the annihilator molecule itself. Reabsorption becomes a major issue, especially when the annihilator molecule has a small Stokes shift, which is often the case in the rigid polyaromatic hydrocarbons often used as annihilators. As an example, most reported TTA-UC yields are reported after reabsorption correction, and for two traditional UC systems PdOEP/DPA (PdOEP = palladium­(II) octaethylporphyrin, DPA = 9,10-diphenylanthracene) and 4CzBN/Nap (4CzBN = 2,3,5,6-tetra­(9H-carbazol-9-yl)­benzonitrile, Nap = 1,4-bis­[2-[tris­(1-methylethyl)­silyl]­ethynyl]­naphthalene), the difference between reabsorption corrected and noncorrected UC quantum yields can in our experience approach a factor of 2. To avoid reabsorption, lowering the concentration of annihilator molecules as such is also not a viable solution since this would decrease η_TET_ (eq 1) and subsequent TTA. A strategy to maintain a high rate of TET and TTA processes with a low concentration of annihilator would minimize the reabsorption of upconverted light.

In this regard, recent work has used mediator-assisted TTA-UC to bypass some of the obstacles associated with the prototypical TTA-UC involving sensitizers and a high concentration of annihilator molecules.^29–36^ The mediator-assisted TTA-UC system consists of a third component along with the sensitizer and annihilator, which acts as a mediator for energy transfer between the sensitizer and annihilator (Figure 1). An efficient mediator needs to have a long triplet lifetime, and its triplet energy should lie in between that of the sensitizer and annihilator. Figure 1 depicts the Jablonski diagram for mediator-assisted TTA-UC.

**Figure 1:**
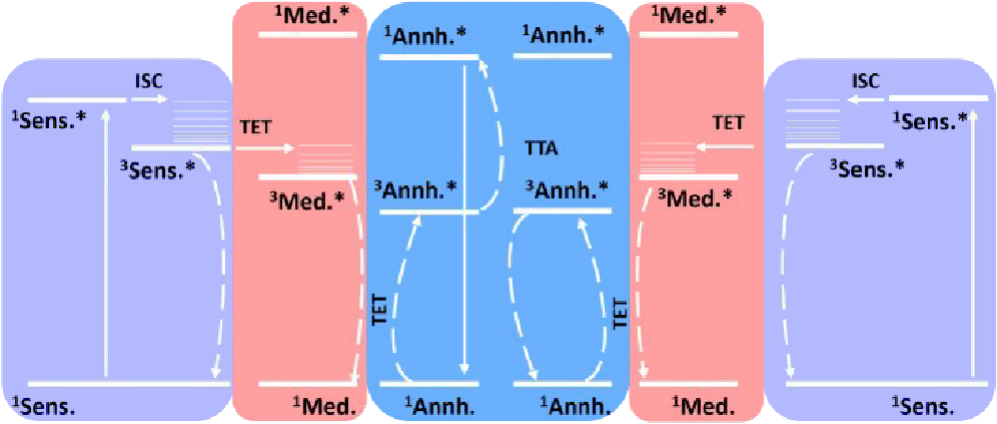
Jablonski diagram for mediator-assisted triplet–triplet annihilation upconversion (TTA-UC). Sens., Med., and Annh. refer to sensitizer, mediator, and annihilator, respectively.

A few different approaches to using mediators have been reported. Most commonly, a mediator is tethered to a sensitizer or quantum dot, effectively extending the apparent triplet lifetime of the sensitizer by ensuring fast TET from the sensitizer to the mediator.^29–35^ A more recent report from Kerzig and co-workers employed Coulombic interactions^36^ to increase the rate constant for TET between the sensitizer and mediator beyond the diffusion limit without covalent linkage. They emphasized the significance of the Coulombic interaction between the mediator and annihilator for enhanced TET. A lower efficiency of TTA-UC was observed for the system without Coulombic interaction compared to that with Coulombic interaction between the sensitizer and mediator. They also noted the positive effect of lowering the annihilator concentration for minimizing reabsorption. However, both covalent and Coulombic approaches entail certain limitations. For example, linking a mediator molecule to a sensitizer might involve a tedious synthetic approach. In those cases where sensitizer and mediator molecules are bound by Coulombic interactions, they are limited to only charged species.

A noncovalent mediator approach without Coulombic interactions was highlighted by Schmidt and co-workers^37^ in 2016 to minimize the reabsorption from annihilator molecules by reducing its concentration while maintaining high concentration of mediator molecules for an efficient TET transfer process. In their three-component system, they used a 9,10-bis­(phenylethynyl)­anthracene (BPEA) molecule as the mediator for the triplet energy transfer from the sensitizer PQ_4_PdNA to annihilator molecule rubrene for the upconversion in the visible region. Schmidt and co-workers^38^ also reported a singlet oxygen-mediated TTA-UC process with an uncharged annihilator. However, upconversion in the blue region mediated by singlet oxygen is highly unlikely, as it requires annihilator molecules with triplet energies lower than the singlet oxygen energy (0.98 eV). Other three-component TTA-UC systems include those by Balushev and co-workers,^39^ Zhang and co-workers,^40^ and also recently by Wang and co-workers.^41^ However, in these latter cases, the same or similar concentration was used for two different emitter molecules. Hence, triplet mediation is likely not the main reason for enhanced UC, rather efficient hetero-TTA would play a significant role. Furthermore, the impact of reabsorption of upconverted emission is lacking in these reports. Schmidt and Castellano discussed the kinetics of three-component systems in low triplet concentration regimes and emphasized the role of hetero-TTA, i.e., TTA between one mediator and one annihilator, when the triplet concentrations of the mediator and annihilator are equal.^42^

In the solid state, there are also reports of three-component systems consisting of a sensitizer, an annihilator, and an emitter molecule which act as a singlet acceptor (often referred to as a singlet sink). Singlet energy transfers from the annihilator to the emitter molecule have successfully been employed to mitigate singlet fission from annihilator singlets,^43–45^ but this approach differs from the triplet mediator approach, as highlighted recently by Carrod et al.^46^ In the solid-state films studied by Carrod et al., tetracene was used as the triplet mediator and rubrene as the annihilator. Monte Carlo simulations explained why, at higher intensities, the ratio between hetero-TTA and homo-TTA of the mediator shifted to favor mediator homo-TTA, which was detrimental to UC output. The reason for the majority homo-TTA in the mediator at higher intensities came from the high local concentration of mediator triplets arising from the high intensity excitation and poor triplet migration.

Such limitations have not yet been discussed in solution-based three-component systems, and to the best of our knowledge, a full mechanistic discussion of the mediator approach in solution has not yet been presented. Furthermore, mediator-assisted TTA-UC for UV-emitting annihilators is still lacking.

Herein, we show a protocol to use a neutral mediator molecule for TTA-UC in the UV region with detailed kinetic modeling, revealing the mechanism involved in the process. In our system, benzothiophene-based molecule BT (Figure 2a) was used as a mediator with a well-established UV-emitting annihilator molecule, Nap (TIPS-Naphthalene), and a sensitizer, 4CzBN.^47,48^ BT was found to be a perfect choice to act as a mediator molecule with its triplet energy (T_1_ = 2.37 eV, SI, Figure S34) lying in between that of the sensitizer 4CzBN (T_1_ = 2.71 eV)^48^ and the annihilator Nap (T_1_ = 2.12 eV).^5,48^ Additionally, the singlet energy of BT (3.54 eV) is higher than that of Nap (3.40 eV, determined from the absorption onset; Figure 2). Hence, the possibility for the former molecule to act as a singlet sink is minimized. Instead, it can efficiently act as the mediator for triplet energy transfer from sensitizer to annihilator molecules. We were able to achieve an over 10-fold increase in the triplet–triplet annihilation upconversion quantum yield (TTA-UC QY) for BT mediator-assisted upconversion in the presence of the 4CzBN sensitizer and Nap annihilator compared to the system without a mediator while maintaining the same concentration of sensitizer and annihilator (vide infra). We were able to reduce the concentration of annihilator almost an order magnitude lower than the typical concentration used for TTA-UC studies using the Nap annihilator,^47,48^ while maintaining the same order of upconversion QY. Our approach could expand the range of annihilator and mediator molecules for the TTA-UC process by not necessarily binding them together by means of covalent linkage or restricting them to be a charged species for Coulombic interactions.

**Figure 2:**
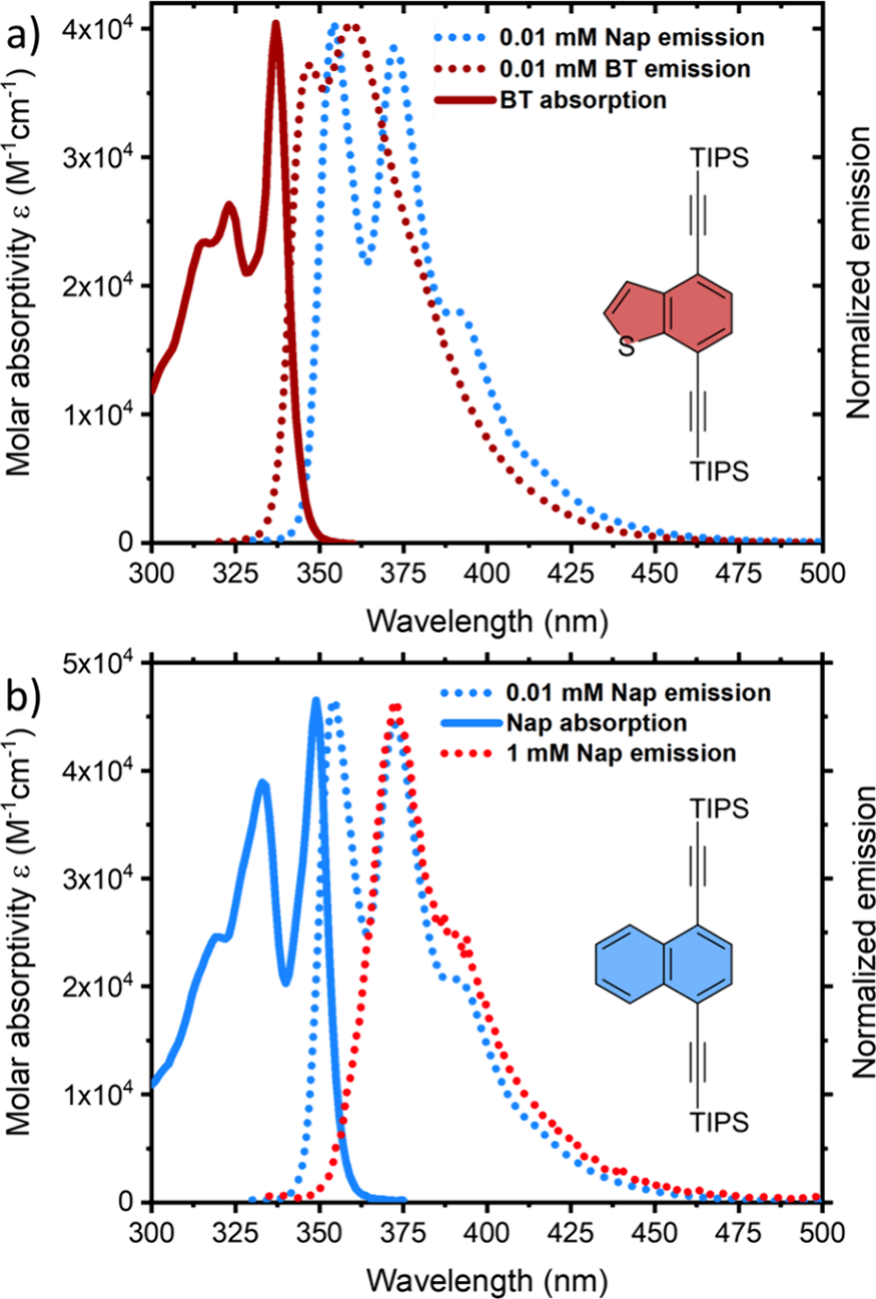
(a) Normalized emission spectra of 0.01 mM Nap (dotted blue line), 0.01 mM BT (dotted dark red line), and UV–vis absorption spectra of BT (solid dark red line). (b) Normalized emission spectra of 1 mM Nap (dotted red line), 0.01 mM Nap (dotted blue line), and UV–vis absorption spectra of Nap (solid blue line).

## Results and Discussion

### Synthesis

The mediator molecule BT was synthesized as per the previous report (Scheme 1).^49,50^ 1,4-Dibromo-2-fluorobenzene was reacted with LDA in THF at −78 °C for 45 min. This was followed by the addition of dimethylformamide (DMF) at the same temperature, and stirring for 5 min resulted in the formation of corresponding aldehyde 3,6-dibromo-2-fluorobenzaldehyde **2** in 96% crude yield.^50^ It was taken as such for the next step without further purification and treated with sodium-2-methyl-2-propanethiolate in DMF at −45 °C for 7 h to form 3,6-dibromo-2-[(1,1-dimethylethyl)­thio]­benzaldehyde **3** in 80% isolated yield. This was followed by the reaction with dimethyl­(1-diazo-2-oxopropyl)­phosphonate in the presence of K_2_CO_3_, in methanol at 0 °C for 2 h, resulting in the formation of the corresponding alkyne derivative 1,4-dibromo-2-[(1,1-dimethylethyl)­thio]-3-ethynyl-benzene **4** in 64% isolated yield. This was further treated with AuCl in a dioxane/water mixture (5:1) at rt for 10 min to form 4,7-dibromobenzo­[b]­thiophene **5** in 84% isolated yield. Sonogashira coupling of this product with (triisopropylsilyl)­acetylene resulted in 4,7-bis­[2-[tris­(1-methylethyl)­silyl]­ethynyl]-benzo­[b]­thiophene **6** in 95% isolated yield. We abbreviated this compound as BT (see SI for the detailed procedure).

**Scheme 1:**
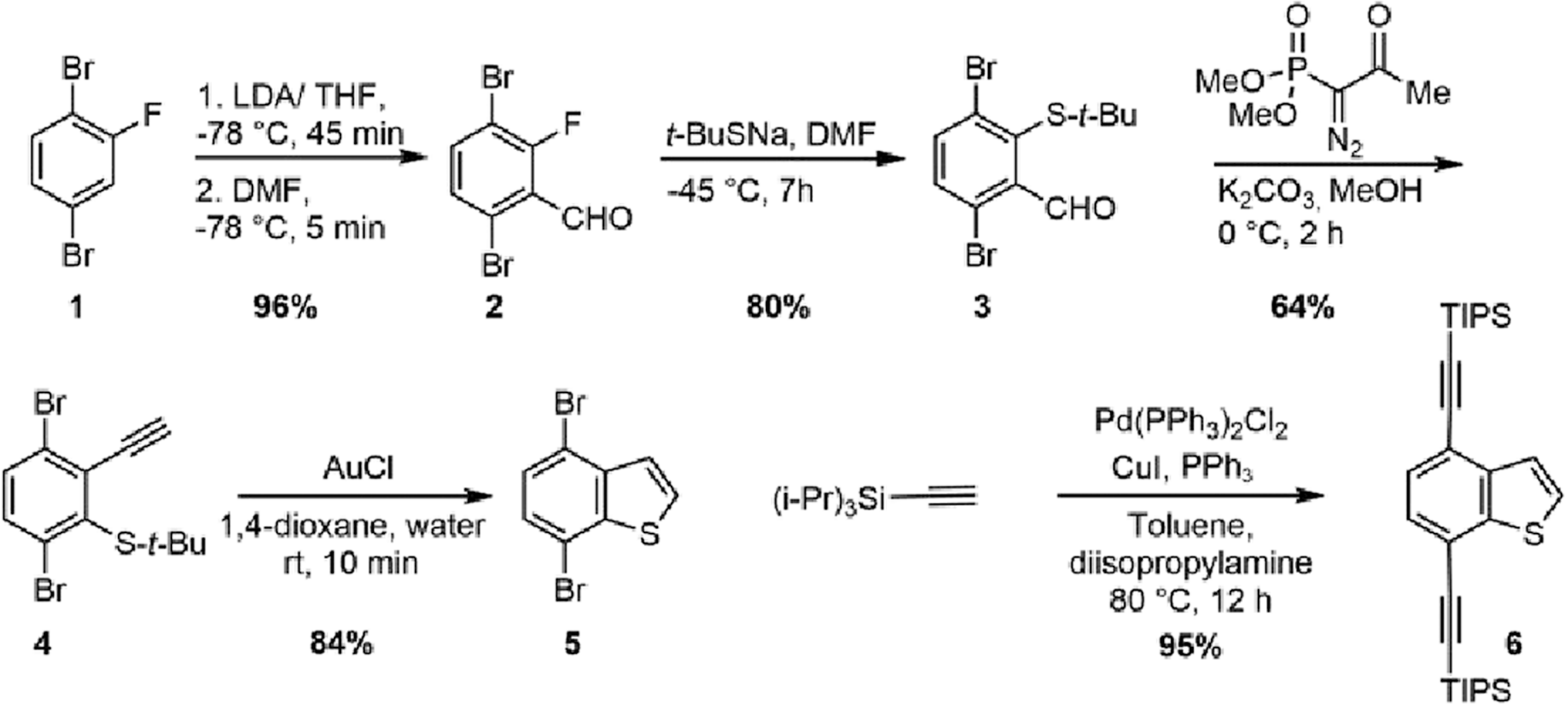
Synthesis of BT

### Photophysical Characterization

Figure 2a comprises normalized emission spectra of Nap and BT and UV–vis absorption spectra of BT in toluene. The absorption onset of Nap (0.01 mM) is around 365 nm (Figure 2b) compared to that of BT, which is around 350 nm (Figure 2a). Importantly, there is minimal spectral overlap between the emission of annihilator Nap and the absorption of mediator BT (346–350 nm, Figure 2a). Hence, BT can be used as a mediator at high concentrations with minimal reabsorption of upconverted emission from Nap (vide infra). On the other hand, Nap exhibits significant reabsorption of emitted light at higher concentrations, as evident from the decreased intensity of emission peak in the blue region (∼354 nm) on increasing its concentration from 0.01 mM (Figure 2b, blue dotted line) to 1 mM (Figure 2b, red dotted line).

### Mediator-Enhanced Upconversion

The mediator-assisted TTA-UC process involves two triplet energy transfer (TET) processes. In the first TET from sensitizer to mediator (TET^S→M^), mediator BT acts as a quencher with respect to the triplet energy of sensitizer 4CzBN. In the second TET process from mediator to annihilator (TET^M→A^), the annihilator Nap acts as a quencher with respect to the triplet energy of mediator. Higher concentration of mediator increases the efficiency of TET^S→M^(eq 1), and since BT absorption exhibits minor overlap with the emission spectra of annihilator Nap, it is possible to use BT in high concentration with minimal reabsorption of upconverted light by the mediator. On the other hand, higher η_TET_ for TET^M→A^ is accomplished by choosing a mediator with relatively high triplet lifetime. In fact, to benefit from a mediator, the mediator triplet lifetime needs to exceed the triplet lifetime of the sensitizer. The concentration of Nap was maintained at a minimal level to reduce the intrinsic reabsorption by the annihilator molecule.

For the detailed analysis of BT mediator-assisted TTA-UC studies, we vary the Nap annihilator concentration from 0.1 to 0.01 mM while maintaining the concentration of sensitizer at 25 μM (corresponding to an absorbance at the excitation wavelength 405 nm ∼ 0.17) and mediator at 1 mM. The TTA-UC QY from the three-component system is compared to the corresponding two-component system (annihilator and sensitizer only). All solutions for TTA-UC studies were prepared in vacuum-degassed toluene (5-cycles). Lowering the concentration of Nap from 1 mM to 0.1 mM in the upconversion mixture results in the UV–vis absorption onset around 373 nm to blue shift by about 10 nm (Figure S14, SI). Similarly, fluorescence of Nap blue shifts (Figure S33, SI) due to minimized reabsorption in the upconversion mixture with 0.1 mM Nap compared to that in the case with 1 mM Nap. Figure 3 summarizes the comparison of TTA-UC QYs without reabsorption correction (practical output flux of upconverted light).

**Figure 3:**
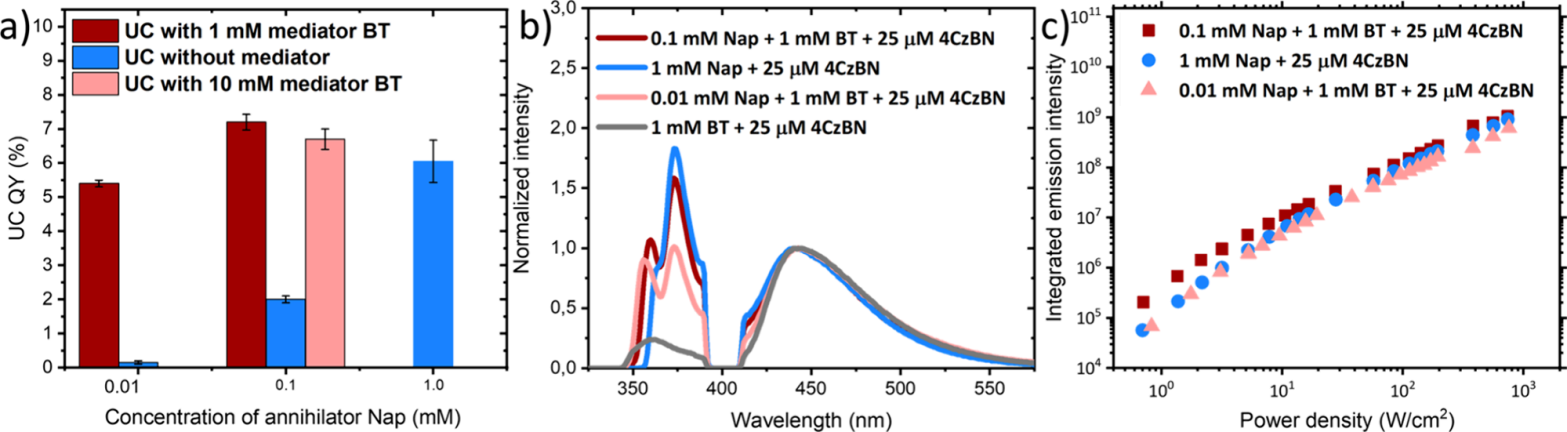
(a) Comparison of TTA-UC quantum yield for systems with sensitizer 4CzBN (25 μM) and annihilator Nap at three different concentrations (0.01, 0.1, and 1 mM) in the presence and absence of mediator. Error bar for 1 mM Nap and 0.1 mM Nap in the presence of 1 mM BT is the standard deviation measured over four independent measurements. For the rest, error bars indicate spread over two independent measurements. (b) TTA-UC emission spectra for bi- and tricomponent systems, excited at 405 nm with a power density of 830 W/cm^2^. Spectra are normalized to the sensitizer emission intensity for ease of comparison. (c) Plot of the upconverted emission intensity as a function of excitation power density.

These quantum yield data are considered for further discussion below. For comparison, TTA-UC QYs with reabsorption correction are included in Table 1. TTA-UC with a 25 μM 4CzBN, 1 mM BT, and 0.1 mM Nap mixture shows a TTA-UC QY of 7.2 ± 0.3% (Figure 3 and Table 1, entry 2), which is almost 4 times higher compared to the system without mediator (i.e., 25 μM 4CzBN and 0.1 mM Nap (Table 1, entry 3). It is noteworthy to mention that TTA-UC QY with 25 μM 4CzBN, 1 mM BT, and 0.1 mM Nap is slightly higher (7.2 ± 0.3%) than that of the traditional two-component system with higher concentration of annihilator (6.05 ± 0.5%), i.e., for 1 mM Nap and 25 μM 4CzBN (Figure 3a and Table 1, entry 1). Lowering the concentration of annihilator further to only 0.01 mM Nap with 1 mM BT mediator, the TTA-UC QY decreases slightly to 5.4 ± 0.1% (Figure 3a and Table 1, entry 4). However, in the absence of a mediator, the TTA-UC QY with 0.01 mM Nap was a mere 0.15 ± 0.02% (Figure 3a and Table 1, entry 5).

**Table 1:** TTA-UC Quantum Yields with and without Reabsorption Correction

	TTA-UC system^a^		TTA-UC QY (%)^b^ ^,^ ^c^	
entry	Nap (mM)	BT (mM)	corrected^d^	uncorrected^e^
1	1		12.6 ± 0.3	6.05 ± 0.5
2	0.1	1	12.2 ± 0.3	7.2 ± 0.3
3	0.1		3.38 ± 0.01	2.0 ± 0.05
4	0.01	1	7.8 ± 0.15	5.4 ± 0.1
5	0.01		0.2 ± 0.02	0.15 ± 0.02
6		1	1.5 ± 0.05	1.0 ± 0.1
7	0.1	10	12.5 ± 0.55	6.7 ± 0.3

a All samples contained 25 μM 4CzBN as the sensitizer.

b TTA-UC QY was measured in a 4 × 10 mm cuvette, where the sample was excited across a 10 mm path of cuvette, with emitted light detected in a 90° angle, passing through the 4 mm path to minimize reabsorption of emitted light.

c Averaged over two trials except for entries 1 and 2, which are averaged over four trials with standard deviation as error. UC QY measured using the standard compound Coumarin 153 in aerated ethanol (Φ_F_ = 0.53).^48^ The reported QY values are with respect to the maximum value of 50%.

d After reabsorption correction.

e Without reabsorption correction.

The importance of reabsorption can be illustrated by comparing the measured TTA-UC QY with the reabsorption corrected values (Table 1). For the two-component system with 1 mM Nap, reabsorption accounts for a 52% loss in intensity as the corrected TTA-UC QY is 12.6 ± 0.3% compared to 6.05 ± 0.5% before correction (Table 1, entry 1). For the three-component system with 0.1 mM Nap, reabsorption accounts for 41% signal loss (Table 1, entry 2), whereas with 0.01 mM Nap, the reabsorption loss is only 31% (Table 1, entry 4). The fact that there is still 31% reabsorption in the low-concentration sample can be explained by the absorption overlap of the sensitizer in the emission region (SI, Figure S33).

On increasing the concentration of mediator BT to 10 mM, while maintaining the concentration of sensitizer at 25 μM and annihilator at 0.1 mM, no significant change in the upconversion QY is observed i.e., 6.7 ± 0.3% (Figure 3 and Table 1, entry 7).

To investigate whether the mediator itself is undergoing TTA-UC and contributing to the overall upconversion efficiency, TTA-UC experiments with 1 mM BT and 25 μM 4CzBN were performed. The TTA-UC QY for the 1 mM BT and 25 μM 4CzBN systems is approximately 6 times lower (1.0 ± 0.1%) than 1 mM Nap and 25 μM 4CzBN (Table 1, entries 6 and 1, respectively). This low TTA-UC QY is attributed to the ∼6 times lower fluorescence QY of BT (11.9% in toluene, see SI) compared to that of Nap (77% in toluene^48^). The low-emission QY from the mediator, yet high TTA-UC QY from the three-component system suggests that homo-TTA of the mediator is a minor problem, and that BT indeed acts as a mediator for energy transfer from sensitizer to annihilator.

To understand the generality of the mediator approach, we switch the mediator to 1,4-bis­[2-[tris­(1-methylethyl)­silyl]-ethynyl]­benzene, (abbreviated as Ph). The triplet energy of Ph is 2.64 eV,^51^ slightly higher than BT (T_1_ = 2.37 eV) but still in between that of 4CzBN (T_1_ = 2.71 eV) and Nap (T_1_ = 2.12 eV), complying with the requirement for a triplet energy transfer mediator. The concentration of annihilator and sensitizer is maintained at the same level as that of the best TTA-UC system with 1 mM mediator BT, i.e., 0.1 mM Nap and 25 μM 4CzBN. The concentration of Ph was maintained at 1 mM. Under this condition, TTA-UC QY is quite low, 3.0 ± 0.2% (SI, Table S1, entry 10). Yanai and co-workers^51^ reported Ph as an annihilator for TTA-UC in the presence of the 4CzBN sensitizer with a TTA-UC QY of 1%, albeit at a high concentration of 10 mM of the annihilator, suggesting that TET from 4CzBN to Ph might not be as efficient as that of Nap. Nevertheless, given that the TTA-UC system with a Ph mediator shows a higher TTA-UC QY (i.e., 3.0 ± 0.2%, SI, Table S1, entry 10) compared to the TTA-UC QY of the two-component system with only 0.1 mM Nap (i.e., 2.0 ± 0.05 Figure 3 and Table 1, entry 3), indicates that Ph does act as a mediator, but with less efficiency compared to that of BT. Contribution of TTA-UC from Ph itself is also minimized in the mixture of 25 μM 4CzBN, 1 mM Ph, and 0.1 mM Nap as Ph needs to be in higher concentration (10 mM) to undergo efficient TTA-UC, as per the previous report from Yanai and co-workers.^51^ It is evident from Table S1 that BT acts as a better mediator compared to Ph (SI, Table S1, entry 10) under the same concentration of sensitizer and annihilator. Since the absorbance of Ph and BT looks nearly identical (Figure S15, SI), it is unlikely that the lower efficiency of Ph as a triplet mediator arises due to greater reabsorption of upconverted emission by Ph. Instead, it is likely due to slower and less efficient TET^S→M^, as reported previously (*k*_TET_ ∼ 6 × 10^7^ M^–1^s^–1^).^51^

To further investigate the generality of mediated TTA-UC, we explored a three-component system in the visible region. The three-component system consists of commonly used sensitizer platinum­(II) octaethylporphyrin (PtOEP) and annihilator 9-phenyl-10-(2-phenylethynyl)­anthracene (PPE-A)^52^ and mediator 9,10-diphenylanthracene (DPA). The triplet energy of mediator DPA (T_1_ = 1.77 eV)^52^ is lower than that of sensitizer PtOEP (T_1_ = 1.92 eV),^52^ but higher than that of annihilator PPE-A (T_1_ = 1.49 eV)^52^ making DPA an ideal energy transfer mediator. Unlike the low fluorescence quantum yield of the mediator BT (Φ_BT_ = 11.9%), the mediator DPA has a high fluorescence quantum yield in deaerated toluene (close to unity).^28,53^ Also, the sensitizer PtOEP has high triplet energy transfer rate constants (*k*_TET_) to both DPA and PPE-A (close to diffusion limit, ∼10^9^ M^–1^ s^–1^).^52^ However, we find that depending on the concentration of DPA and PPE-A, DPA can act as an effective mediator to enhance the TTA-UC QY from the annihilator PPE-A. The concentration of the sensitizer PtOEP was maintained at 15 μM (corresponding to an absorbance at the excitation wavelength 526 nm ∼ 0.2, refer SI) and that of mediator DPA at 0.1 mM. For the three-component system with 0.01 mM PPE-A, the TTA-UC QY was 13.1 ± 0.6%, while in the absence of the mediator, it was only 3.75 ± 0.05%, highlighting the significance of the mediator molecule in increasing the TTA-UC QY (Table S3, entries 1 and 2 respectively).

However, the TTA-UC QY, in the presence of a mediator, has some contribution from the mediator homo-TTA, as evident from the UC peak centered at 412 nm, corresponding to DPA emission (Figure S42). Importantly, even though the mediator only system, i.e., 0.1 mM DPA with 15 μM PtOEP, showed a similar TTA-UC QY of 13.15 ± 0.35% (Table S3, entry 6), the corresponding UC emission spectra are significantly different (Figure S42). In the presence of the annihilator PPE-A (0.01 mM), the majority of upconversion originates from PPE-A with an overall TTA-UC QY of 13.1 ± 0.6% (Table S3, entry 1). We estimate the TTA-UC QY arising from DPA emission in the three-component system to 1.45 ± 0.05%, by adjusting the TTA-UC emission intensity at 412 nm of the two-component system (i.e., 0.1 mM DPA and 15 μM PtOEP) to that of the three-component system (0.01 mM PPE-A, 0.1 mM DPA, and 15 μM PtOEP). Interestingly, even after increasing the concentration of the mediator to 1 mM, the three-component system (0.01 mM PPE-A, 1 mM DPA, and 15 μM PtOEP) showed only a minor (1.3 times) increase in the UC emission from DPA at 412 nm, (Figure S42) with an overall TTA-UC QY of 15.3 ± 0.2% (Table S3, entry 5).

However, on decreasing the concentration of the annihilator PPE-A, the three-component system (0.001 mM PPE-A, 0.1 mM DPA, and 15 μM PtOEP) exhibited an overall TTA-UC QY of 14.35 ± 0.05% (Table S3, entry 3), with major contribution from the direct UC emission from the mediator DPA, as evident with a 7.5 times increase in UC intensity at 412 nm (Figure S42). This behavior of increased contribution from the mediator occurring at low annihilator concentrations (i.e., 0.001 mM PPE-A) is also observed for the UV-emitting three-component system (0.01 mM Nap, 1 mM BT, and 25 μM 4CzBN), as discussed further below.

### Mechanistic Insights into Mediator-Enhanced TTA

We use nanosecond-to-millisecond transient absorption (nsTA) to gain further insights into the mediator-enhanced TTA-UC process. First, Nap and BT are studied alone with 4CzBN as the sensitizer. Figure 4a,b shows the spectral evolution for Nap (annihilator) and BT (mediator), respectively. At early time, a ground-state bleach (GSB) signal of the sensitizer is observed around 400 nm. The spectra evolve as TET proceeds to populate the annihilator/mediator triplet states. The triplet spectra of Nap show photoinduced absorption (PIA) peaks at 360 and 450 nm, whereas BT has a sharp PIA at 360 nm, but minimal absorption at 450 nm. Kinetic traces from these nsTA experiments are shown in Figure 4d,e. Fitting the kinetic profile according to conventional TTA-UC kinetics (Refs. 54 and 55, see details in SI Section 13) with the TTA rate constant (*k*_TTA_) as the sole fitting parameter indicates that both Nap and BT have similar *k*_TTA_ of about 1 × 10^9^ M^–1^ s^–1^. With *k*_TTA_ and the experimental threshold intensity (*I*_th_), we then extract the triplet lifetimes according to eq 2,

$$I_{\mathrm{th}}=\frac{k_{T}^{2}}{2k_{\mathrm{TTA}}\alpha [\mathrm{Sens}]}$$ 2

where *k*_T_ is the inverse of the triplet lifetime, α is the absorption cross-section of the sensitizer at the excitation wavelength, and [Sens] is the sensitizer concentration.

**Figure 4:**
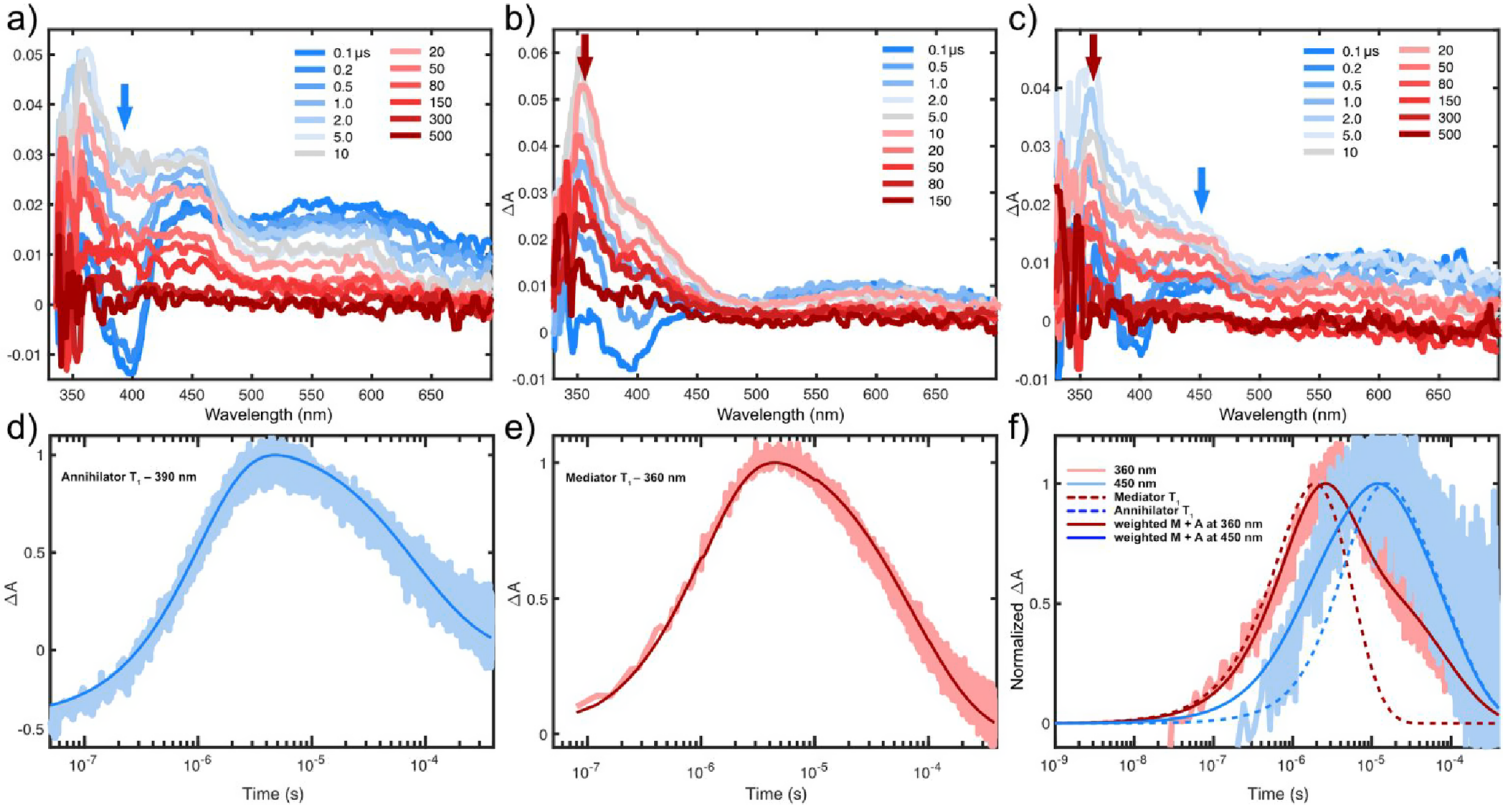
Nanosecond transient absorption spectra at selected time points for (a) Nap, (b) BT, and (c) 0.1 mM Nap and 1 mM BT, all with 25 μM 4CzBN and excitation at 410 nm with a pulse power of 2.3 mJ/pulse. (d,e) Kinetic traces corresponding to the samples in (a–c). (d) Nap at 390 nm (blue arrow in a), (e) BT at 360 nm (red arrow in b), and (f) 0.1 mM Nap with 1 mM BT at 360 (red) and 450 nm (blue). Trace at 360 nm corresponds to majority BT (red arrow in part c) triplet absorption, and traces at 450 nm mostly Nap triplet absorption (blue arrow in part c). Dashed lines are modeled triplet populations, and solid lines are weighted sums of both Nap and BT triplet populations.

We observe that the triplet lifetime of BT (156 μs) is slightly shorter than that of Nap (226 μs), perhaps due to the incorporation of the slightly heavier sulfur atom in the structure.

Importantly, the model works well to reproduce both the kinetic traces (Figure 4d,e), the reabsorption corrected steady-state TTA-UC QYs and intensity ramps for the bimolecular systems (Figures S59 and S60). We then extend the model to a three-component system where we consider: triplet energy transfer from the sensitizer to both mediator and annihilator; triplet energy transfer from mediator to annihilator; homo-TTA between either two triplet excited mediators or two triplet excited annihilators; as well as hetero-TTA between one triplet excited mediator and one triplet excited annihilator. The full kinetic model is described in the SI.

nsTA measurements of the three-component system (25 μM 4CzBN, 1 mM BT, and 0.1 mM Nap) are shown in Figure 4c,f. As seen in Figure 4c, the spectra of the three-component system resemble a mix of the triplet spectra of Nap and BT. However, kinetic traces at the wavelengths dominated by annihilator Nap (450 nm) and mediator BT (360 nm) indicate that the annihilator and mediator triplets are populated at different times, as shown in Figure 4f. Specifically, an initial rise of the mediator triplet is followed by a delayed population of the annihilator triplet state, as shown in Figure 4f. The annihilator signal at 450 nm peaks at ∼20 μs, whereas the mediator triplet peaks already at ∼5 μs. From the spectra in Figure 4c, it is clear that any GSB from the sensitizer is gone by 2 μs, suggesting that TET from mediator to annihilator is the main population route of the annihilator on the time scale 5–20 μs. These trends can be reproduced with the kinetic model using rate constants (*k*_TTA_ and *k*_TET_) for the mediator and annihilator, as determined from the individual measurements.

Importantly, although the annihilator concentration is relatively low (0.1 mM), TET outcompetes the intrinsic triplet decay of the mediator BT by an order of magnitude. With a diffusion-limited mediator to annihilator TET rate constant (∼1 × 10^9^ M^–1^ s^–1^), further reduction of the annihilator concentration to 0.01 mM would still result in >60% TET efficiency for a mediator triplet lifetime of 156 μs. Hence, a long-lived mediator triplet state is crucial for achieving efficient TET and TTA-UC with low annihilator concentrations.

We also follow the delayed UC emission and find that it peaks at 10 μs, as shown in Figure 5a. Since UC emission will result from the population of either the mediator or annihilator singlet excited state (S_1_), we compare the UC time profile to our model. The kinetic model predicts an early population of mediator S_1_, maximizing at 1 μs. Furthermore, in the steady-state UC measurements, we only observe annihilator fluorescence, leading us to conclude that the UC signal arises from annihilator emission. Without hetero-TTA (i.e., TTA between one triplet BT and one triplet Nap) in the model, the annihilator S_1_ reaches a maximum at a longer time scale than that observed (16 μs). Introducing a hetero-TTA channel with *k*_TTA‑hetero_ = 2*k*_TTA‑homo_ yields an excellent match with the time profile, as shown in Figure 5a. At this stage, it is unclear why hetero-TTA would have a larger rate constant than homo-TTA; perhaps it is due to the slightly larger driving force for TTA when a higher-energy mediator triplet is consumed to populate the annihilator singlet. Cao et al.^40^ observed a lowering of the excitation intensity dependence on the UC emission in three-component systems, yet no satisfactory explanation has been presented. However, their observations are in line with an increased rate constant of hetero-TTA compared with homo-TTA, as suggested by our model.

**Figure 5:**
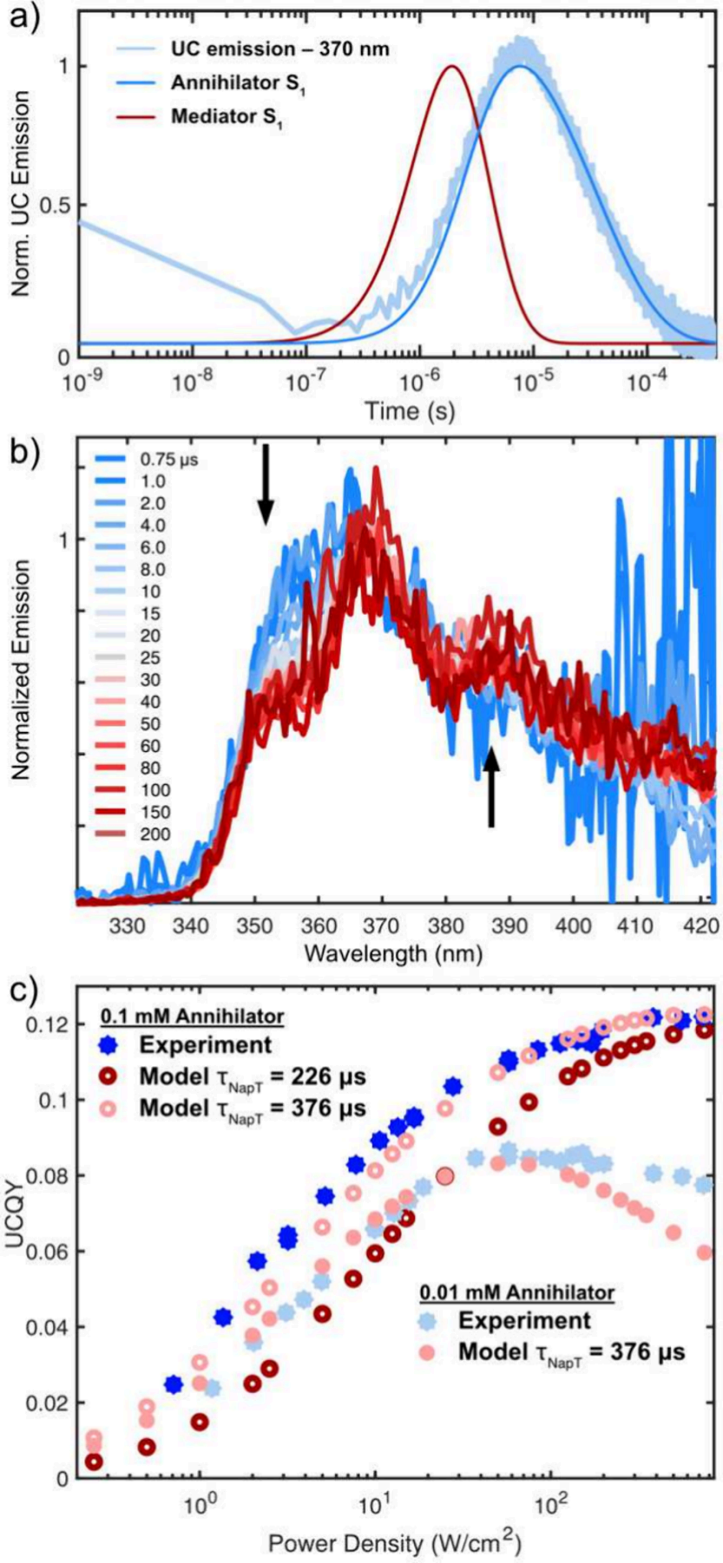
(a) Kinetic trace of upconverted emission detected at 370 nm for a sample with 0.1 mM Nap, 1 mM BT, and 25 μM 4CzBN, excited at 410 nm with a pulse power of 2.3 mJ/pulse. Solid lines indicate the normalized population dynamics of mediator and annihilator singlet states (S_1_). (b) Area-normalized upconversion emission at selected time points. (c) Experimental (solid blue) and modeled (red) upconversion quantum yields (UCQY) as a function of excitation power density for (i) 25 μM 4CzBN, 1 mM BT, and 0.1 mM Nap and (ii) 25 μM 4CzBN, 1 mM BT, and 0.01 mM Nap. The discrepancy between modeled and experimental results at low excitation power densities is removed by extending the triplet lifetime of Nap from 225 to 376 μs.

As an alternative to hetero-TTA, singlet annihilators could be populated from singlet energy transfer (SET) from mediator singlets. However, to fit the experimental UC time profile, a SET rate constant >10^13^ M^–1^ s^–1^ is required, which is far beyond the diffusion limit. Furthermore, the shape of the annihilator S_1_ kinetics becomes broader than what is observed experimentally, as shown in Figure S66. Additionally, considering the low fluorescence QY of the mediator BT, the TTA-UC QYs would be reduced if the main annihilator S_1_ population pathway was through SET. We thus include only the hetero-TTA channel in our further analysis.

The S_1_ population profiles in Figure 5a indicate that the upconversion emission might evolve over time, being dominated by mediator emission at early (∼1 μs) times and annihilator emission at longer (∼10 μs) times. Even though the emission quantum yield of BT is significantly lower, making it difficult to measure weak BT emission, we are able to track such spectral evolution in the UC emission of the 0.01 mM Nap, 1 mM BT, and 25 μM 4CzBN samples, as shown in Figure 5b. At higher Nap concentrations (0.1 mM), fast and efficient TET from the mediator to annihilator results in upconverted BT emission too weak for us to detect with the nsTA instrumentation.

The model for the three-component system adequately reproduces both time-resolved and steady-state data (Figures 4f, 5c, and S61). Some discrepancy between the model and experiment is found at lower excitation powers when modeling the TTA-UC QY, shown as dark red circles in Figure 5c. At low excitation powers, the main decay of Nap triplets is through intrinsic triplet decay and not TTA. Hence, the only parameter affecting the model at low excitation power is the triplet lifetime of Nap. By increasing the triplet lifetime from 226 to 376 μs, the model also reproduces the experimental TTA-UC QYs in the low-intensity regime. We attribute this difference in triplet lifetime to variations in the oxygen level between samples.

With an appropriate model, we can now modify the rate constants to gain further mechanistic insights into mediator-enhanced TTA and compare it to previous reports in the literature. It is important to highlight that our model makes no assumption of the singlet and triplet energies; hence, it can be applied to TTA-UC systems in any wavelength region as long as appropriate rate constants are used. For the following discussion, we use the rate constants established for the BT-mediated system with 4CzBN as the sensitizer and Nap as the annihilator.

First, we discuss the role of triplet energy transfer from a sensitizer to a mediator. In 2023, Glaser et al.^36^ demonstrated a mediator-enhanced TTA-UC system using Coulomb interactions to increase the sensitizer to mediator triplet energy transfer rate constant (*k*_TET_^S→M^) beyond the diffusion limit. In Figure 6, we show the effect of varying *k*_TET_^S→M^ values for different systems. In our system with 1 mM mediator and 0.1 mM annihilator, there is little benefit of increasing the triplet energy transfer rate from 10^9^ to 10^10^ M^–1^ s^–1^. However, in a situation where the sensitizer lifetime is 10 times shorter (∼600 ns), there is a 3 times enhancement in TTA-UC QY with the same change. Furthermore, with a larger *k*_TET_^S→M^, the lower mediator concentration can be used to achieve the same TTA-UC QY. Hence, a long sensitizer triplet lifetime or a large *k*_TET_^S→M^ allows one to compose mediated systems with the lower mediator concentration.

**Figure 6:**
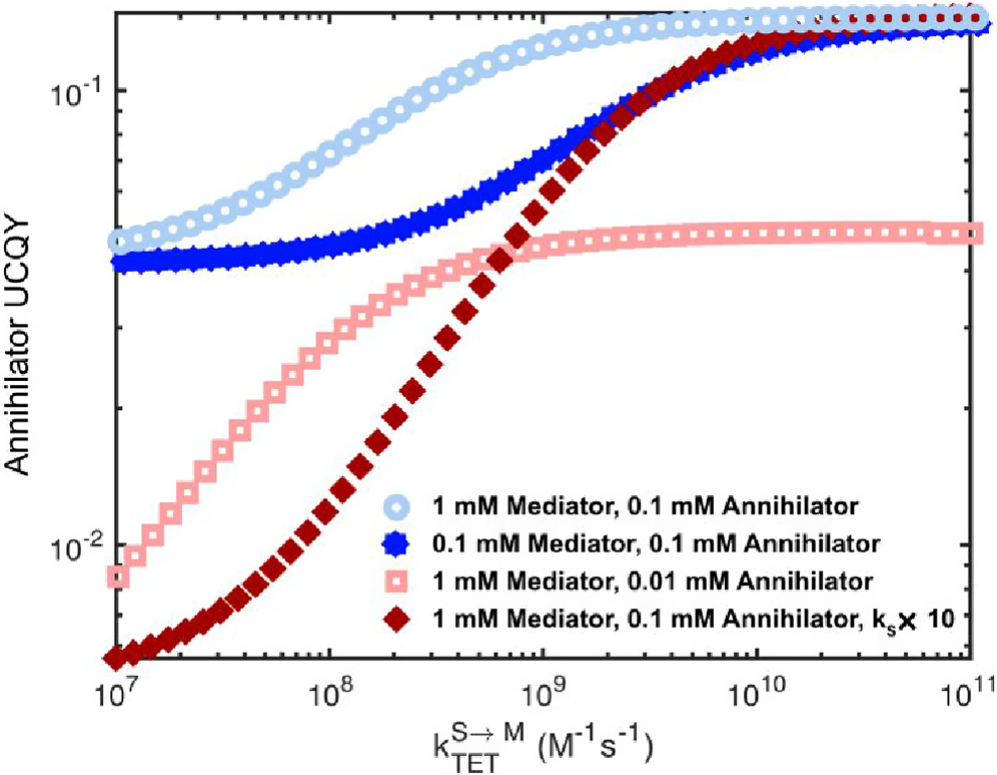
Modeled TTA-UC quantum yield (UCQY) originating from annihilator emission as a function of sensitizer to mediator triplet energy transfer (TET) rate constant (*k*_TET_^S→M^) for four different trimolecular systems. *k*_s_ × 10 refers to the case with a 10 times shorter sensitizer triplet lifetime, here, 600 ns.

Similar analysis, but varying the rate constants for intrinsic triplet decay of the mediator (*k*_T_^BT^) or sensitizer (*k*_T_^S^), the rate constants for hetero or homo-TTA (*k*_TTA_^hetero^ and *k*_TTA_^homo^, respectively) (Figure S63) indicate that the current system is close to optimal, and shorter triplet lifetimes and slower TTA will have a negative effect on the UC efficiency, as can be deduced for the corresponding bimolecular TTA-UC systems.

Now, we turn to discuss the roles of homo-TTA of mediator, homo-TTA of annihilator, and hetero-TTA between a mediator and annihilator. Figure 7a shows the ratio of hetero-TTA to homo-TTA of both mediator and annihilator, as per eq 3:

Ratio=kTTAhetero[A3*][M3*](kTTAhomo[A3*][A3*]+kTTAhomo[M3*][M3*]) 3

With *k*_TTA_^hetero^ = 2 × *k*_TTA_^homo^, the maximum ratio is 1, when the rate of hetero-TTA equals the sum of rate of homo-TTA of the annihilator and mediator, i.e., [^3^ A*] = [^3^ M*]. In solution, we find that hetero-TTA is more important at low annihilator concentrations (0.01 mM annihilator; Figure 7a). Hetero-TTA and TTA-UC QY are maximized when annihilator and mediator triplet concentrations are equal, as predicted by Schmidt and Castellano^42^ (Figures 7a,b and S61). However, in their analysis, their analytical solutions were limited to low excitation intensity regions where triplet decay mainly occurs through intrinsic decay.

**Figure 7:**
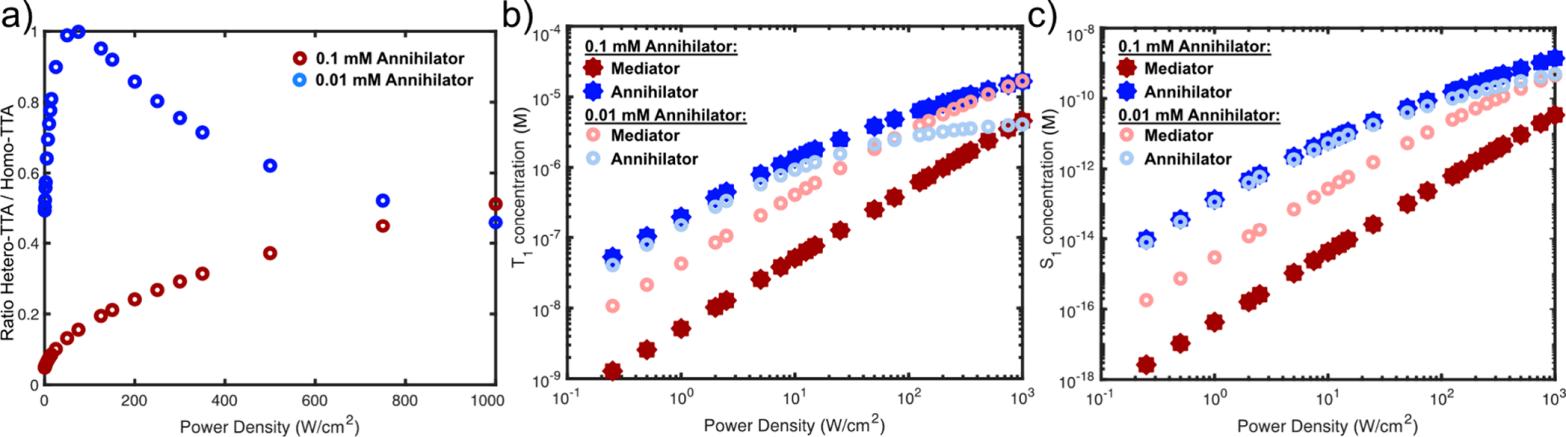
(a) Ratio of hetero-TTA to homo-TTA (sum of annihilator and mediator homo-TTA) as a function of excitation power density for low (0.01 mM, blue) and high (0.1 mM, red) concentrations of annihilator. (b) Modeled T_1_ population of BT mediator (red) and Nap annihilator (blue) as a function of excitation power density for high (0.1 mM, solid) and low (0.01 mM, open) concentrations of Nap annihilator with 1 mM BT mediator and 25 μM 4CzBN. (c) As in (b), but singlet excited state (S_1_) population of mediator (red) and annihilator (blue) species.

As seen in Figure 7b, with a 0.1 mM annihilator, TET is sufficiently efficient to keep [^3^A*] well above [^3^M*]; consequently, annihilator homo-TTA dominates at intensities below 10 000 W/cm^2^ (Figures 7a and S64). On the other hand, for the case of a 0.01 mM annihilator, TET is not as efficient, and [^3^A*] falls below [^3^M*] at around 100 W/cm^2^, as shown in Figure 7b. At higher intensities, the [^3^A*] concentration saturates as the annihilator ground-state population is depleted significantly, and mediator to annihilator TET levels off. Instead, [^3^M*] increases, and hetero-TTA followed by mediator homo-TTA becomes dominant. Due to the low fluorescence quantum yield of the mediator, homo-TTA and formation of [^1^M*] will result in ∼6 times lower TTA-UC QY compared to the same concentration of [^1^A*]. Hence, when [^1^M*] dominates in Figure 7c, it translates to a decrease in the TTA-UC QY due to mediator homo-TTA, as observed experimentally, as shown in Figure 5c. Here, we note that in a case where the mediator fluorescence QY is high, like the case with DPA, the overall TTA-UC QY will remain high, but the emission spectra will resemble that of mediator emission rather than annihilator emission (Figure S42).

It is insightful to compare the crossing point of mediator and annihilator singlet and triplet states populations. For low annihilator concentrations (0.01 mM), [^1^A*] is the majority product until about 750 W/cm^2^, when [^1^M*] crosses over and becomes the dominant product, as shown in Figure 7c. This crossover occurs at much higher excitation densities than where [^3^M*] becomes dominant (Figure 7b), due to efficient hetero-TTA still yielding [^1^A*]. At intensities beyond 1000 W/cm^2^ (and 0.01 mM annihilator), mediator homo-TTA is expected to dominate fully. With a higher annihilator concentration (0.1 mM), the leveling off of [^3^A*] due to annihilator ground-state depletion is slower and requires a significantly higher intensity, beyond what our experimental setup can achieve. However, with a stronger sensitizer absorption, one might approach such regimes also in solution. One should note that the crossover will depend on the concentrations of all three components (sensitizer, mediator, and annihilator) as well as their intrinsic triplet decay rates.

An interesting comparison is to that of a mediator-enhanced TTA-UC system in the solid state using rubrene as the annihilator and tetracene as the mediator, reported by Carrod et al.^46^ They found that hetero-TTA decreased with excitation intensity as the local mediator triplet concentration ([^3^M*]) increased and led to increased mediator homo-TTA. In solution, as discussed above, homo-TTA of the mediator is mostly an issue at low annihilator concentrations.

With the additional insight from the model, it is worth discussing possible drawbacks of mediator-assisted TTA-UC as well. First, going from a two- to a three-component system will increase the complexity of the system, which, in turn, can lead to more difficulties in finding optimal conditions. The system might not be as flexible as a two-component system, e.g., for a two-component system, there is a large window of high excitation powers above *I*_*th*_ in which UC emission is maximized. The only bottleneck arises when ground-state depletion of the sensitizer starts to become significant. For a three-component system, however, this high intensity region with maximal UC yield can be narrower, especially if the mediator fluorescence quantum yield is low, in which case, homo-TTA of the mediator will lead to a decrease in UC emission at higher intensities.

With a mediator, one also introduces another energy transfer step with its associated driving force, potentially decreasing the achievable anti-Stokes shift. However, we see this as a minor drawback, as in most current two-component systems, there is enough energetic offset between sensitizer and annihilator triplet levels to fit a mediator triplet state in between, e.g., as the current case with 4CzBN, Nap and BT. However, it will require designing mediators with appropriate triplet energies.

## Conclusions

Herein, we demonstrate a three-component upconversion system comprising a sensitizer, an annihilator, and a triplet mediator. The triplet mediator ensures efficient triplet energy transfer from the sensitizer to annihilator, allowing us to reduce the annihilator concentration by a factor of 100, which in turn reduces the intrinsic reabsorption of the system from 50% to 31%. Increased TTA-UC QY at low concentration of annihilator can potentially help address the challenges associated with excimer formation at high annihilator concentration.^47,56,57^ Our approach increases the range of possible sensitizer and mediator molecules by not restricting them to charged species with Coulombic interactions or covalently linked dyads. We also present a detailed mechanistic model and evaluate the rate constant for hetero-TTA (TTA between a mediator and an annihilator). To the best of our knowledge, this is the first time a hetero-TTA rate constant has been estimated. Importantly, the rate constant of hetero-TTA appears to be larger than the homo-TTA rate constants, possibly due to a larger energetic driving force for TTA. Overall, this work demonstrates a mechanistic basis to further explore mediator-enhanced TTA-UC systems.

## Supplementary Material



## Data Availability

Data underlying the figures and conclusions in this publication, including the MATLAB code is available at Zenodo data repository with DOI: 10.5281/zenodo.15266985.

## Notes

Additional experimental details, data and details for the synthesis, additional fluorescence and absorption spectra, and details on kinetic modeling (PDF)
