# Correction to “Improving Triplet–Triplet Annihilation Upconversion Output by a Triplet Mediator Approach: Mechanistic Insights on Homo- and Hetero-Annihilation in Three-Component Systems”

**DOI:** 10.1021/jacs.5c22551

**Published:** 2026-01-02

**Authors:** Sunil Kumar Kandappa, Victor Gray

Corrections to the Supporting Information follow. These changes do not affect the data or conclusions in the manuscript.

The correct form of eq 1 in the Supporting Information should read

1 $$\Phi_{UC}=\Phi_{r}\left[\frac{A_{r}}{A_{UC}}\frac{F_{UC}}{F_{r}}\frac{\eta_{UC}^{2}}{\eta_{r}^{2}}\right]$$

The following typographical errors in the Supporting Information are corrected:

a) In the paragraph below eq 1, p 3, of the Supporting Information the line should read as “... *F*_*UC*_ and *F*_*r*_ are ...” The corrected text is as follows:
  In this equation, *A*_*r*_ and *A*_*UC*_ are the absorbance of reference compound and upconversion sample at the excitation wavelength, *F*_*UC*_ and *F*_*r*_ are the integrated emission intensity of upconversion sample and reference compound, η_*r*_ and η_*UC*_ are the refractive index of solvent used for reference sample and upconversion sample, respectively. UC QY reported to the maximum value of 50%.
b) In the kinetic modeling section, p 53 of the Supporting Information, the line should read as “... The code is available to download ...” The corrected text is as follows:
  Kinetic modeling was done using MATLAB. The code is available to download at the Zenodo repository (http://zenodo.org) with DOI: 10.5281/zenodo.15266985. The differential equations describing the trimolecular system used for the modeling are described below:

The corrected Supporting Information is published with this Correction.

## Supplementary Material



## Notes

Additional experimental details, data and details for the synthesis, additional fluorescence and absorption spectra, and details on kinetic modeling (PDF)

