# Supplementary material for: Correction to “Improving Triplet–Triplet Annihilation Upconversion Output by a Triplet Mediator Approach: Mechanistic Insights on Homo- and Hetero-Annihilation in Three-Component Systems”
Source: J Am Chem Soc. 2026 Jan 2;148(2):2820. doi: 10.1021/jacs.5c22551 (PMC12833834; doi:10.1021/jacs.5c22551)
Supplement: Supplementary file 1 [file ja5c22551_si_001.pdf]

Supporting Information

Improving Triplet–Triplet Annihilation Upconversion Output by a Triplet Mediator Approach: Mechanistic Insights on Homo and Hetero-Annihilation in Three-Component Systems

Sunil Kumar Kandappa, Victor Gray*
Department of Chemistry, Ångström Laboratory, Uppsala University, Box 532, SE-751 20 Uppsala, Sweden

Table of Contents

1. General Experimental details 2
2. Photophysical experiments 2
3. Synthesis 3
3.1. Synthesis of Nap; 1,4-bis[2-[tris(1-methylethyl)silyl]ethynyl]naphthalene 3
3.2. Synthesis of Ph; 1,4-bis[2-[tris(1-methylethyl)silyl]ethynyl]benzene 4
3.3. Synthesis of 3,6-dibromo-2-fluorobenzaldehyde 5
3.4. Synthesis of 3,6-dibromo-2-[(1,1-dimethylethyl)thio]benzaldehyde 5
3.5. Synthesis of tosyl azide 6
3.6. Synthesis of dimethyl (1-diazo-2-oxopropyl)phosphonate 7
3.7. Synthesis of 1,4-dibromo-2-[(1,1-dimethylethyl)thio]-3-ethynylbenzene 7
3.8. Synthesis of 4,7-dibromobenzo[*b*]thiophene 8
3.9. Synthesis of BT; 4,7-bis[2-[tris(1-methylethyl)silyl]ethynyl]benzo[*b*]thiophene 8
3.10. Synthesis of Synthesis of 9-Bromo-10-phenylanthracene 9
3.11. Synthesis of 9-Phenyl-10-(2-phenylethynyl)anthracene 10
4. TTA-UC quantum yield data for 4CzBN, Nap/BT system 12
5. Plot of integrated area of upconverted emission intensity vs laser energy for 4CzBN, Nap/BT system 12
6. UV-vis absorption spectra of upconversion sample 4CzBN, Nap/BT system 16
7. TTA-UC quantum yield data for PtOEP, PPE-A/DPA system 20
8. Plot of integrated area of upconverted emission intensity vs laser energy for PtOEP, PPE-A/DPA system 21
9. UV-vis absorption spectra of upconversion sample PtOEP, PPE-A/DPA system 24
10. Determination of emission quantum yield of BT 28
11. Stern-Volmer plot 29
12. NMR data 37
13. Kinetic Modelling 53
13.1. Figures from kinetic modelling 56
13.2. Regarding Mediator to Annihilator Singlet Energy Transfer 58
14. References 59

1. General Experimental details

All glassware was dried in an oven at 110 °C before reaction unless otherwise mentioned where dry glassware was not needed. All reactions were performed under argon atmosphere using standard Schlenk-line technique unless otherwise mentioned. For dry solvents, THF and toluene were collected from *Pure Solve MD7* solvent purification system and used as such without further drying. All the chemicals are used as such received from vendors without further purification. NMR experiment performed with Jeol Eclipse+ 400 spectrometer for ^{1}H-400 MHz, ^{13}C-101MHz spectra at room temperature. The chemical shift values were reported in parts per million (PPM or δ) referenced to the residual solvent signal. For $CDCl_3$ it is 7.26 PPM for ^{1}H-NMR and 77.16 PPM for ^{13}C NMR.[1] NMR spectra of all compounds are recorded in $CDCl_3$.The abbreviations s, singlet; d, doublet; t, triplet; q, quartet; m, multiplet; and brs, broad singlet are used to describe multiplicities.

For high-resolution mass spectrometry (HRMS) experiments, the compounds were dissolved in aceotinitrile (ACN) and mixed 1:1 with 2,5-dihydroxy benzoic acid (DHB) matrix solution (35mg/ml in 50% ACN/%0.01TFA) and 1 µl of mixed solution from each compound was spotted on MTP 384 ground steel target plates (Bruker Daltonics) and left for drying at room temperature. HR-MS experiments were performed in positive ionization mode using a matrix-assisted laser desorption/ionization Fourier-transform ion cyclotron resonance (MALDI-FTICR) (7T solariX XR-2ω, Bruker Daltonics) mass spectrometer equipped with a Smartbeam II 2 kHz laser. The instrument was tuned for optimal detection of compounds (m/z 200–2000) in both polarities using the quadrature phase detection (QPD) (2ω) mode and methods were calibrated externally with red phosphorus over an appropriate mass range for each compound analysis. Either M^+ or $[M+H]^+$ ions were detected for each compound and ppm errors were calculated. Molecular ion m/z values of each compound were calculated using IsotopePattern software (Bruker Daltonics). Molecular isotopic distributions of each molecule were also checked for validating the identity of the molecule. For the MALDI analysis of compound **19**, 9-Bromo-10-phenylanthracene and **21**, 9-Phenyl-10-(2-phenylethynyl)anthracene, the respective compounds were dissolved in dichloromethane and spotted on MTP 384 ground steel target plates as mentioned above (without matrix).

Reactions were monitored by thin layer chromatography technique. Column chromatography techniques were used to isolate the product after the chemical reaction. Silica gel 200-400 mesh used for column chromatography. Freshly prepared lithium diisopropylamide solution[2] was used whenever required. The chemicals 1,4-dibromo-2-fluorobenzene, dimethyl(2-oxopropyl)phosphonate, sodium-2-methyl-2-propanethiolate, CuI, PPh3, $Pd(PPh_3)2Cl_2$, triisopropylsilylacetylene, DMF, tosyl chloride, sodium azide, potassium carbonate, diphenyl anthracene, platinum octaethylporphyrin, 9,10-dibromoanthracene, phenylboronic acid and 9-bromo-10-phenylanthracene were purchased from Sigma-Aldrich. Gold(I) chloride was purchased from abcr. For the synthesis involving Nap, BT and Ph standard procedure[3] of Sonogashira coupling was followed. BT was synthesized by following the reported procedure.[4]

2. Photophysical experiments

All experiments involving triplet-triplet annihilation upconversion (TTA-UC) were performed in deaerated toluene. Samples were prepared inside the glovebox under argon atmosphere (with H_2O and O_2 level below 0.1 ppm). For all experiments involving TTA-UC and Stern-Volmer quenching same stock solution of sensitizer, annihilator and mediator was used to minimize the error associated with the sample preparation. Toluene was deaerated by using standard technique of freeze-pump-thaw (5 cycles). The solution of upconversion mixture was prepared inside the glovebox in a cuvette with 4 mm path length in

one side and 10 mm path length in the opposite side. For TTA-UC QY measurement, UV-vis absorption was recorded through 4 mm path of cuvette and converted to the absorbance that correspond to 10 mm pathlength. For TTA-UC QY determination, sample was excited across 10 mm path of cuvette with emitted light detected in 90° angle, passings through the 4 mm path to minimize reabsorption of emitted light. For the UC QY determination, absorbance across 10 mm path length (excitation path) was considered. The steady state UV-vis absorption spectra were performed using Agilent Cary 60 spectrometer. Steady state fluorescence spectra and emission decay kinetics were recorded using FS5 Spectrofluorometer from Edinburgh Instruments. Upconversion experiments were performed using FLS1000 Photoluminescence Spectrometer from Edinburgh Instruments. For the upconversion in the UV region, an external 405 nm CW laser (MPN:X-III-405, 200 mW, Edinburgh Instruments) with a spot size of 0.00012 cm^2 was used. For the upconversion in the visible region, 526 nm CW laser (Edinburgh Instruments) with a spot size 0.0026 cm^2 was used. Upconversion quantum yield Φ_{UC} was determined by the relative method[5,6] using the standard compound Coumarin 153 in aerated ethanol ($\Phi_F = 0.53$)[7] or Rhodamine 6G in aerated ethanol ($\Phi_F = 0.95$)[8] as per the Equation 1, below. In this equation, A_r and A_{UC} are the absorbance of reference compound and upconversion sample at the excitation wavelength, F_{UC} and F_r are the integrated emission intensity of upconversion sample and reference compound, η_r and η_{UC} are the refractive index of solvent used for reference sample and upconversion sample respectively. UC QY reported to the maximum value of 50%.

$$\Phi_{UC} = \Phi_r \left[\frac{A_r}{A_{UC}} \frac{F_{UC}}{F_r} \frac{\eta_{UC}^2}{\eta_r^2}\right] \dots\dots\dots\dots (1)$$

TTA-UC QY values are reported after relative method without any correction as well as with correction for reabsorption of emitted light by annihilator. Previously reported method[9–11] was employed to correct for reabsorption where the fluorescence spectra of a dilute solution of annihilator is normalized to the upconverted emission spectra at a wavelength in the lower energy region of emission spectra with minimal reabsorption.

The nanosecond transient absorption (nsTA) experiments were performed on a flash-photolysis setup using a Nd:YAG laser (Ekspla, NT342B laser) as the source of excitation of the samples with an OPO set at 410 nm having energies of 2 mJ/pulse. The spectrometer (LP920, Edinburgh Instruments) comprises a pulsed 450 W ozone-free Xe arc lamp, a symmetrical Czerny-Turner monochromator (TMS300) with 5 nm bandwidth and detectors for both single kinetic traces (LP900 photomultiplier, with Tektronix TDS3012C oscilloscope) and entire spectra (Andor SH720 ICCD camera).

3. Synthesis

3.1. Synthesis of Nap; 1,4-bis[2-[tris(1-methylethyl)silyl]ethynyl]naphthalene

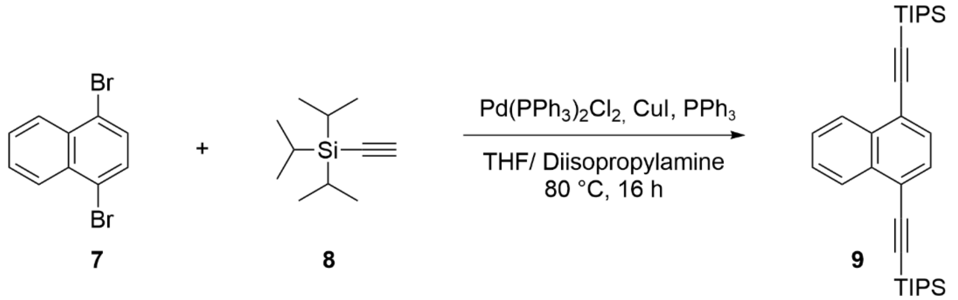

Procedure for this synthesis was adapted from the previous report[3]. A 250 mL two neck round bottomed flask was charged with 1, 4-dibromo naphthalene (1.44 g, 5.04 mmol, 1 equiv), CuI (60.5mg, 0.317 mmol, 0.06 equiv), PPh_3(1.44 mg, 0.317 mmol, 0.06 equiv) and $Pd(PPh_3)_2Cl_2$ (80.06 mg, 0.114 mmol, 0.02 equiv). The flask was purged with argon. Subsequently, dry THF (40 mL) and diisopropyl amine (36 mL) were

added under argon atmosphere. The reaction mixture was stirred at 80 °C for 5 minutes. The reaction mixture changed to orange colour. This was followed by the dropwise addition of triisopropylsilylacetylene under argon atmosphere (4.36 mL, 18.13 mmol, 3.6 equiv). It was further stirred at 80 °C for 16 h. Reaction was monitored by TLC. After the completion of reaction, the reaction mixture was cooled down to room temperature. Solvent was evaporated under reduced pressure in rotary evaporator. Crude material was dissolved in DCM (30 mL) and added DI water (30 mL). Organic layer separated and aqueous layer was further extracted with DCM (3 × 30 mL). Combined organic layer was dried with Na_2SO_4 (anhydrous) and concentrated under reduced pressure. It was further purified by column chromatography.

Column chromatography: Crude material was adsorbed over ~ 5 mL of silica. Slurry of silica (100 mL) in heptane was loaded into a column. To the silica packed column, crude material adsorbed over silica was added. Column was further eluted with 400 mL of 100 % heptane. Eluent was collected as 25 mL fraction in a test tube. Two sets of fractions were collected. First set of fractions from 9-12 had some nonpolar impurity as seen from TLC. Second set from 9-12 was pure by TLC. Impure fractions were combined and subjected to second column chromatography under the same condition mentioned above. Pure fractions from first and second column chromatography combined and concentrated to get pure product (2.12 g, yield = 88%).

Characterization: White solid, R_f: 0.8 in 100% heptane. ^{1}H NMR (400 MHz, $CDCl_3$): δ 8.39 (m, 2H), 7.65 – 7.57 (m, 4H), 1.19 (m, 42H). ^{13}C NMR (101 MHz, $CDCl_3$): δ 133.35, 130.29, 127.32, 126.76, 121.91, 104.89, 97.93, 18.92, 11.54. MALDI-HRMS *m/z*: Mass calculated for [M^+] ion for $C_{32}H_{48}Si_2$; theoretical = 488.3295, observed = 488.3284, |Δppm| = 2.2 ppm.

3.2. Synthesis of Ph; 1,4-bis[2-[tris(1-methylethyl)silyl]ethynyl]benzene

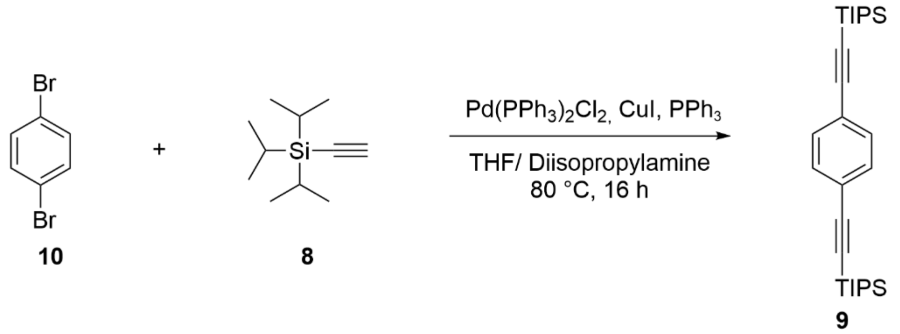

Procedure for this synthesis was adapted from previous report[3]. A 250 mL two neck round bottomed flask was charged with 1, 4-dibromo benzene (1.44 g, 6.10 mmol, 1 equiv), CuI (69.75 mg, 0.366 mmol, 0.06 equiv), PPh_3 (96 mg, 0.366 mmol, 0.06 equiv) and $Pd(PPh_3)_2Cl_2$ (86 mg, 0.122 mmol, 0.02 equiv). The mixture was purged with argon. Subsequently, dry THF (40 mL) and diisopropyl amine (36 mL) were added under argon atmosphere. The reaction mixture was stirred at 80 °C for 5 minutes. The reaction mixture changed to orange colour. Then added triisopropyl silylacetylide (5.28 mL, 21.97 mmol, 3.6 equiv) under argon atmosphere. It was further stirred at 80 °C for 16 h. Reaction was monitored by TLC. After the completion of reaction, the reaction mixture was cooled down to room temperature. Solvent was evaporated under reduced pressure in rotary evaporator. Crude material was dissolved in DCM (30 mL) and added DI water (30 mL). Organic layer separated and aqueous layer was further extracted with DCM (3 × 30 mL). Combined organic layer was dried with Na_2SO_4 (anhydrous) and concentrated under reduced pressure. It was further purified by column chromatography.

Column chromatography: Crude material was adsorbed over ~ 5 mL of silica. Slurry of silica (100 mL) in pentane was loaded into a column. To the silica packed column, crude material adsorbed over silica was added. Column was further eluted with 400 mL of 100 % pentane. Eluent was collected as 25 mL fraction in a test tube. Fractions 8-20 had product along with nonpolar impurity. It was further subjected to the second column chromatography under the condition mentioned above to get pure product. (2.2 g, yield = 82%).

Characterization: White solid, R_f: 0.8 in 100% pentane. ^{1}H NMR (400 MHz, $CDCl_3$): δ 7.39 (s, 4H), 1.13 (s, 42H). ^{13}C NMR (101 MHz, $CDCl_3$): δ 131.92, 123.50, 106.78, 92.81, 18.81, 11.45. MALDI-HRMS *m/z*: Mass calculated for $[M^+]$ ion for $C_{32}H_{48}Si_2$; theoretical = 438.3138, observed = 438.3131, |Δppm| = 1.6 ppm.

3.3. Synthesis of 3,6-dibromo-2-fluorobenzaldehyde

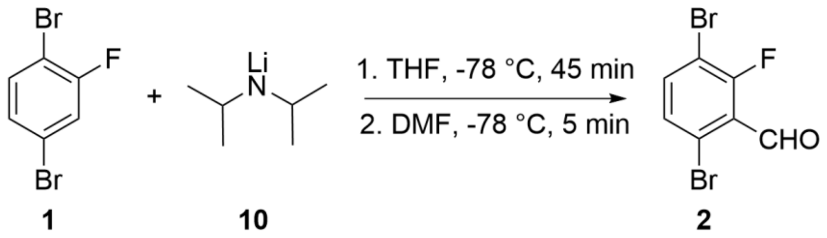

Procedure for this synthesis was adapted from previous report[12]. For the synthesis of aldehyde derivative freshly prepared LDA solution was prepared. For the preparation of LDA solution, a 50 mL round bottomed flask was charged with 10 mL of dry THF and 2.5 mL of diisopropyl amine (freshly distilled over CaH_2). The mixture was cooled to -78 °C and added nBuLi (2.5 M in hexane). It was stirred for about a minute and used as such for the next step.

A 100 mL Schlenk flask was charged with 1,4-dibromo-2-flouro benzene (4 g, 15,75 mmol, 1 equiv) and flushed with argon. Then added 40 mL of dry THF and the mixture was cooled to -78 °C in liquid nitrogen /acetone bath. Subsequently, freshly prepared LDA solution was added (15,91 mL, 1 M, 15,91 mmol, 1.01 equiv) and stirred at the same temperature for 45 min. Afterwards DMF (3,05 mL, 39,39 mmol, 2.5 equiv) was added. The mixture was further stirred for 5 min and then quenched with 4 mL of DI water. The reaction mixture was allowed to warm to room temperature. Further, 10 mL of DI water was added. Organic layer was separated and aqueous layer was extracted with ethyl acetate (3 × 20 mL). Combined organic layer was washed with brine solution and dried with Na_2SO_4 (anhydrous) followed by filtration and concentration under reduced pressure. To the mixture, n-heptane was added and the slurry formed was cooled to -70 °C and the solid formed was filtered. The filtrate obtained was further cooled to -77 °C and the solid material formed was again filtered. Filtered solid material (yellow, fluffy solid) was combined and used as such without further purification for the next step described below. (Crude mass = 4.3 g, crude yield = 96%).

3.4. Synthesis of 3,6-dibromo-2-[(1,1-dimethylethyl)thio]benzaldehyde

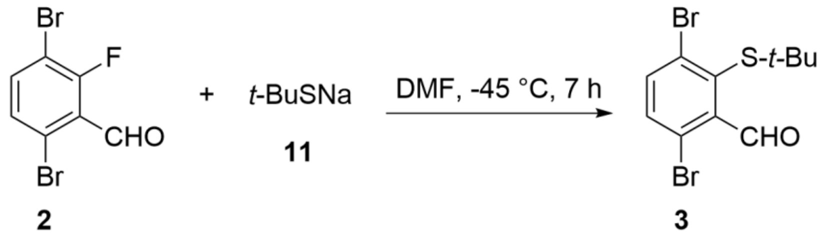

Procedure for this synthesis was adapted from previous report[4]. A 50 mL round bottomed flask was charged with 3,6 dibromo-2-flourobenzaldehyde (4.0 g, 14,19 mmol, 1.0 equiv) and flushed with argon. Then added 20 mL of dry DMF. Another 100 mL Schlenk flask was charged with sodium-2-methyl-2-propanethiolate, *t*-BuSNa (1.75 g, 15.61 mmol, 1.1 equiv) and flushed with argon. Then added 12 mL of dry DMF and cooled to -45 °C (in acetonitrile/liquid nitrogen mixture). To this mixture previously prepared solution of 3,6 dibromo-2-flourobenzaldehyde in 20 mL of dry DMF was added at -45 °C. The reaction mixture was further stirred at the same temperature for 7 h. Reaction was monitored by TLC. Upon completion of reaction, it was poured into saturated solution of NH_4Cl (20 mL). Then added 10 mL of diethyl ether. Organic layer was separated. Aqueous layer was extracted with diethyl ether (3 × 20 mL). Combined organic layer was dried with Na_2SO_4 (anhydrous) and concentrated under reduced pressure. It was further purified by column chromatography.

Column chromatography: Crude material was adsorbed over ~ 10 mL of silica. Slurry of silica (90 mL) in pentane was loaded into a column. To the silica packed column, crude material adsorbed over silica was added. Column was further eluted with 100% pentane and DCM/pentane mixture. Eluent was collected as 25 mL fraction in a test tube. Following solvent mixtures were eluted.
300 mL of 100 % pentane, 200 mL of 2.5 % DCM/pentane and 200 mL of 5 % DCM/pentane. Clean fractions from 19-25 were concentrated to get pure product. (4.0 g, yield = 80 %).

Characterization: Yellow solid, R_f: 0.4 in 20% DCM in pentane. ^1H NMR (400 MHz, $CDCl_3$): δ 7.39 (s, 4H), 1.13 (s, 42H). ^{13}C NMR (101 MHz, $CDCl_3$): δ 192.39, 143.53, 137.53, 136.82, 136.36, 134.63, 119.89, 52.71, 31.64. MALDI-HRMS *m/z*: Mass calculated for [M^+] ion for $C_{32}H_{48}Si_2$; theoretical = 349.8976, observed = 349.8972, |Δppm | = 1.1 PPM.

3.5. Synthesis of tosyl azide

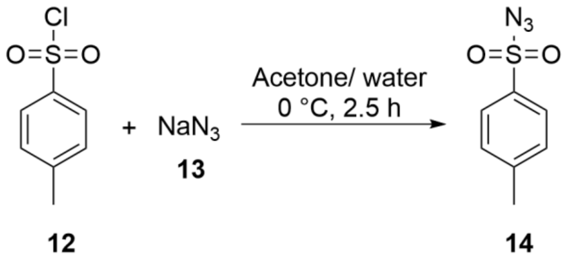

Procedure for this synthesis was adapted from previous report[13]. A 100 mL round bottomed flask was charged with 4 g of tosyl chloride. Then added 25 mL of acetone and 25 mL of water. The mixture was cooled to 0 °C in an ice bath. Subsequently sodium azide was added portion wise. It was further stirred at the same temperature for 2.5 h. The rection mixture was concentrated to the half of initial volume under reduced pressure. Then added 20 mL of diethyl ether. Organic layer was separated and the aqueous layer was extracted with diethyl ether (3 × 20 mL). Combined organic layer was dried with Na_2SO_4 (anhydrous) and concentrated under reduced pressure in rotary evaporator with maintaining the temperature of water bath at room temperature. Crude material (pale yellow solid) was taken as such for the next step described below. (Crude mass = 4.0 g, crude yield = 96%).

3.6. Synthesis of dimethyl (1-diazo-2-oxopropyl)phosphonate

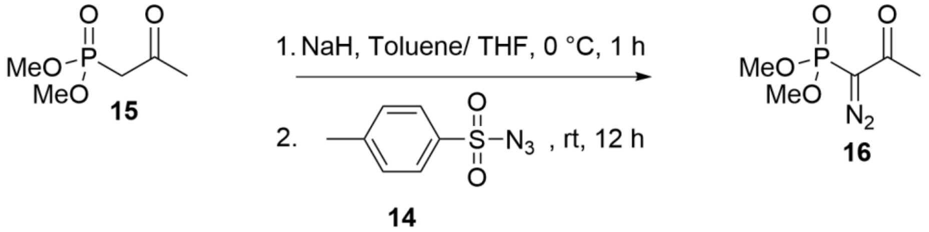

Procedure for this synthesis was adapted from previous report[14]. A 100 mL Schlenk flask was charged with NaH (722 mg, 60% dispersion in mineral oil, 18.06 mmol, 1.5 equiv). Then added 25 mL of toluene and 7 mL of THF. The mixture was cooled to 0 °C. Subsequently dimethyl acetylmethylphosphonate (2 g, 12.04 mmol, 1 equiv) was added to the mixture at the same temperature. The mixture was stirred for 1 h at 0 °C. Afterwards, tosyl azide (2.37 g, 12.04 mmol, 1 equiv) was added and the mixture was further stirred for 12 h at rt. Then the mixture was filtered over Celite pad and the filtrate was concentrated under reduced pressure in rotary evaporator with maintaining the temperature of water bath at room temperature. Crude material (orange oily liquid) was taken as such for the next step described below. (Crude mass = 2.3 g, crude yield = 99%).

3.7. Synthesis of 1,4-dibromo-2-[(1,1-dimethylethyl)thio]-3-ethynylbenzene

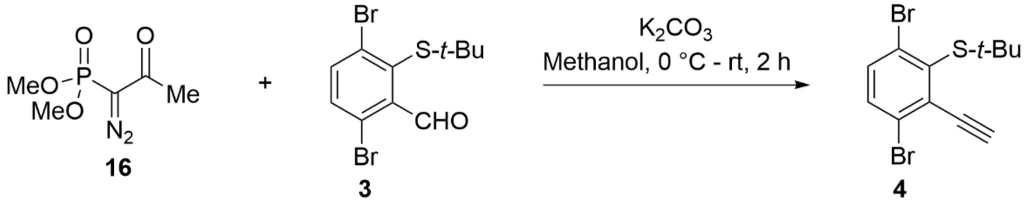

Procedure for this synthesis was adapted from previous report[4]. A 100 mL round bottomed flask was charged with aldehyde derivative (4.57 g, 12.98 mmol, 1.2 equiv) and added 50 mL of methanol. Aldehyde derivative was made sure to dissolve completely in methanol. Another 250 mL Schlenk flask was charged with dimethyl acetylmethylphosphonate (4.99 g, 25.96 mmol, 2 equiv) and added 60 mL of methanol. Subsequently, potassium carbonate was added (5.38 g, 38.93 mmol, 3 equiv) and the mixture was cooled to 0 °C in ice bath. To this mixture, previously prepared solution of aldehyde derivative in 50 mL of methanol was added at 0 °C. The mixture was allowed to warm to rt. and stirred at the same temperature for 2 h. Reaction was monitored by TLC. Upon completion of reaction the mixture was poured into DI water (20 mL) and added 20 mL of DI water. Organic layer was separated and aqueous layer was extracted with diethyl ether (3x 10 mL). Combined organic layer was washed with brine solution and dried over Na_2SO_4 (anhydrous). It was concentrated under reduced pressure in rotary evaporator and purified by column chromatography.

Column chromatography: Crude material was adsorbed over ~ 10 mL of silica. Slurry of silica (200 mL) in pentane was loaded into a column. To the silica packed column, crude material adsorbed over silica was added. Column was further eluted with 100% pentane and DCM/pentane mixture. Eluent was collected as 25 mL fraction in a test tube. Following solvent mixtures were eluted.

100 mL of 100 % pentane, 200 mL of 2.5 % DCM/pentane, 200 mL of 5 % DCM/pentane, 200 mL of 7.5 % DCM/pentane, 200 mL of 10 % DCM/pentane, 200 mL of 12.5 % DCM/pentane. Clean fractions from 16-36 were concentrated to get pure product. (3.4 g, yield = 75 %).

Characterization: White solid, R_f: 0.8 in 20% DCM in pentane. ^1H NMR (400 MHz, $CDCl_3$): δ 7.54 (d, J = 8.6 Hz, 1H), 7.45 (d, J = 8.7 Hz, 1H), 3.65 (s, 1H), 1.42 (s, 10H). ^{13}C NMR (101 MHz, $CDCl_3$): δ 138.93, 134.06,

134.02, 133.92, 133.59, 126.13, 87.27, 82.72, 53.06, 32.00. MALDI-HRMS *m/z*: Mass calculated for [M^+] ion for $C_{32}H_{48}Si_2$; theoretical = 345.9026, observed = 345.9021, |Δppm| = 1.4 ppm.

3.8. Synthesis of 4,7-dibromobenzo[*b*]thiophene

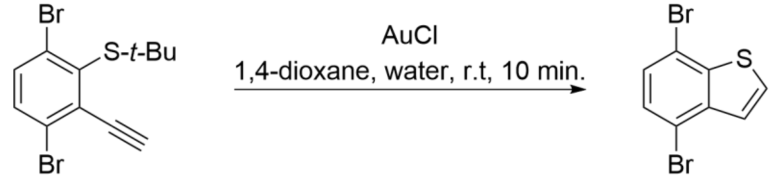

4 **5**

Procedure for this synthesis was adapted from previous report.[4] A 100 mL round bottomed flask was charged with dibromo thiol derivative (3.38 g, 9.71 mmol, 1 equiv). Then added 1,4-dioxane (30 mL) and DI water (6 mL). Subsequently gold chloride, AuCl (90.27 mg, 0.3884 mmol) was added. The reaction mixture was stirred at rt for 10 min. Reaction was monitored by TLC. Upon completion of reaction, solvent was evaporated in rotary evaporator and the crude material was purified by column chromatography.

Column chromatography: Crude material was adsorbed over ~ 10 mL of silica. Slurry of silica (100 mL) in pentane was loaded into a column. To the silica packed column, crude material adsorbed over silica was added. Column was further eluted with 600 mL of 100% pentane. Clean fractions from 9-19 were concentrated to get pure product. (2.6 g, yield = 92 %).

Characterization: White solid, R_f: 0.8 in 100% pentane. ^{1}H NMR (400 MHz, $CDCl_3$): δ 7.62 – 7.54 (m, 2H), 7.43 (d, J = 8.1 Hz, 1H), 7.35 (d, J = 8.1 Hz, 1H). ^{13}C NMR (101 MHz, $CDCl_3$): δ 142.34, 140.21, 128.79, 128.39, 128.04, 125.55, 116.53, 115.04. MALDI-HRMS *m/z*: Mass calculated for [M^+] ion for $C_{32}H_{48}Si_2$; theoretical = 289.8400, observed = 289.8394, |Δppm | = 2.1 ppm.

3.9. Synthesis of BT; 4,7-bis[2-[tris(1-methylethyl)silyl]ethynyl]benzo[*b*]thiophene

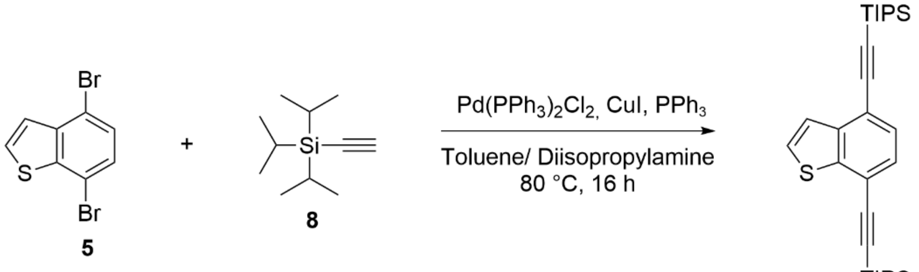

5 **8** **6**

Procedure for this synthesis was adapted from previous report.[3] A 100 mL Schlenk flask was charged with 1, 4-dibromo naphthalene (1.48 g, 5.07 mmol, 1 equiv), CuI (57.92 mg, 0.304 mmol, 0.06 equiv), PPh_3 (79.7 mg, 0.304 mmol, 0.06 equiv) and $Pd(PPh_3)_2Cl_2$ (71.15 mg, 0.101 mmol, 0.02 equiv). The flask was purged with argon. Subsequently, dry toluene (60 mL) and diisopropyl amine (18 mL) were added under argon atmosphere. The reaction mixture was stirred at 80 °C for 5 minutes. The reaction mixture changed to orange colour. This was followed by the dropwise addition of triisopropyl silyl acetylide under argon atmosphere (2.68 mL, 11.15 mmol, 2.2 equiv). It was further stirred at 80 °C for 16 h. Reaction was monitored by TLC. After the completion of reaction, the reaction mixture was cooled down to room temperature. Solvent was evaporated under reduced pressure in rotary evaporator. Crude material was dissolved in ethyl acetate (20 mL) and added DI water (20 mL). Organic layer separated and aqueous layer was further extracted with ethyl acetate (3 × 20 mL). Combined organic layer was dried with Na_2SO_4

(anhydrous) and concentrated under reduced pressure in rotary evaporator. It was further purified by column chromatography.

Column chromatography: Crude material was adsorbed over ~ 10 mL of silica. Slurry of silica (125 mL) in pentane was loaded into a column. To the silica packed column, crude material adsorbed over silica was added. Column was further eluted with 600 mL of 100 % pentane. Eluent was collected as 25 mL fraction in a test tube. Fractions from 4-12 had product along with nonpolar impurity as seen from TLC. Impure fractions were combined and subjected to second column chromatography.
Crude material was adsorbed over ~ 10 mL of silica. Slurry of silica (350 mL) in pentane was loaded into a column. To the silica packed column, crude material adsorbed over silica was added. Column was further eluted with 900 mL of 100 % pentane. Eluent was collected as 25 mL fraction in a test tube. Fractions from 14-32 had product along with nonpolar impurity as seen from TLC. Impure fractions were combined and subjected to third column chromatography.
Crude material was adsorbed over ~ 10 mL of silica. Slurry of silica (500 mL) in pentane was loaded into a column. To the silica packed column, crude material adsorbed over silica was added. Column was further eluted with 900 mL of 100 % pentane. Pure fractions from 21-40 were concentrated to get pure product (2.4 g, yield = 95%).

Characterization: White solid, R_f: 0.7 in 100% pentane. ^{1}H NMR (400 MHz, $CDCl_3$): δ 7.61 – 7.49 (m, 2H), 7.49 – 7.35 (m, 2H), 1.18 (d, J = 3.4 Hz, 42H). ^{13}C NMR (101 MHz, $CDCl_3$): δ 140.86, 128.35, 127.93, 127.39, 124.19, 118.65, 118.18, 105.03, 104.26, 97.85, 96.33, 18.88, 18.84, 11.51, 11.47, 11.43. MALDI-HRMS *m/z*: Mass calculated for [M^+] ion for $C_{32}H_{48}Si_2$; theoretical = 494.2859, observed = 494.2850, |Δppm| = 1.8 ppm.

3.10. Synthesis of Synthesis of 9-Bromo-10-phenylanthracene

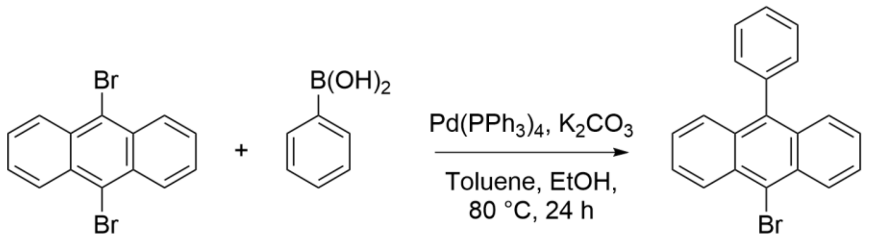

17 **18** **19**

Procedure for this synthesis was adapted from previous report.[15] In a 50 mL round bottomed flask 9,10-dibromoanthracene (400 mg, 1.19 mmol) and phenylboronic acid (145.1 mg, 1.19 mol) were added. Then added 7 mL of toluene, 2.5 mL of EtOH and 3.5 mL of 2 M K_2CO_3 (aq) solution. Mixture was bubbled with argon for 15 min. Then added $Pd[PPh_3]_4$ (137.6 mg, 0.119 mmol) and the mixture was further bubbled with argon for 15 min. The reaction mixture was stirred at 100 ^oC for 16 h under Ar atmosphere. Reaction was monitored by TLC. After the completion of reaction, the mixture was cooled to rt. Toluene was evaporated under vacuum in rotary evaporator. Crude material was dissolved in 10 mL of chloroform and 10 mL of DI water was added. Organic layer was separated, and aqueous layer was extracted with chloroform, (3 x 10 mL). Combined organic layer was washed with brine solution and dried with Na2SO4 (anhydrous). The mixture was concentrated under reduced pressure using a rotary evaporator. Crude mixture was purified by Column chromatography.

Column chromatography: Crude material was adsorbed over ~ 5 mL of silica. Slurry of silica (50 mL) in pentane was loaded into a column. To the silica packed column, crude material adsorbed over silica was

added. Column was further eluted with 500 mL of 100% pentane. Eluent was collected as 25 mL fraction in a test tube. Following solvent mixtures were eluted.

100 mL of 100 % pentane, Fractions from 10-20 were concentrated to get pure product. (306 mg, yield = 77 %).

Characterization: Yellow solid, Rf: 0.8 in 100% pentane. 1H NMR (400 MHz, $CDCl_3$): δ 8.61 (dt, J = 8.9, 1.0 Hz, 2H), 7.65 (dt, J = 8.8, 1.0 Hz, 2H), 7.58 (dddd, J = 9.3, 8.6, 5.9, 1.0 Hz, 5H), 7.39 (tdd, J = 8.8, 6.3, 1.5 Hz, 4H). 13C NMR (101 MHz, $CDCl_3$): δ 138.52, 137.94, 131.26, 131.16, 130.35, 128.58, 127.98, 127.88, 127.53, 127.08, 125.67, 122.85 . MALDI-HRMS m/z: Mass calculated for [M+] ion for C20H13Br; theoretical = 332.0201, observed = 332.0202, |Δppm| = 0.3 ppm.

3.11. Synthesis of 9-Phenyl-10-(2-phenylethynyl)anthracene

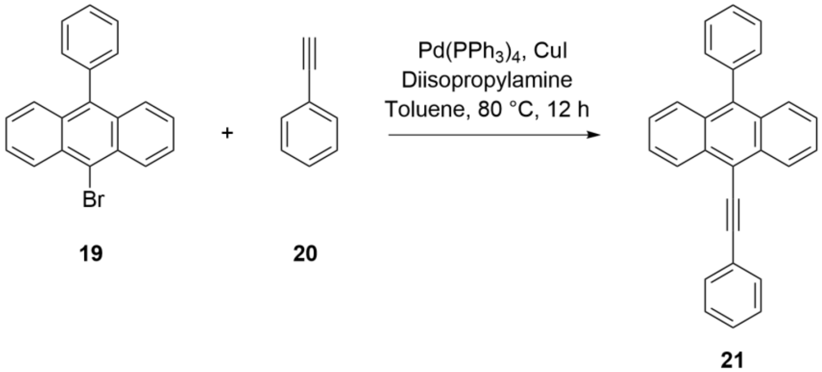

Procedure for this synthesis was adapted from previous report.[16] In a 50 mL oven dried round bottomed flask 9-bromo-10-phenylanthracene (140 mg, 0.42 mmol), CuI (3.2 mg, 0.017 mmol), and $Pd(PPh_3)_4$ (20.8 mg, 0.013 mmol) were taken and flushed with argon gas. Then added 8 mL of toluene and 8 mL of diisopropylamine. The mixture was bubbled with argon gas for 15 min. Then phenyl acetylene (0.14 mL, 1.26 mmol) was added. The mixture was further stirred for 48 h under reflux condition at 100 ^oC. Reaction was monitored by TLC. After the completion of reaction, the mixture was cooled to rt. Toluene was evaporated in the rotary evaporator. The crude material was dissolved in 10 mL of chloroform followed by the addition of DI water (10 mL). Then organic layer was separated, and the aqueous layer was extracted with chloroform (3 x 10 mL). Combined organic layer was washed with brine solution and dried with Na2SO4 (anhydrous). The mixture was concentrated under reduced pressure. Crude mixture was purified by Column chromatography.

Column chromatography: Crude material was adsorbed over ~ 10 mL of silica. Slurry of silica (100 mL) in pentane was loaded into a column. To the silica packed column, crude material adsorbed over silica was added. Column was further eluted with 500 mL of 100% pentane. Eluent was collected as 25 mL fraction in a test tube. Following solvent mixtures were eluted.

600 mL of 100 % pentane, 200 mL of 2 % DCM/pentane, 200 mL of 4 % DCM/pentane. Fractions from 50-61 concentrated. The material obtained was further subjected to recrystallization to isolate pure product.

Recrystallization: The material obtained after column chromatography was dissolved in DCM (3.5 mL) in a glass vial. The solution was layered with methanol (3 mL). The vial was closed with cap and allowed to stand still at rt for 48 h. The crystals formed were isolated and washed with methanol to get pure product. (97 mg yield = 65 %).

Characterization: Yellow solid, Rf: 0.5 in 100% Pentane. 1H NMR (400 MHz, $CDCl_3$): δ 8.73 (dt, *J* = 8.3, 1.0 Hz, 2H), 7.83 – 7.74 (m, 2H), 7.67 (dd, *J* = 8.7, 1.0 Hz, 2H), 7.62 – 7.52 (m, 5H), 7.49 – 7.35 (m, 7H). 13C NMR (101 MHz, $CDCl_3$): δ 138.65, 138.55, 132.41, 131.82, 131.29, 130.08, 128.69, 128.63, 128.53, 127.80,

127.55, 127.04, 126.47, 125.74, 123.83, 117.57, 101.09, 86.68. MALDI-HRMS m/z: Mass calculated for [M+] ion for C28H18; theoretical = 354.1409, observed = 354.14091, |Δppm| = 0.03 ppm.

4. TTA-UC quantum yield data for 4CzBN, Nap/BT system

Table S1: TTA UC QY before and after reabsorption correction for upconversion samples in deaerated toluene.

Entry [a]	Upconversion system	TTA-UC QY (%)	
		With reabsorption correction[c]	No reabsorption correction[c]
1	25 μM 4CzBN + 1mM Nap[d]	12.6±0.3	6.05±0.5
2	25 μM 4CzBN + 1mM Nap (10 mm cuvette)[b]	11.1±0.05	4.92±0.01
3	25 μM 4CzBN + 0.1 mM Nap + 1mM BT[d]	12.2±0.3	7.2±0.3
4	25 μM 4CzBN + 0.1 mM Nap	3.38±0.01	2.0±0.05
5	25 μM 4CzBN + 0.01 mM Nap + 1mM BT	7.8±0.15	5.4±0.1
6	25 μM 4CzBN + 0.01 mM Nap	0.2±0.02	0.15±0.02
7	25 μM 4CzBN + 1 mM BT	1.5±0.05	1.0±0.1
8	25 μM 4CzBN + 10 mM BT	1.45±0.01	0.78±0.01
9	25 μM 4CzBN + 0.1 mM Nap + 10mM BT	12.5±0.55	6.7±0.3
10	25 μM 4CzBN + 0.1 mM Nap + 1 mM Ph	5.1±0.3	3.0±0.2

[a]Entry 1, 3-10, TTA-UC QY was measured in 4 mm × 10 mm cuvette, where sample was excited across 10 mm path of cuvette, with emitted light detected in 90° angle, passings through the 4 mm path to minimize reabsorption of emitted light. [b]Entry 2, TTA-UC QY was measured in 10 mm × 10 mm cuvette to compare with that of 4 mm × 10 mm cuvette for one sample (4CzBN + 1mM Nap). [c]Averaged over two trials except for entry 1 and 3. [d]Averaged over four trials with standard deviation as error. Coumarin 153 in aerated ethanol (Φ_F = 0.53) was used as a standard to determine UCQY.[7] The reported QY values are with respect to the maximum value of 50%.

5. Plot of integrated area of upconverted emission intensity vs laser energy for 4CzBN, Nap/BT system

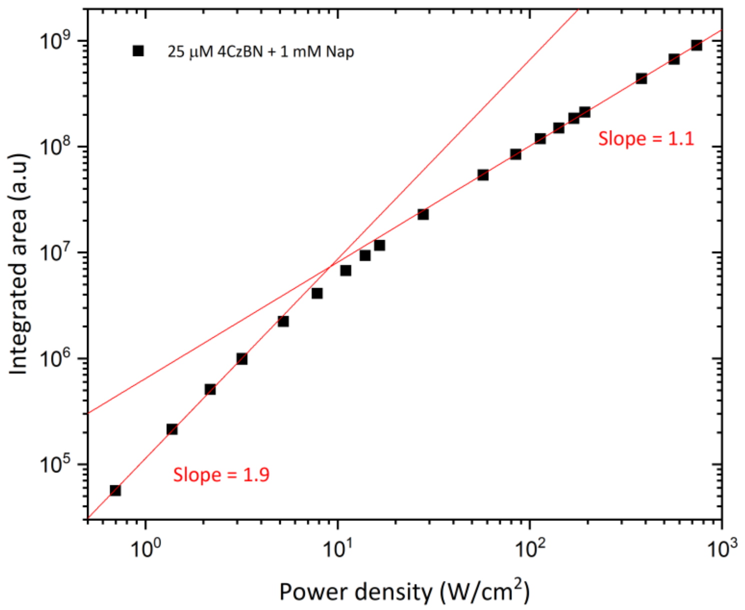

Figure S1: Plot of integrated area vs laser power density of upconverted emission intensity for 25 μM 4CzBN + 1 mM Nap.

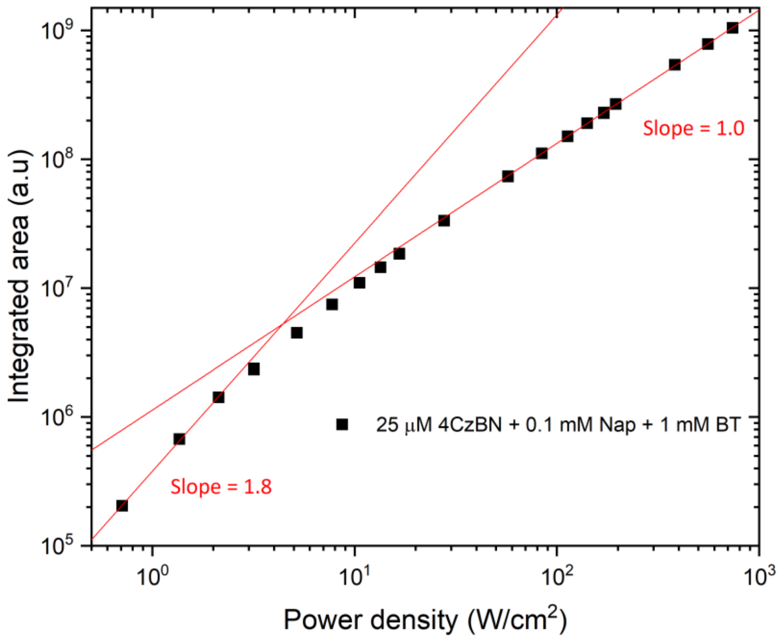

Figure S2: Plot of integrated area of upconverted emission intensity vs laser power density for 25 μM 4CzBN + 0.1 mM Nap + 1 mM BT.

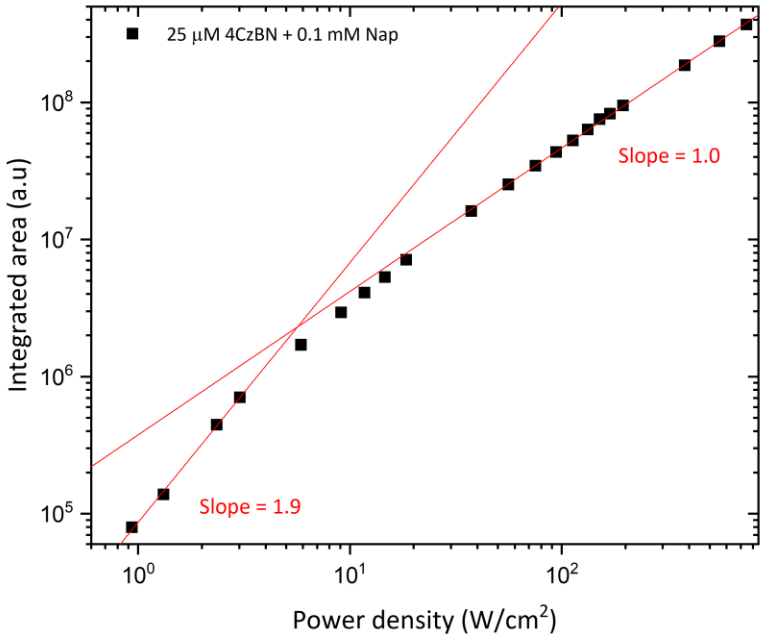

Figure S3: Plot of integrated area of upconverted emission intensity vs laser power density for 25 μM 4CzBN + 0.1 mM Nap.

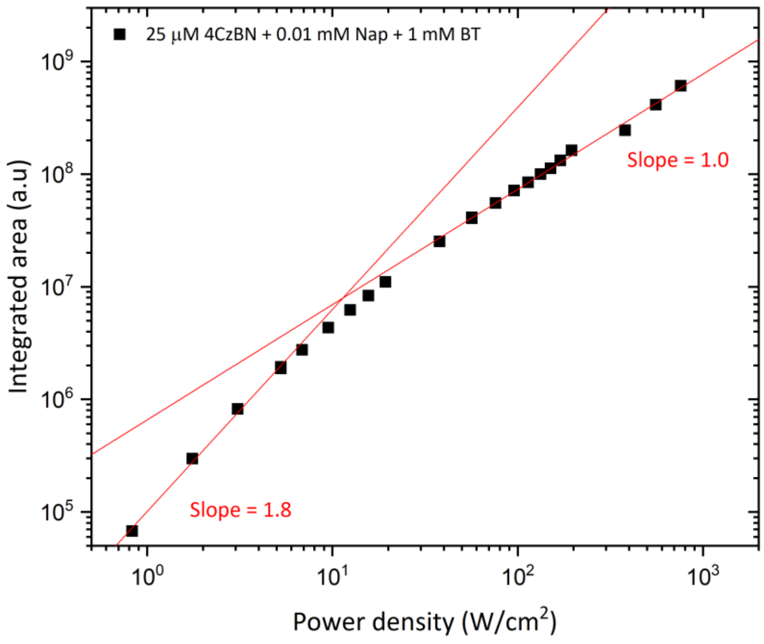

Figure S4: Plot of integrated area of upconverted emission intensity vs laser power density for 25 μM 4CzBN + 0.01 mM Nap + 1 mM BT.

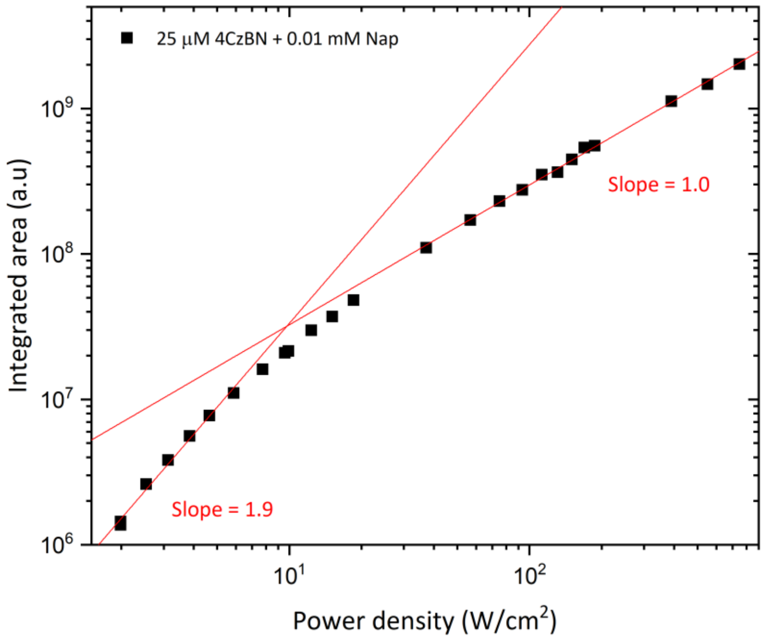

Figure S5: Plot of integrated area of upconverted emission intensity vs laser power density for 25 μM 4CzBN + 0.01 mM Nap.

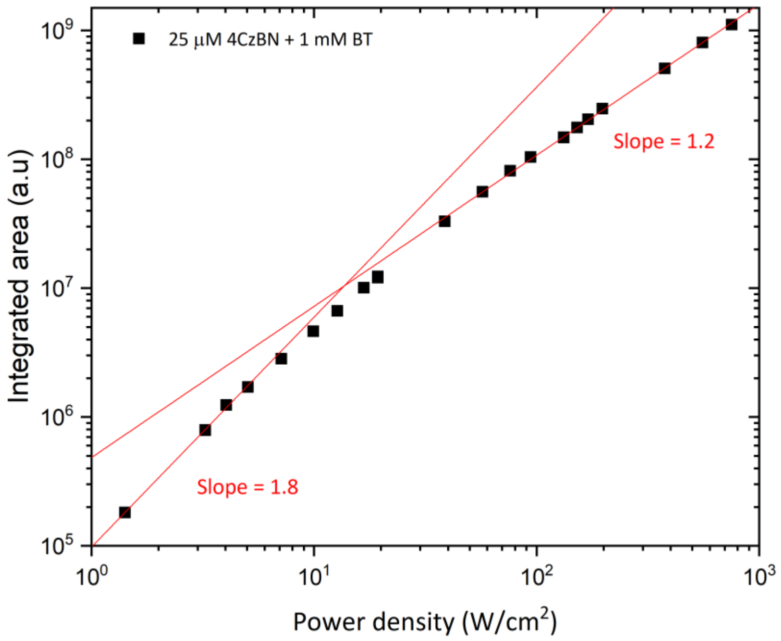

Figure S6: Plot of integrated area of upconverted emission intensity vs laser power density for 25 μM 4CzBN + 1 mM BT.

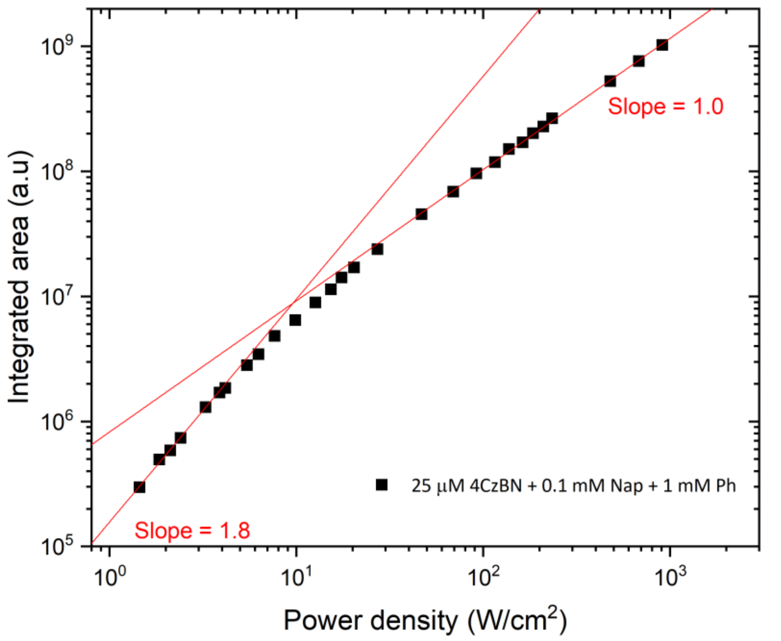

Figure S7: Plot of integrated area of upconverted emission intensity vs laser power density for 25 μM 4CzBN + 0.1 mM Nap + 1 mM Ph.

Table S2: I^{th} value and slope in the plot of integrated area of emission intensity vs laser energy in deaerated toluene for 4CzBN, Nap/BT system.[a]

Entry	Upconversion system	Slope in low intensity region	Slope in high intensity region	I_{th} value (W/cm^2)
1	25 μM 4CzBN + 1mM Nap	1.9	1.1	9.2
2	25 μM 4CzBN + 0.1 mM Nap + 1mM BT	1.8	1.0	4.4
3	25 μM 4CzBN + 0.1 mM Nap	1.9	1.1	5.7
4	25 μM 4CzBN + 0.01 mM Nap + 1mM BT	1.8	1.0	11.4
5	25 μM 4CzBN + 0.01 mM Nap	1.9	1.0	9.8
6	25 μM 4CzBN + 1 mM BT	1.8	1.2	13.7
7	25 μM 4CzBN + 0.1 mM Nap + 1 mM Ph	1.8	1.0	9.8

[a]TTA-UC emission was measured in 10 mm × 10 mm cuvette. Laser energy was varied from high to low energy for each measurement. The straight line obtained after fitting the data in the slope 1 and slope 2 region of integrated area vs power density plot was used to determine I_{th} value. Intersection point was determined after solving the equation for straight line.

6. UV-vis absorption spectra of upconversion sample 4CzBN, Nap/BT system

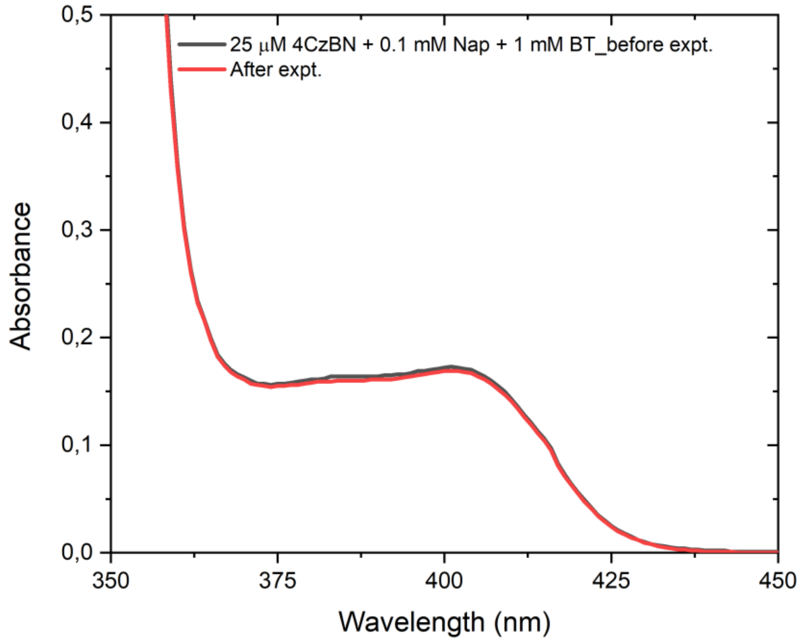

Figure S8: UV-vis absorption spectra of 25 μM 4CzBN + 0.1 mM Nap + 1 mM BT before and after laser excitation in the experiment for the determination of I_{th} value.

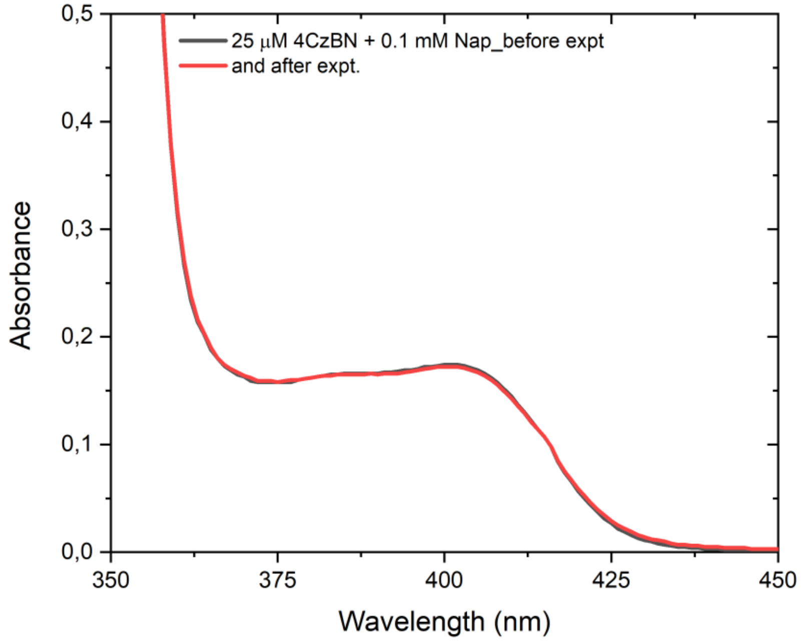

Figure S9: UV-vis absorption spectra of 25 μM 4CzBN + 0.1 mM Nap before and after laser excitation in the experiment for the determination of I_{th} value.

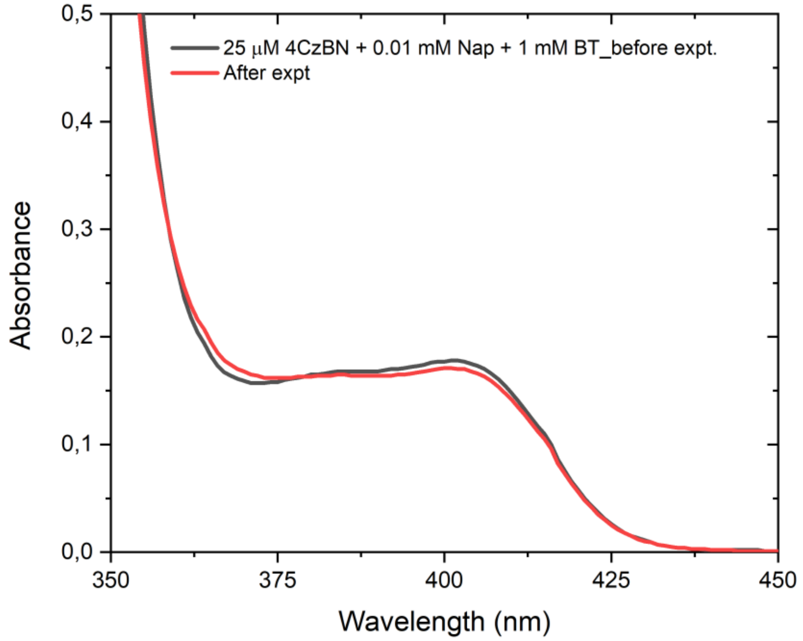

Figure S10: UV-vis absorption spectra of 25 μM 4CzBN + 0.01 mM Nap + 1mM BT before and after laser excitation in the experiment for the determination of I_{th} value.

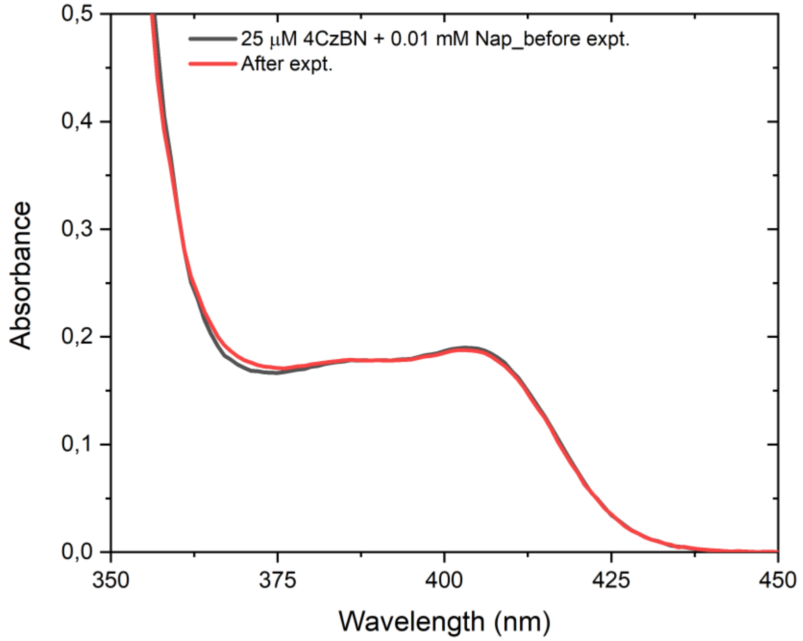

Figure S11: UV-vis absorption spectra of 25 μM 4CzBN + 0.01 mM Nap before and after laser excitation in the experiment for the determination of I_{th} value.

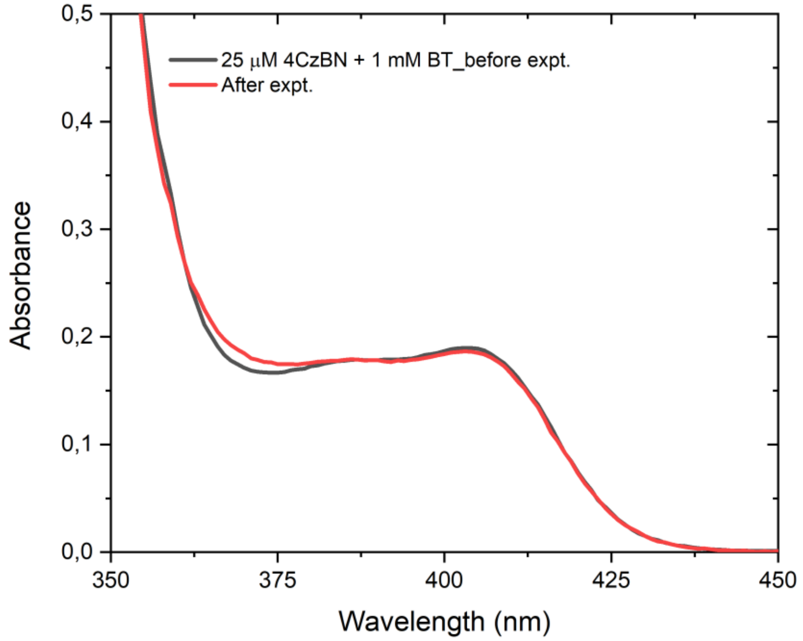

Figure S12: UV-vis absorption spectra of 25 μM 4CzBN + 1 mM BT before and after laser excitation in the experiment for the determination of I_{th} value.

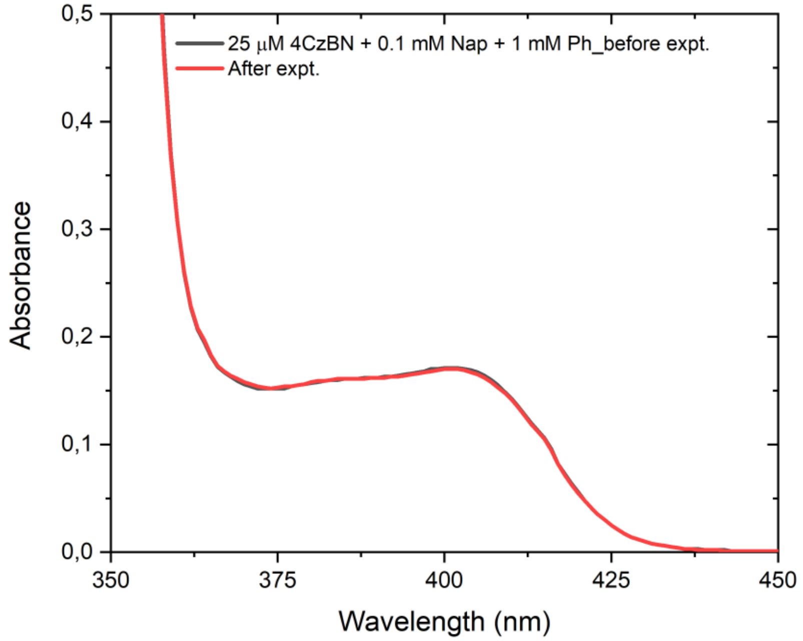

Figure S13: UV-vis absorption spectra of 25 μM 4CzBN + 0.1 mM Nap + 1 mM Ph before and after laser excitation in the experiment for the determination of I_{th} value.

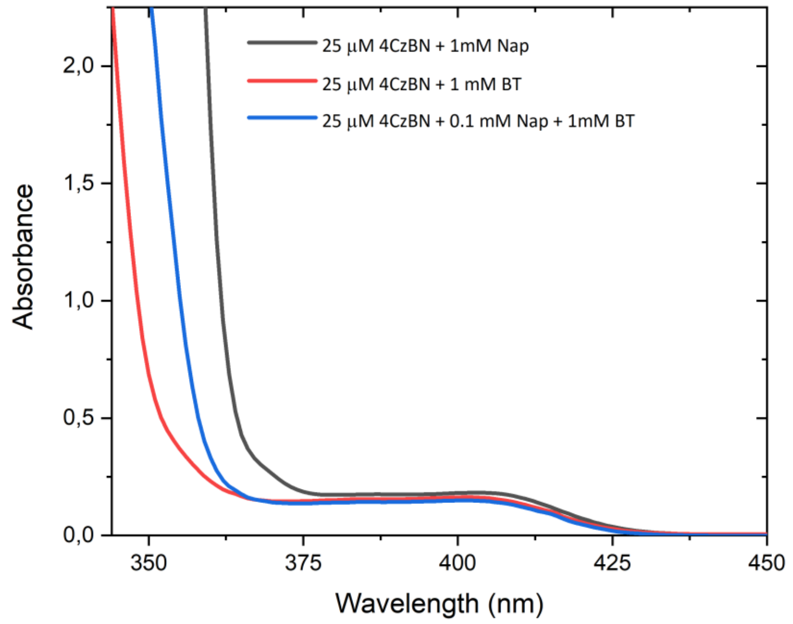

Figure S14: Comparison of UV-vis absorption spectra of upconversion sample 25 μM 4CzBN + 0.1 mM Nap + 1mM BT with that of 25 μM 4CzBN + 1 mM Nap and 25 μM 4CzBN + 1 mM BT.

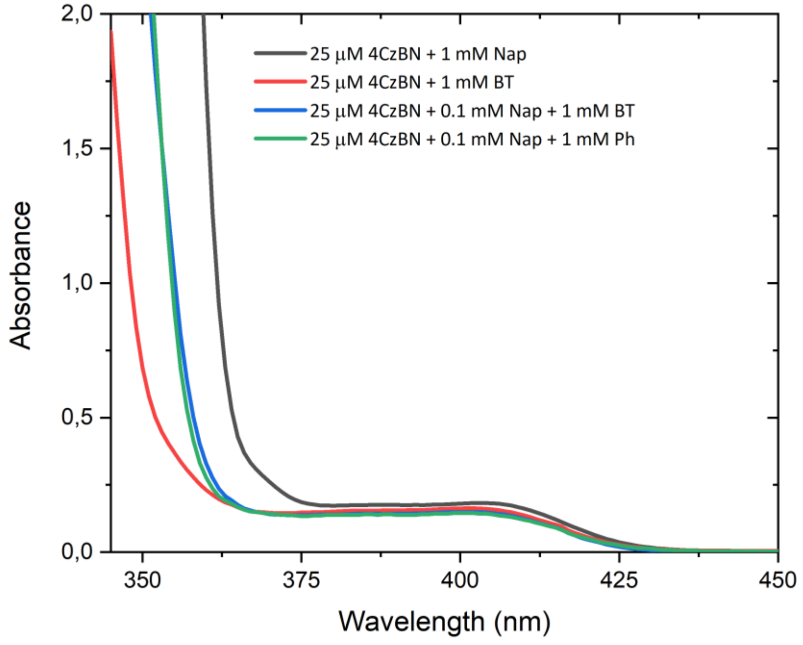

Figure S15: Comparison of UV-vis absorption spectra of upconversion sample 25 μM 4CzBN + 0.1 mM Nap + 1 mM Ph with that of 25 μM 4CzBN + 1 mM Nap, 25 μM 4CzBN + 0.1 mM Nap + 1 mM BT and 25 μM 4CzBN + 1 mM BT.

7. TTA-UC quantum yield data for PtOEP, PPE-A/DPA system

Table S3: TTA UC QY before reabsorption correction for upconversion samples in deaerated toluene.

Entry [a]	Upconversion system	TTA-UC QY (%)[c]
		No reabsorption correction[b]
1	0.01 mM PPE-A + 0.1 mM DPA + 15 μM PtOEP	13.1±0.6
2	0.01 mM PPE-A + 15 μM PtOEP	3.75±0.05
3	0.001 mM PPE-A + 0.1 mM DPA + 15 μM PtOEP	14.35±0.05
4	0.001 mM PPE-A + 15 μM PtOEP	0.19±0.03
5	0.01 mM PPE-A + 1 mM DPA + 15 μM PtOEP	15.3±0.2
6	0. 1 mM DPA + 15 μM PtOEP	13.15±0.35

[a]Entry 1-6, TTA-UC QY was measured in 4 mm × 10 mm cuvette, where sample was excited across 10 mm path of cuvette, with emitted light detected in 90° angle, passings through the 4 mm path to minimize reabsorption of emitted light. [b]Averaged over two trials. Rhodamine 6G in aerated ethanol (Φ_F = 0.95)[8] was used as a standard to determine UCQY. The reported QY values are with respect to the maximum value of 50%. All measurements are made at high excitation intensity of 35 W/cm^2.

8. Plot of integrated area of upconverted emission intensity vs laser energy for PtOEP, PPE-A/DPA system

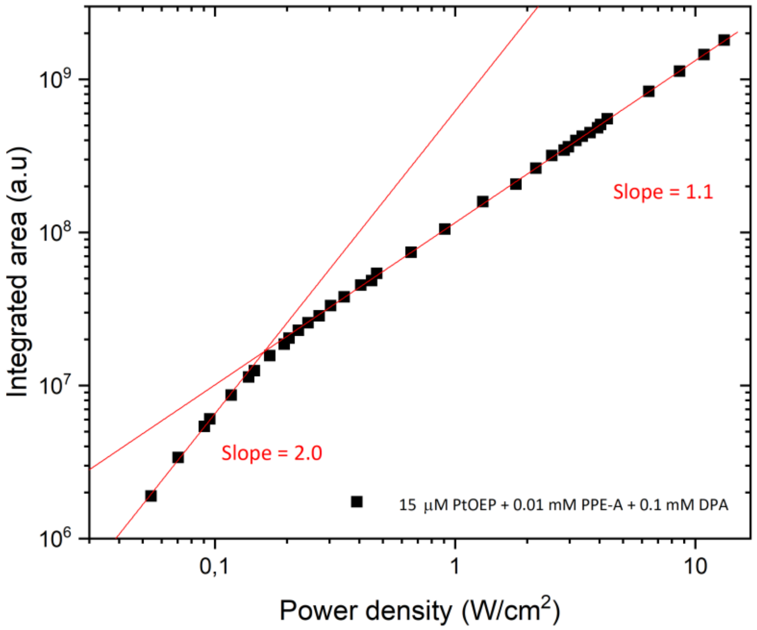

Figure S16: Plot of integrated area of upconverted emission intensity vs laser power density for 15 μM PtOEP + 0.01 mM PPE-A + 0.1 mM DPA.

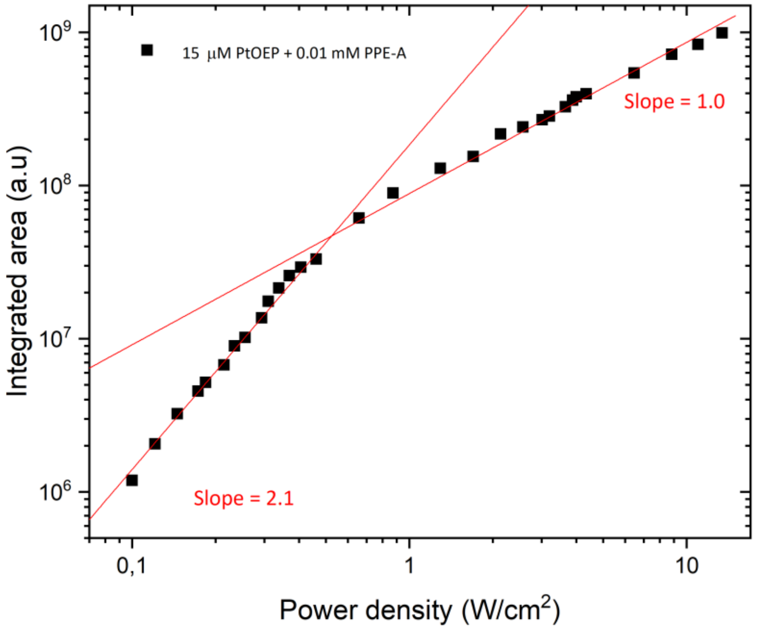

Figure S17: Plot of integrated area of upconverted emission intensity vs laser power density for 15 μM PtOEP + 0.01 mM PPE-A.

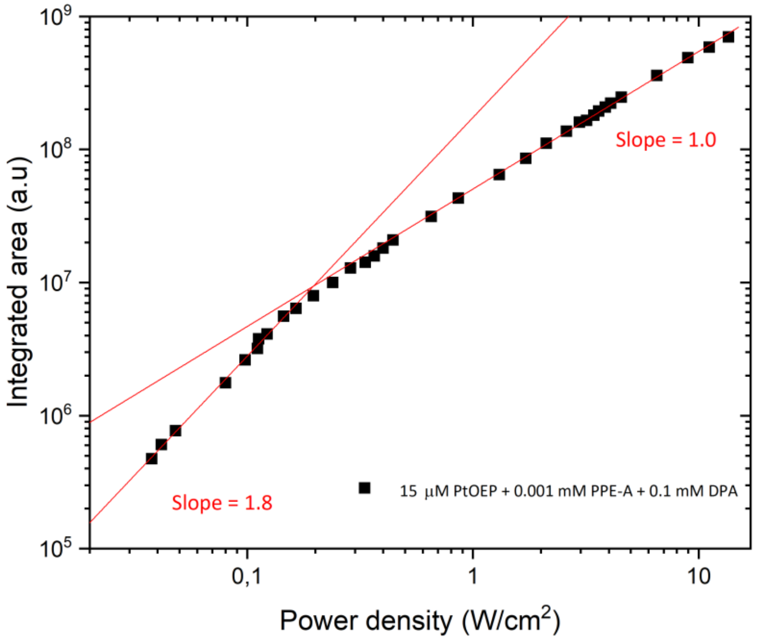

Figure S18: Plot of integrated area of upconverted emission intensity vs laser power density for 15 μM PtOEP + 0.001 mM PPE-A + 0.1 mM DPA.

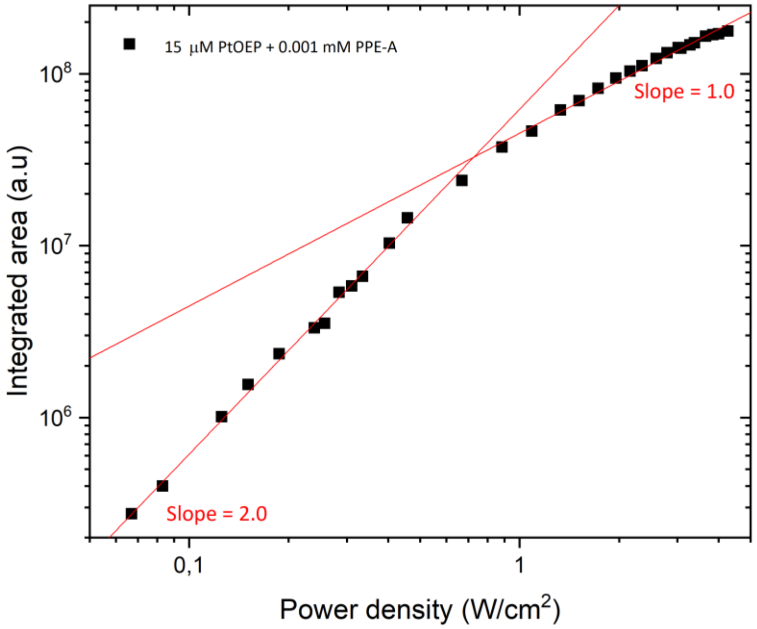

Figure S19: Plot of integrated area of upconverted emission intensity vs laser power density for 15 μM PtOEP + 0.001 mM PPE-A.

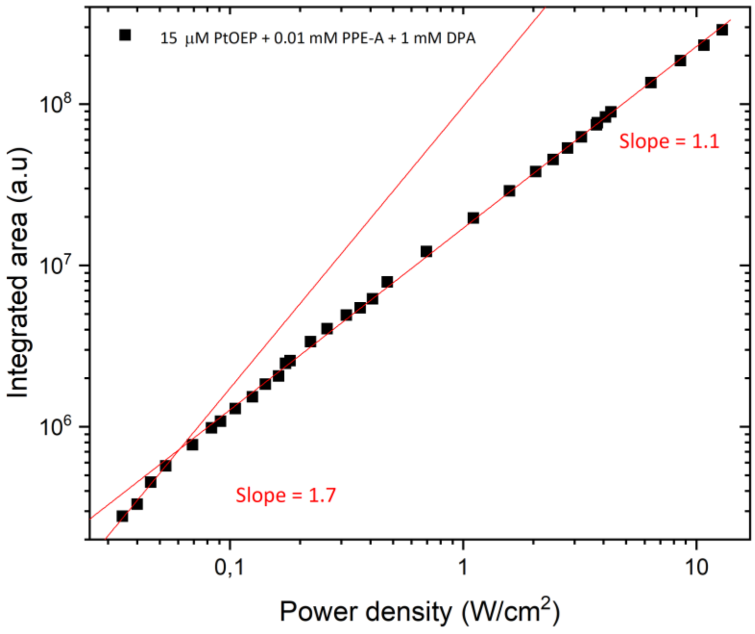

Figure S20: Plot of integrated area of upconverted emission intensity vs laser power density for 15 μM PtOEP + 0.01 mM PPE-A + 1 mM DPA.

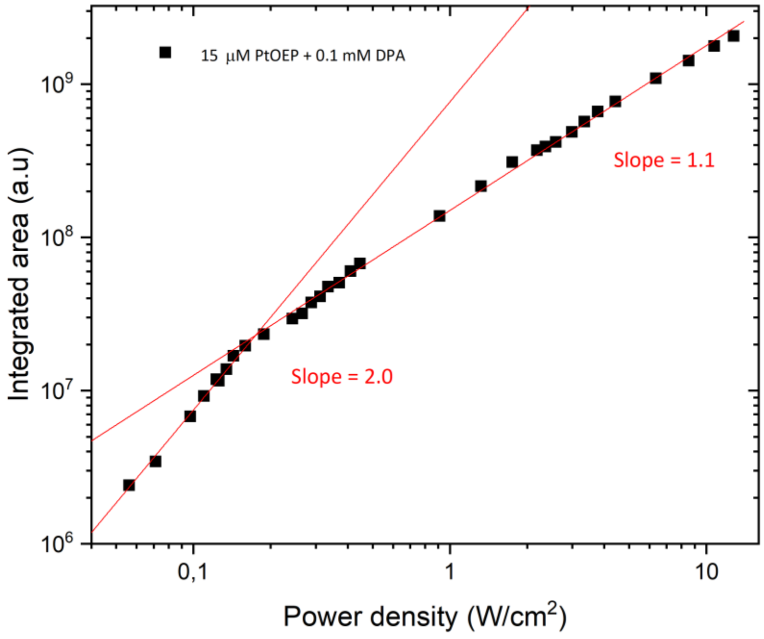

Figure S21: Plot of integrated area of upconverted emission intensity vs laser power density for 15 μM PtOEP + 0.1 mM DPA

Table S4: I^{th} value and slope in the plot of integrated area of emission intensity vs laser energy in deaerated toluene for PtOEP, PPE-A/DPA system.[a]

Entry	Upconversion system	Slope in low intensity region	Slope in high intensity region	I_{th} value (W/cm^2)
1	0.01 mM PPE-A + 0.1 mM DPA + 15 μM PtOEP	2.0	1.1	0.16
2	0.01 mM PPE-A + No mediator+ 15 μM PtOEP	2.1	1.0	0.52
3	0.001 mM PPE-A + 0.1 mM DPA + 15 μM PtOEP	1.8	1.0	0.20
4	0.001 mM PPE-A + No mediator+ 15 μM PtOEP	2.0	1.0	0.73
5	0.01 mM PPE-A + 1 mM DPA + 15 μM PtOEP	1.7	1.1	0.06
6	0. 1 mM DPA + 15 μM PtOEP 15 μM	2.0	1.0	0.18

[a]TTA-UC emission was measured in 10 mm × 10 mm cuvette. Laser energy was varied from high to low energy for each measurement. The straight line obtained after fitting the data in the slope 1 and slope 2 region of integrated area vs power density plot was used to determine I_{th} value. Intersection point was determined after solving the equation for straight line.

9. UV-vis absorption spectra of upconversion sample PtOEP, PPE-A/DPA system

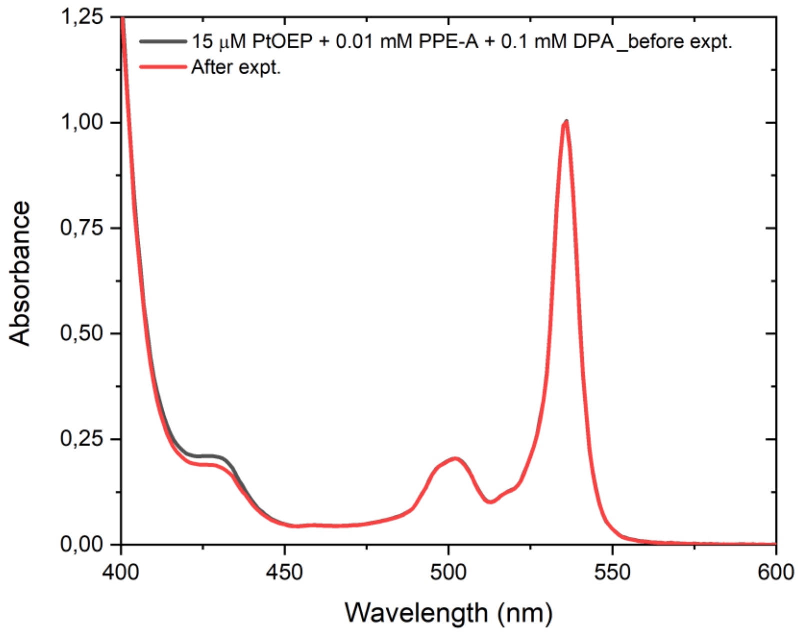

Figure S22: UV-vis absorption spectra of 15 μM PtOEP + 0.01 mM PPE-A + 0.1 mM DPA before and after laser excitation in the experiment for the determination of I_{th} value.

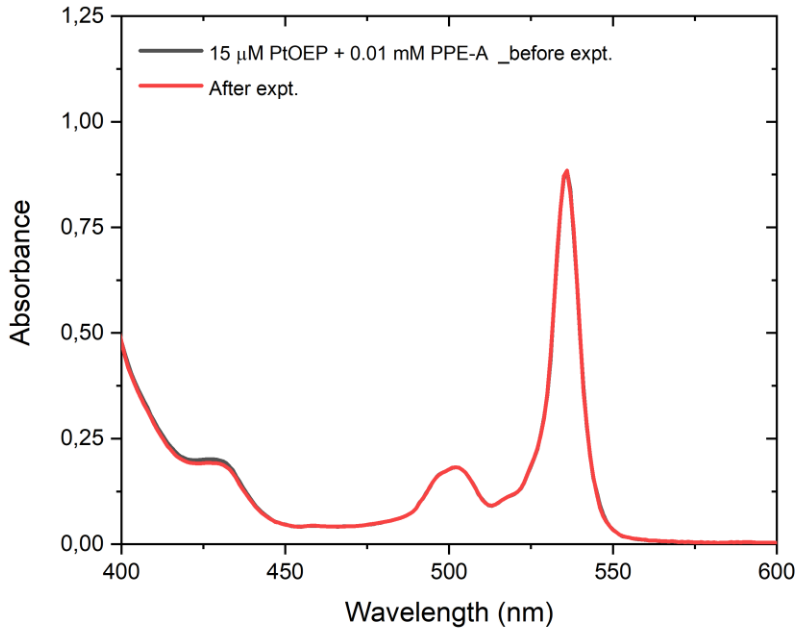

Figure S23: UV-vis absorption spectra of 15 μM PtOEP + 0.01 mM PPE-A before and after laser excitation in the experiment for the determination of I_{th} value.

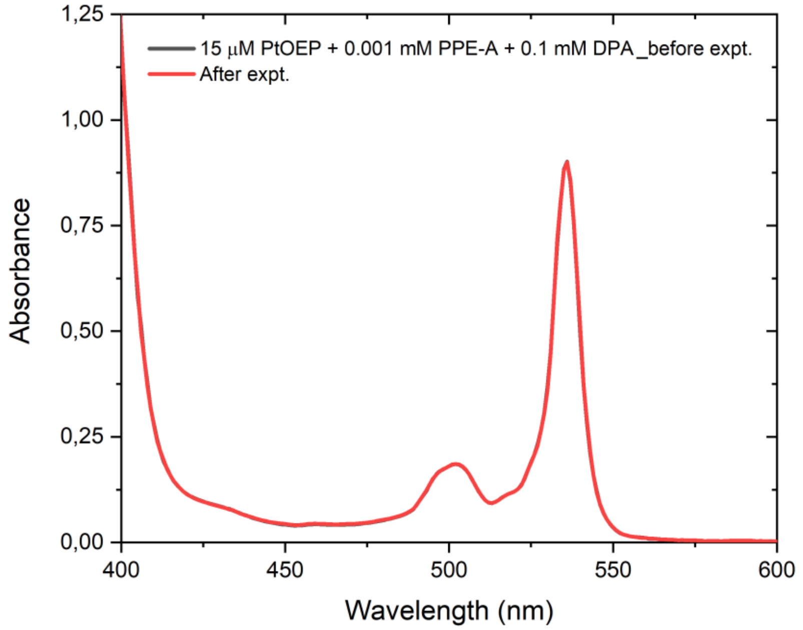

Figure S24: UV-vis absorption spectra of 15 μM PtOEP + 0.001 mM PPE-A + 0.1 mM DPA before and after laser excitation in the experiment for the determination of I_{th} value.

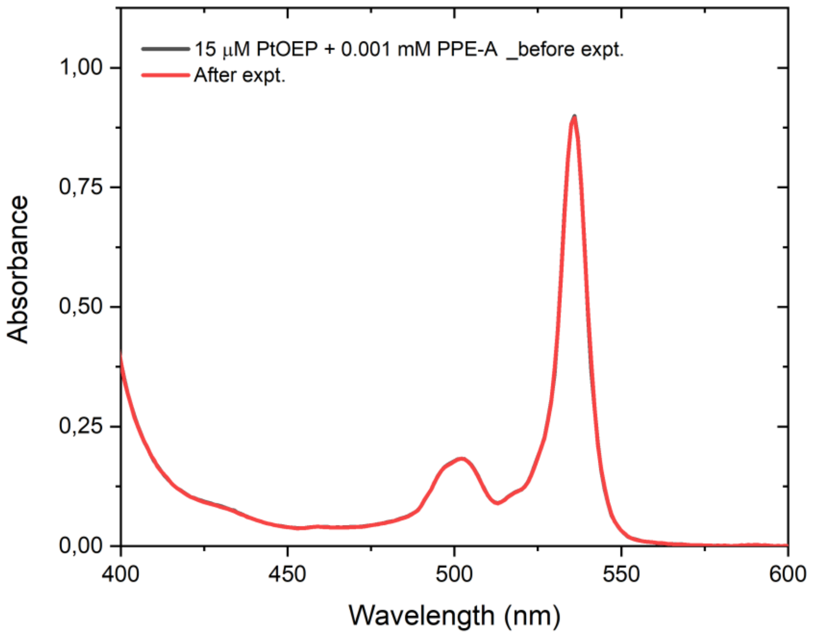

Figure S25: UV-vis absorption spectra of 15 μM PtOEP + 0.001 mM PPE-A before and after laser excitation in the experiment for the determination of I_{th} value.

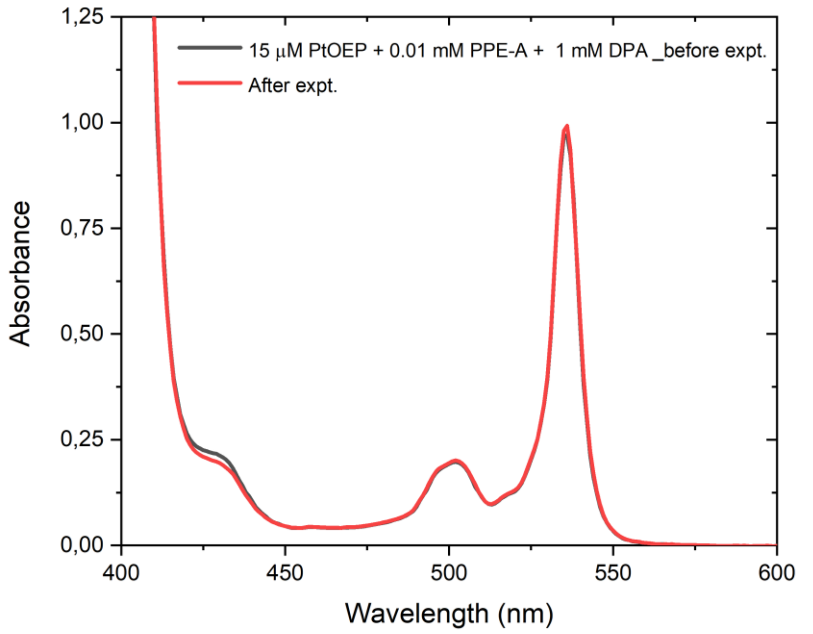

Figure S26: UV-vis absorption spectra of 15 μM PtOEP + 0.01 mM PPE-A + 1 mM DPA before and after laser excitation in the experiment for the determination of I_{th} value.

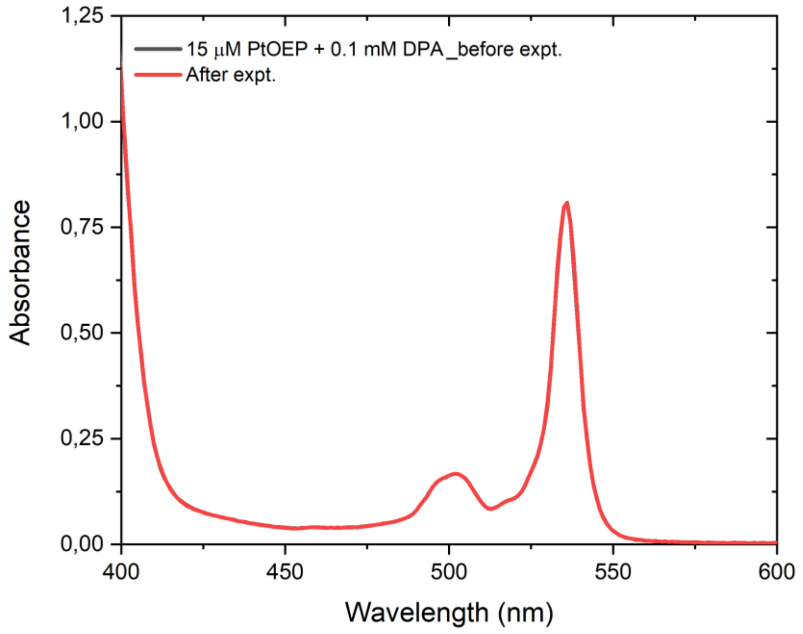

Figure S27: UV-vis absorption spectra of 15 μM PtOEP + 0.1 mM DPA before and after laser excitation in the experiment for the determination of I_{th} value.

10. Determination of emission quantum yield of BT

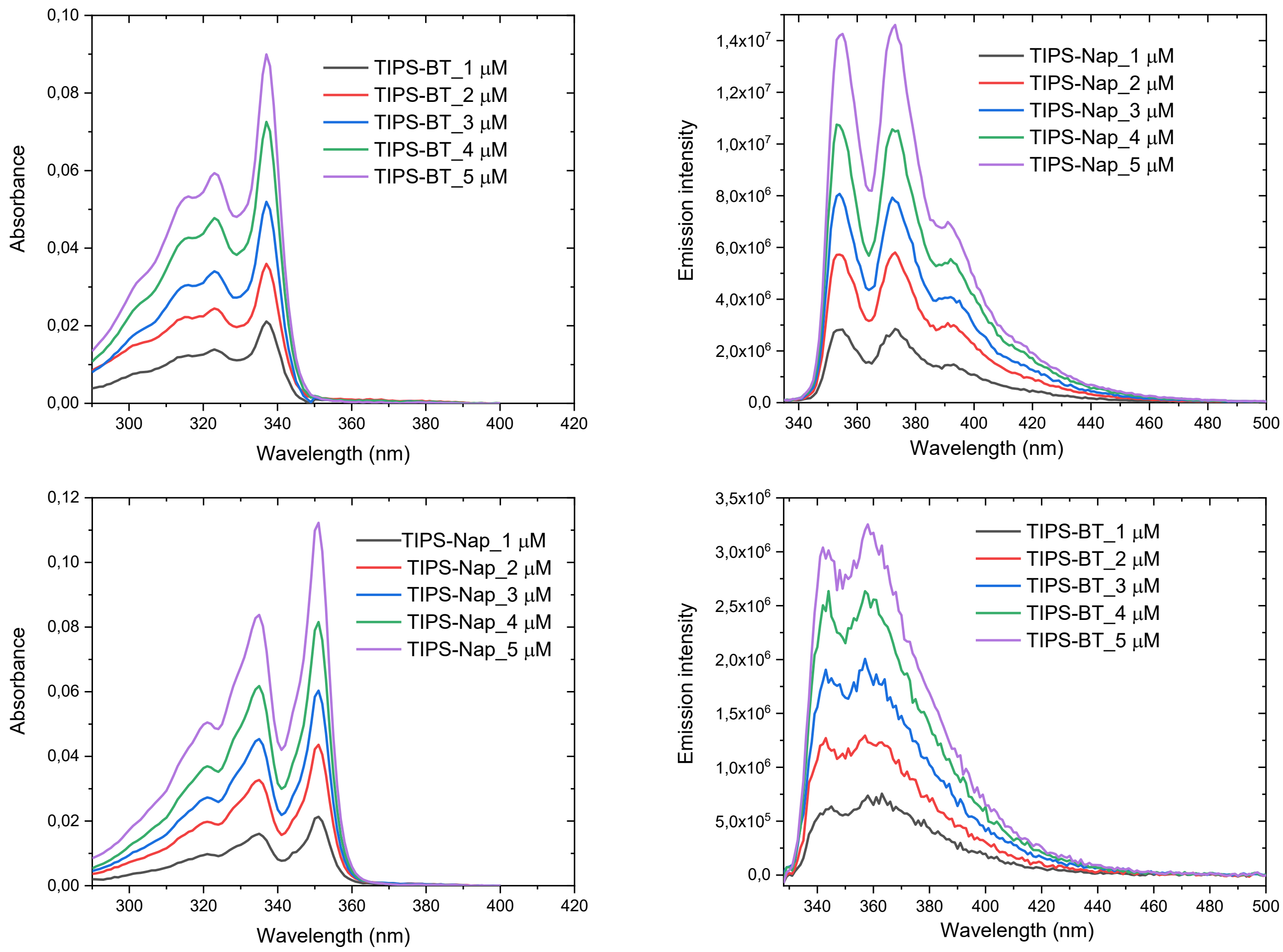

Figure S28: UV-vis absorption (top left and bottom left) and emission (top right and bottom right) spectra of dilute solution of Nap and BT in toluene. Concentration ranges from 1-5 μM.

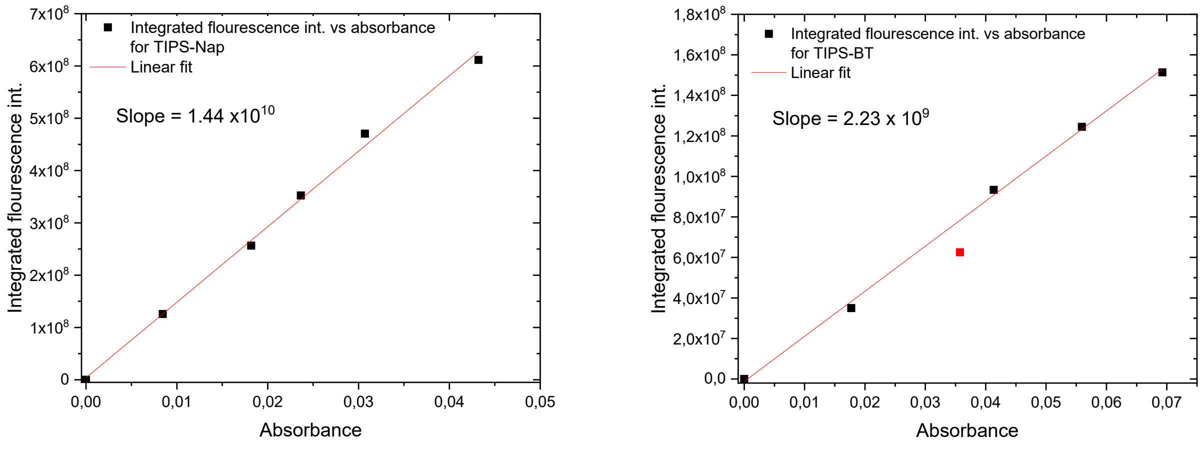

Figure S29: Plot of integrated fluorescence intensity vs absorbance for Nap and BT.

Fluorescence emission quantum yield of BT was determined using Nap as reference in degassed toluene. The fluoresce quantum yield of Nap = 0.77 was adopted from the previous report.[7] In the Equation 2 below to determine the fluorescence emission quantum yield; Φ, A, F and η refers to the fluorescence emission quantum yield, absorbance at the excitation wavelength, integrated fluorescence intensity and the refractive index of the solvent respectively.[5,6] To minimize the error associated with the fluorescence emission quantum yield determination, multiple trials were performed in the low concertation regime (with concentration ranging from 1-5 μM) as depicted in Figure 28 which consists of UV-vis absorbance and emission spectra for each sample. For emission spectra same excitation and emission bandwidth as well as same excitation wavelength was maintained for the reference compound and sample. Plot of integrated emission intensity vs absorbance showed linear relationship as in Figure 19. Further, parameters in Equation 2 can be replaced with the slope of these plots as in Equation 3 to determine fluorescence quantum yield. From this experiment, the fluorescence emission quantum yield of BT was determined to be 11.9%.

$$\Phi_{BT} = \Phi_{Nap}\left[\frac{A_{Nap}}{A_{BT}} \times \frac{F_{BT}}{F_{Nap}} \times \frac{\eta_{BT}^2}{\eta_{Nap}^2}\right] \ldots\ldots\ldots\ldots (2)$$

Or

$$\Phi_{BT} = \Phi_{Nap}[\frac{Slope_{BT}}{Slope_{Nap}} \times \frac{\eta_{Nap}^2}{\eta_{BT}^2}] \ldots\ldots\ldots\ldots (3)$$

11.Stern-Volmer plot

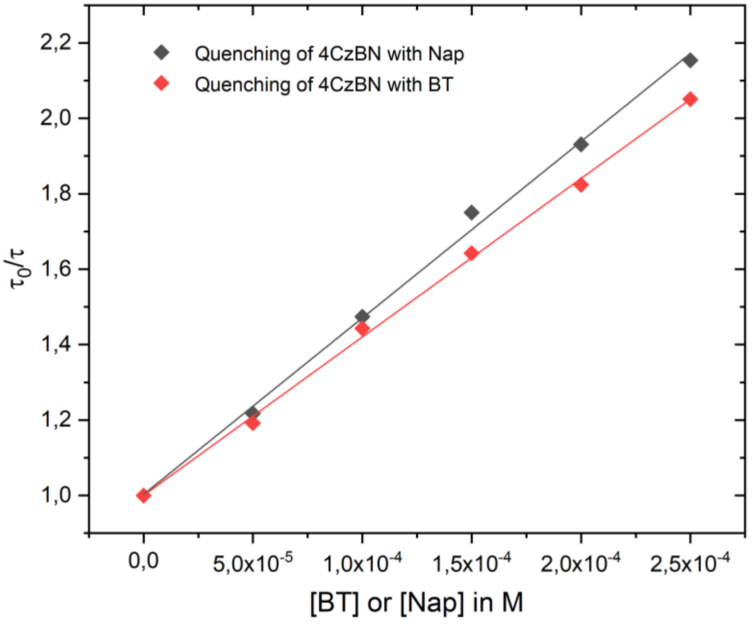

Figure S30: Stern-Volmer plot for the quenching of 4CzBN delayed florescence lifetime with BT and Nap.

For Stern-Volmer plot, quenching of delayed fluorescence component of 4CzBN (since it exhibits TADF behaviour) was considered as reported elsewhere.[7] Quenching experiment was performed in degassed toluene with 25 μM solution of 4CzBN. The quencher concentration ranging from 0.05 mM to 0.25 mM

was added. Rate constant for triplet energy transfer k_{TET} for BT was found to be $0.75 \times 10^9\ M^{-1}s^{-1}$ slightly lower than that of Nap which was $0.84 \times 10^9\ M^{-1}s^{-1}$. Under our experimental condition, lifetime of delayed fluorescence of 4CzBN was found to be 5.6 μs.

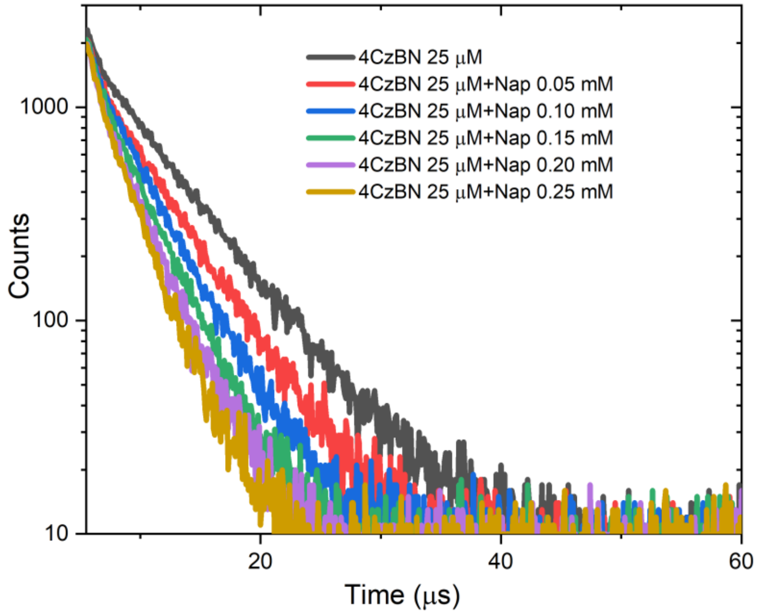

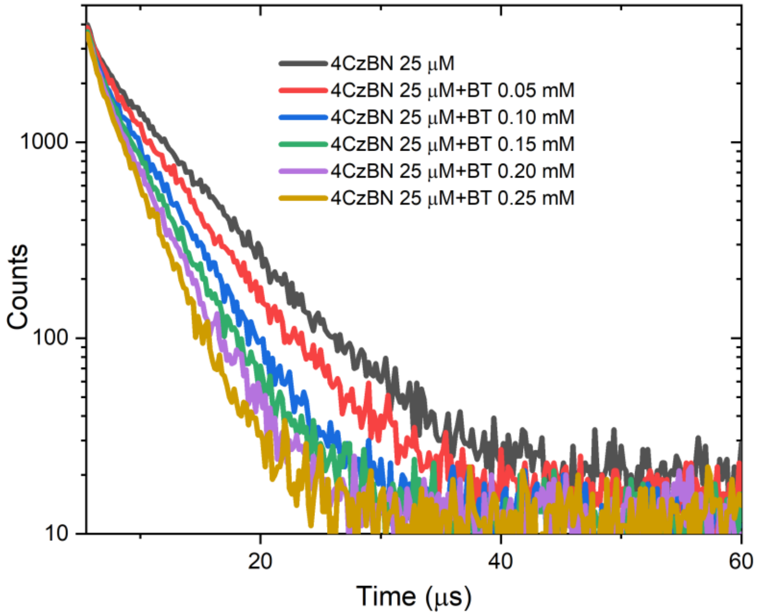

Figure S31: Decay trace of 4CzBN emission upon addition of Nap and BT. Excitation source = MCS laser diode. λ_{ex} = 375 nm and emission monitored at 500 nm. Em BW = 10 nm.

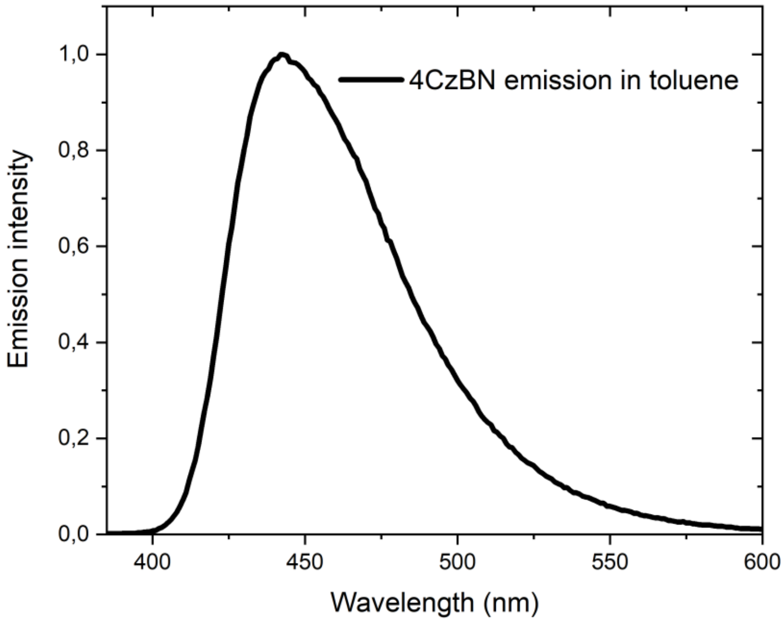

Figure S32: 4CzBN emission spectra in toluene (50 μM). Excitation source = Xe lamp. λ_{ex} = 372 nm, Ex BW= 3 nm, Em BW = 5 nm.

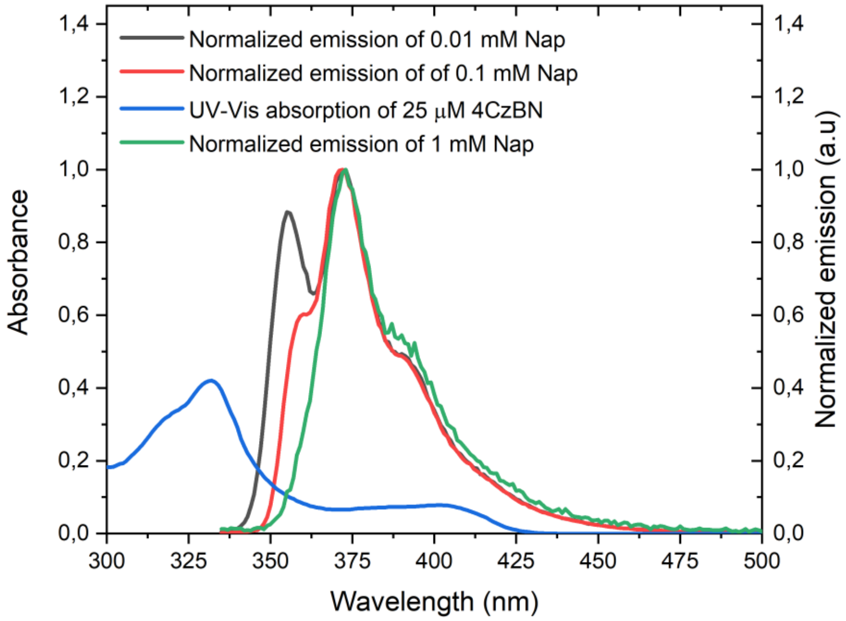

Figure S33: UV-vis absorption spectra of 25 μM 4CzBN and normalized emission spectra of Nap (1 mM, 0.1 mM and 0.01 mM) in toluene.

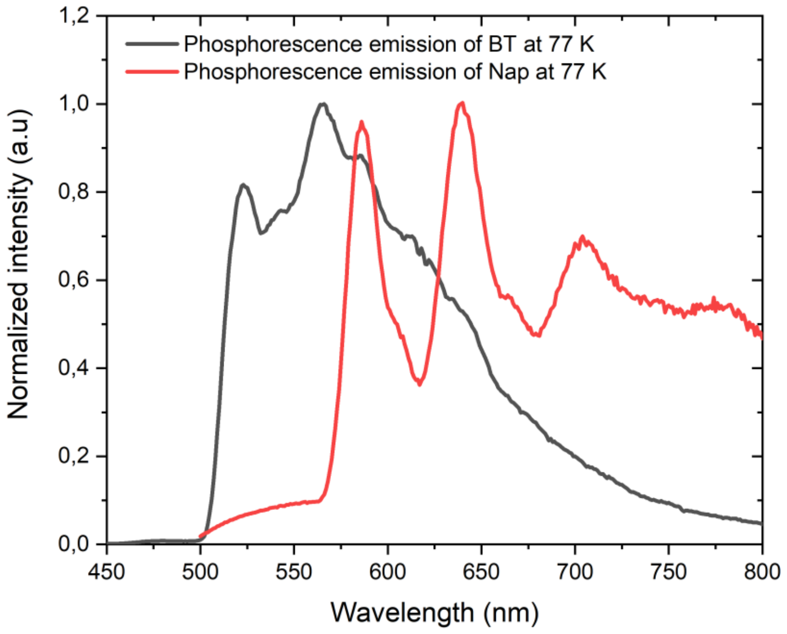

Figure S34: Phosphorescence spectra of Nap and BT in 2-methyl tetrahydrofuran at 77K in quartz tube. Sample was excited at 330 nm with 395 nm long pass filter placed across emission path. Data was collected after 0.05 ms of excitation pulse. Time between the excitation pulse was 61 ms.

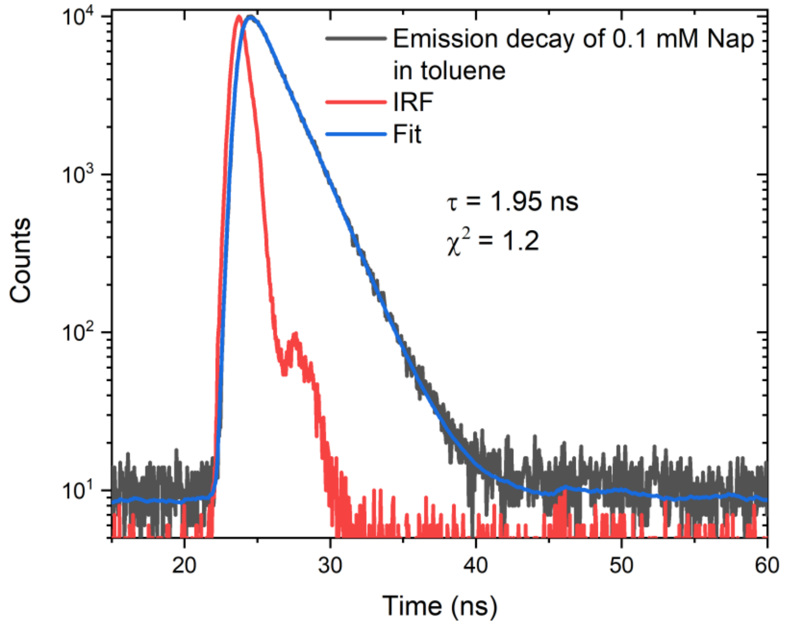

Figure S35: Decay trace of Nap emission. Excitation source = laser diode. λ_{ex} = 340 nm, repetition rate = 10 MHz. Emission monitored at 373 nm. Em BW = 1 nm.

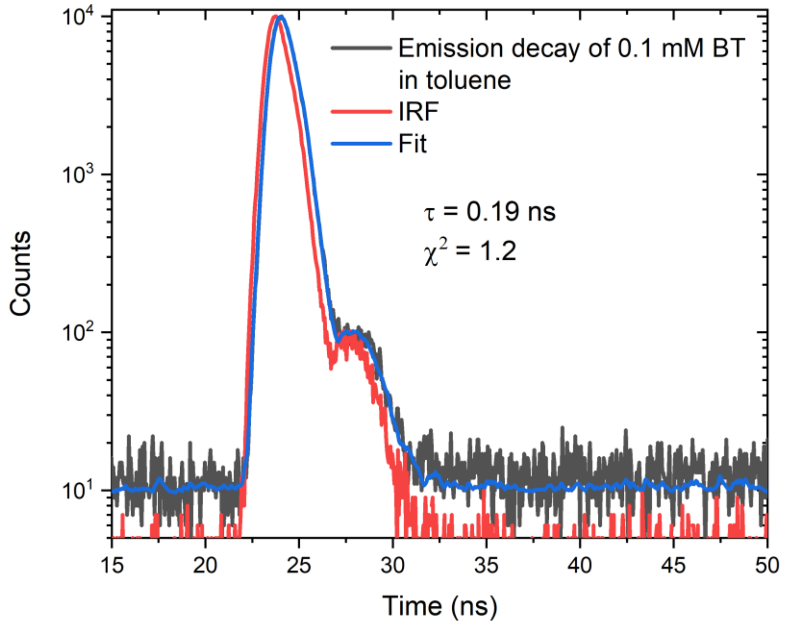

Figure S36: Decay trace of BT emission. Excitation source = laser diode. λ_{ex} = 340 nm, repetition rate = 10 MHz. Emission monitored at 400 nm. Em BW = 3 nm.

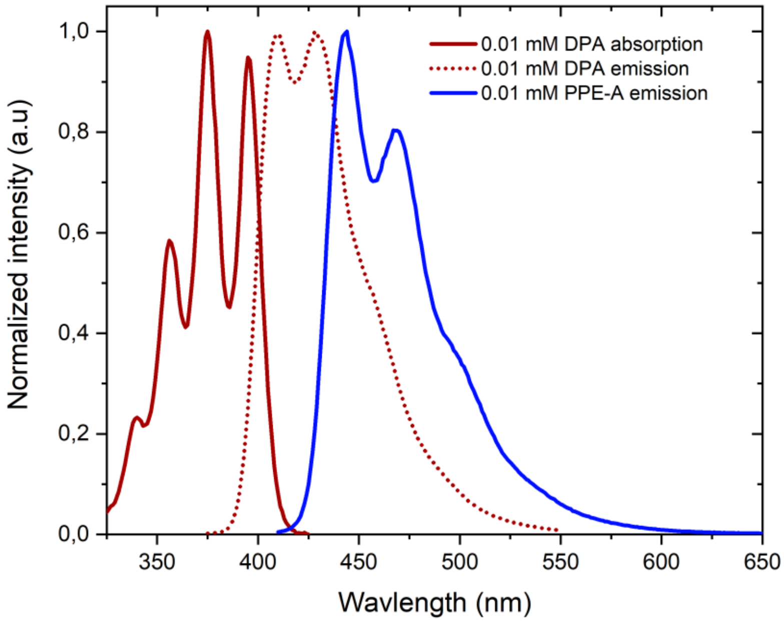

Figure S37: UV-vis absorption spectra of DPA (0.01 mM) and emission spectra of DPA (0.01 mM), PPE-A (0.01 mM).

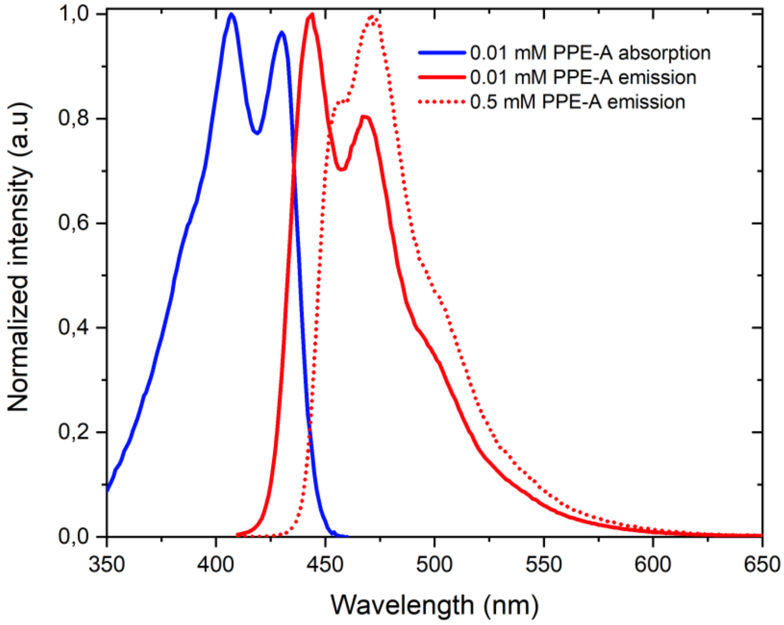

Figure S38: Comparison of UV-vis absorption spectra of PPE-A (0.01 mM) and emission spectra of PPE-A (0.01 mM and 0.05 mM) to analyze the effect of reabsorption of emitted light at higher concentration of PPE-A (0.5 mM).

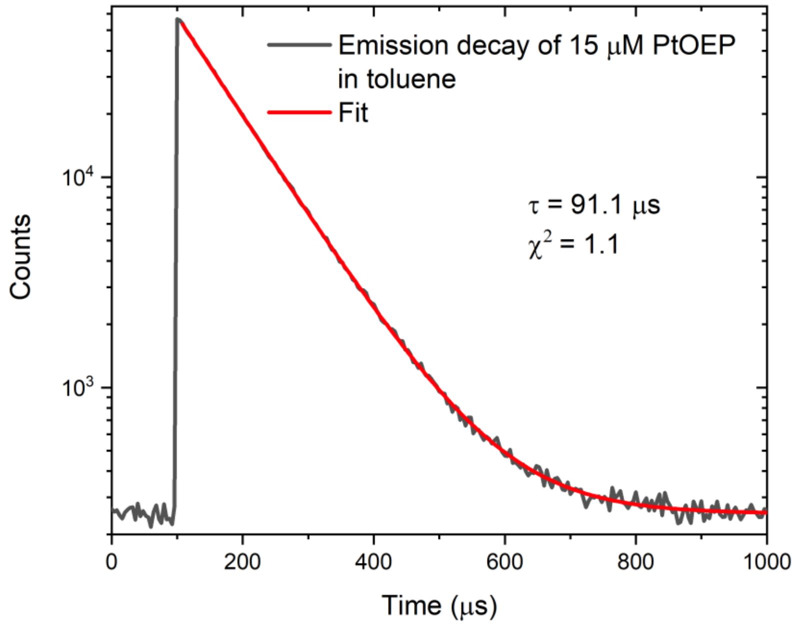

Figure S39: Decay trace of PtOEP emission. Excitation source = MCS laser diode. λ_{ex} = 375 nm, frequency = 500 Hz, pulse period = 2 ms. Emission monitored at 645 nm. Em BW = 5 nm.

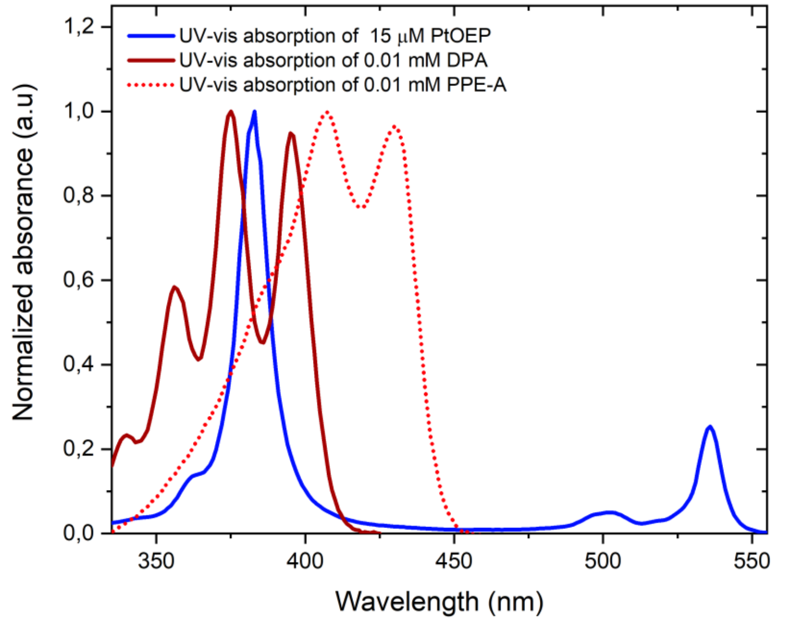

Figure S40: Comparison of UV-vis absorption spectra of DPA, PPE-A with that of PtOEP.

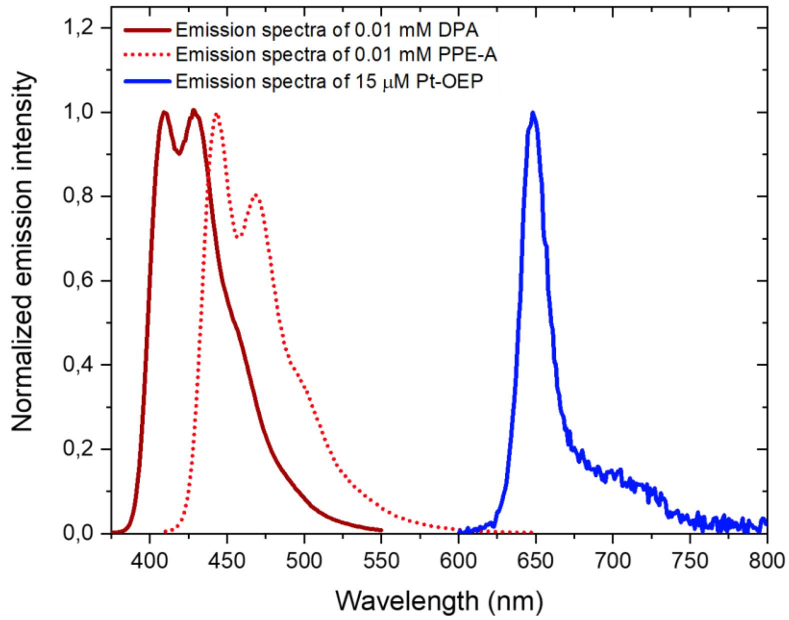

Figure S41: Comparison of emission spectra of DPA, PPE-A with that of PtOEP.

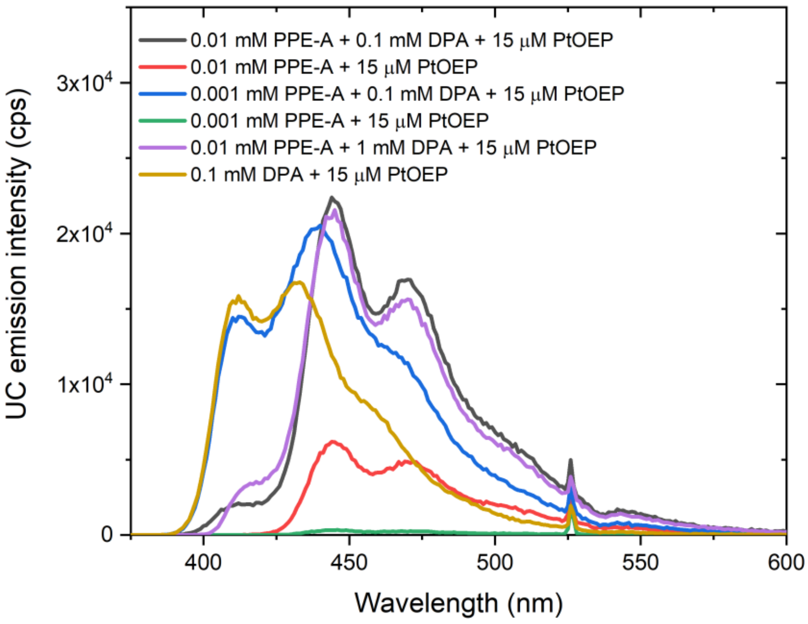

Figure S42: TTA-UC emission of system with PtOEP sensitizer with PPE-A annihilator in the presence or absence of mediator DPA Same excitation intensity used in all the case. All measurements are made at high excitation intensity of 35 W/cm^2

12.NMR data

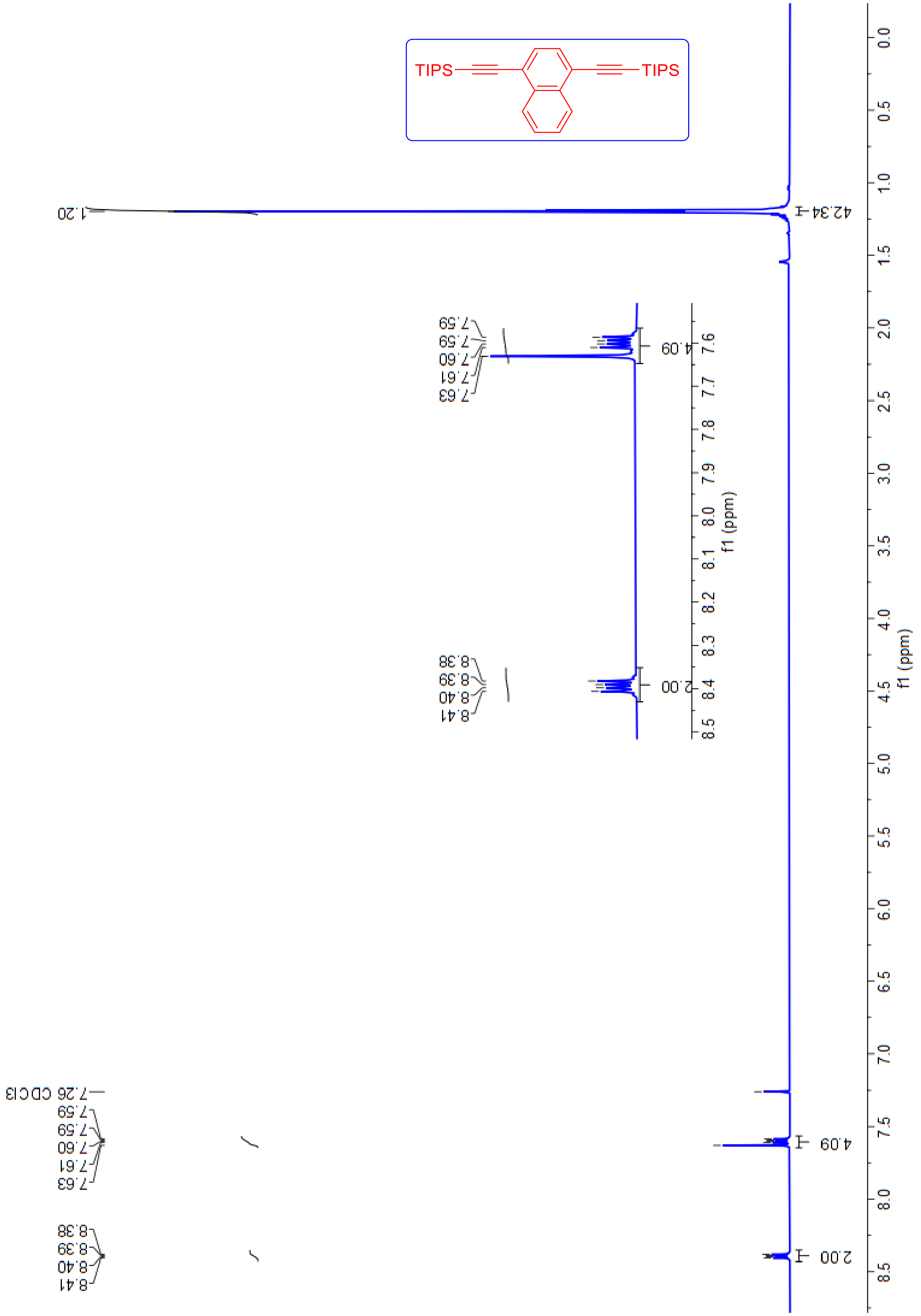

Figure S43: ^{1}H-NMR spectra of Nap.

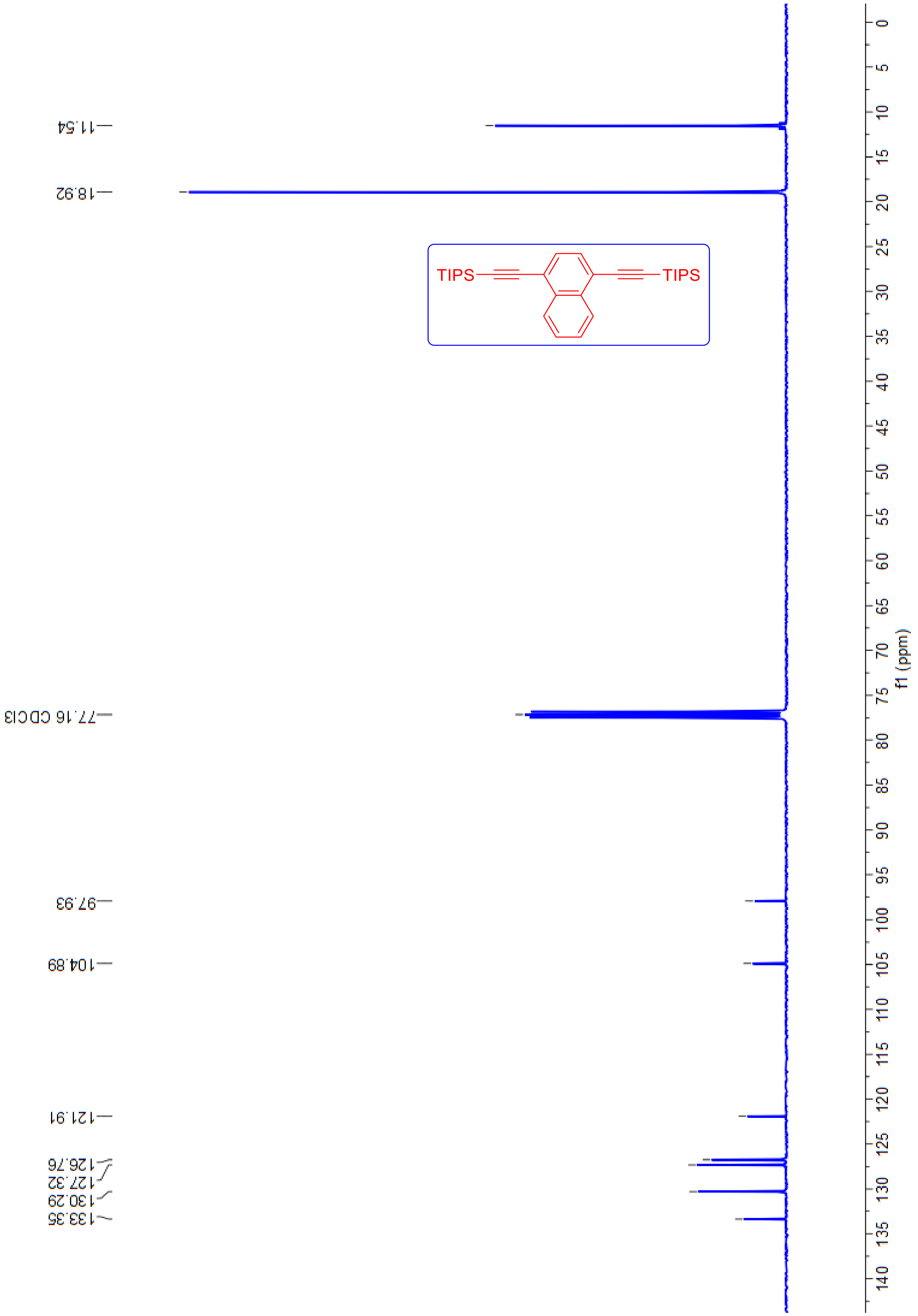

Figure S44: ^{13}C-NMR spectra of Nap.

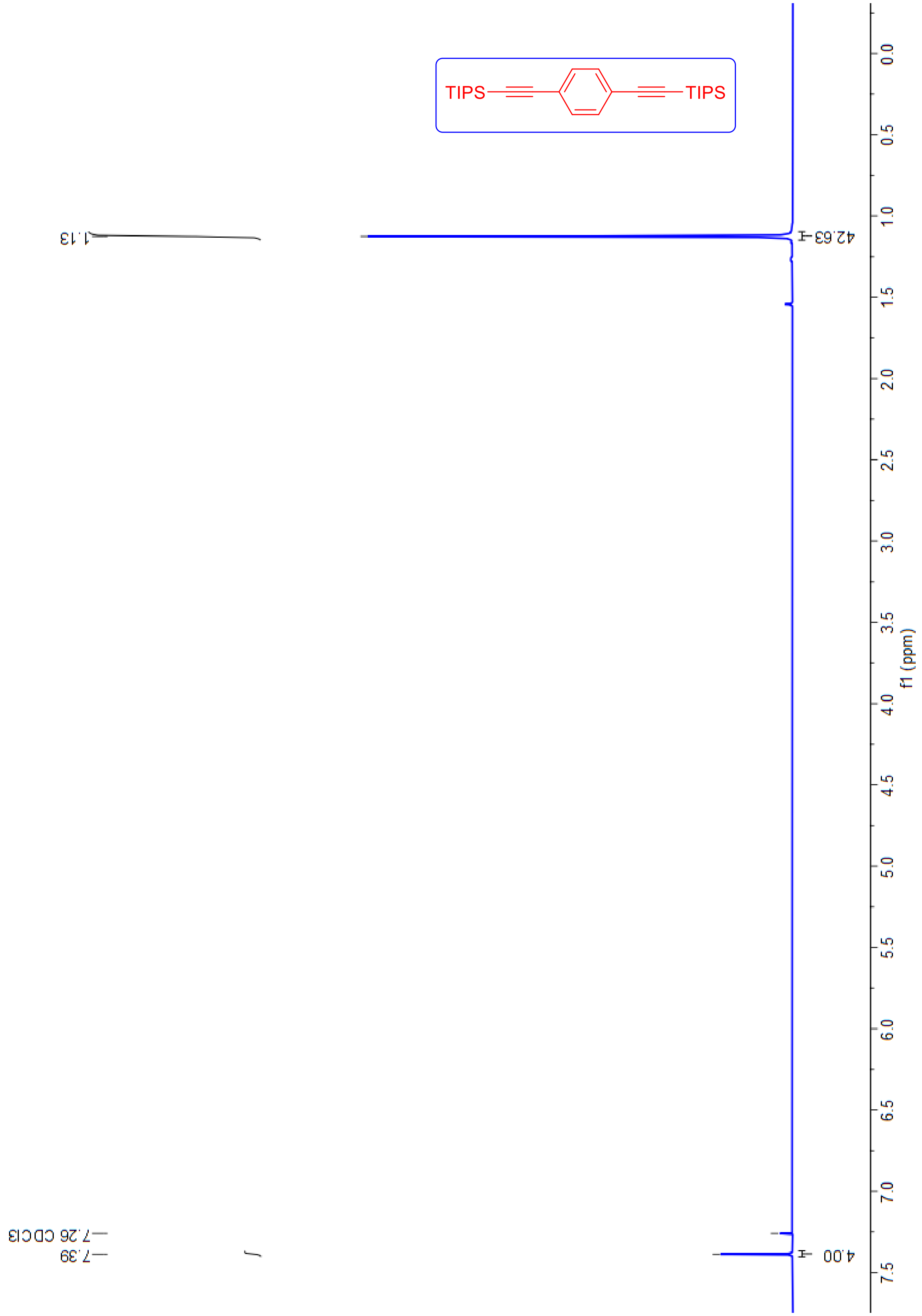

Figure S45: ^{1}H-NMR spectra of Ph.

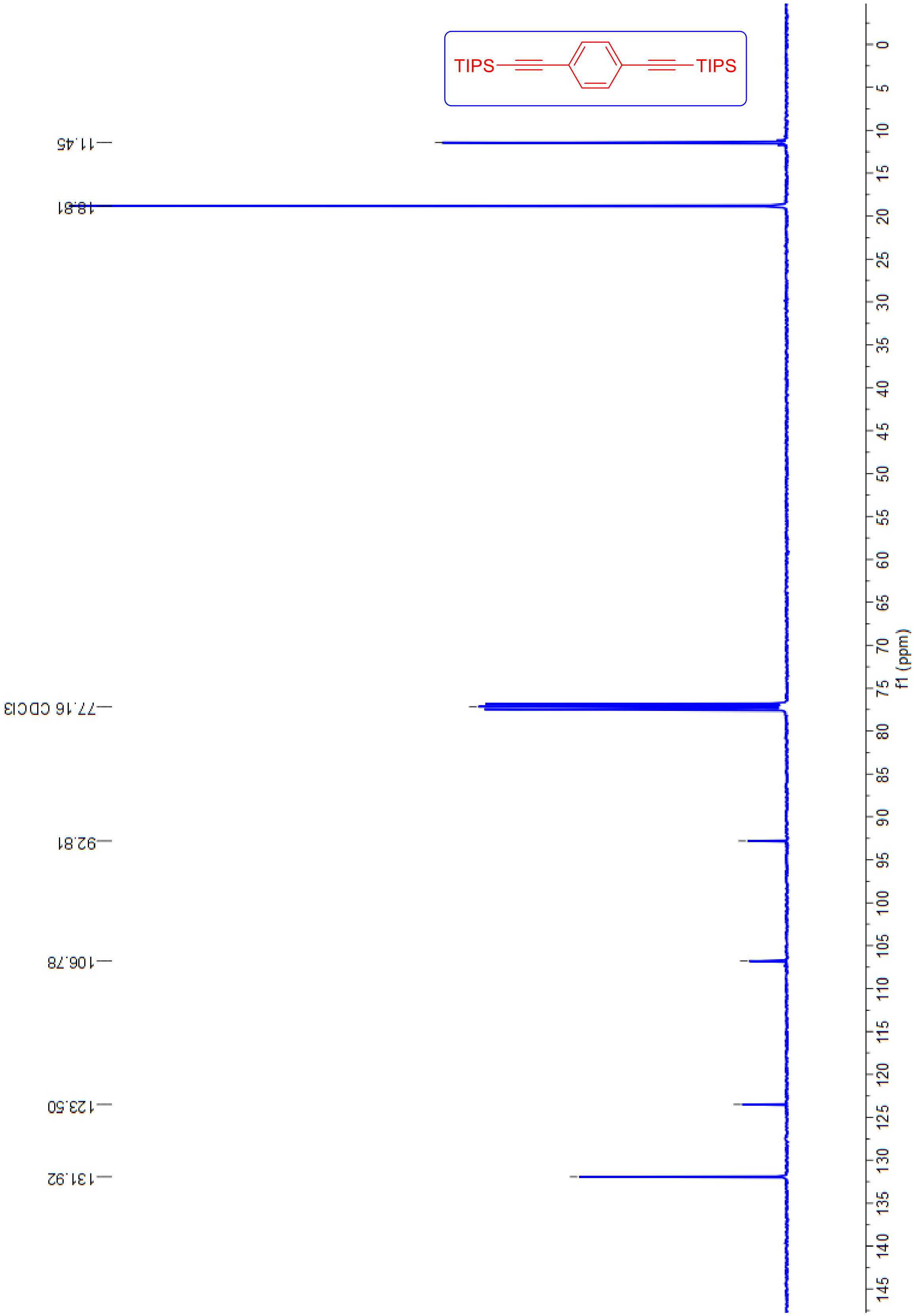

Figure S46: ^{13}C-NMR spectra of Ph.

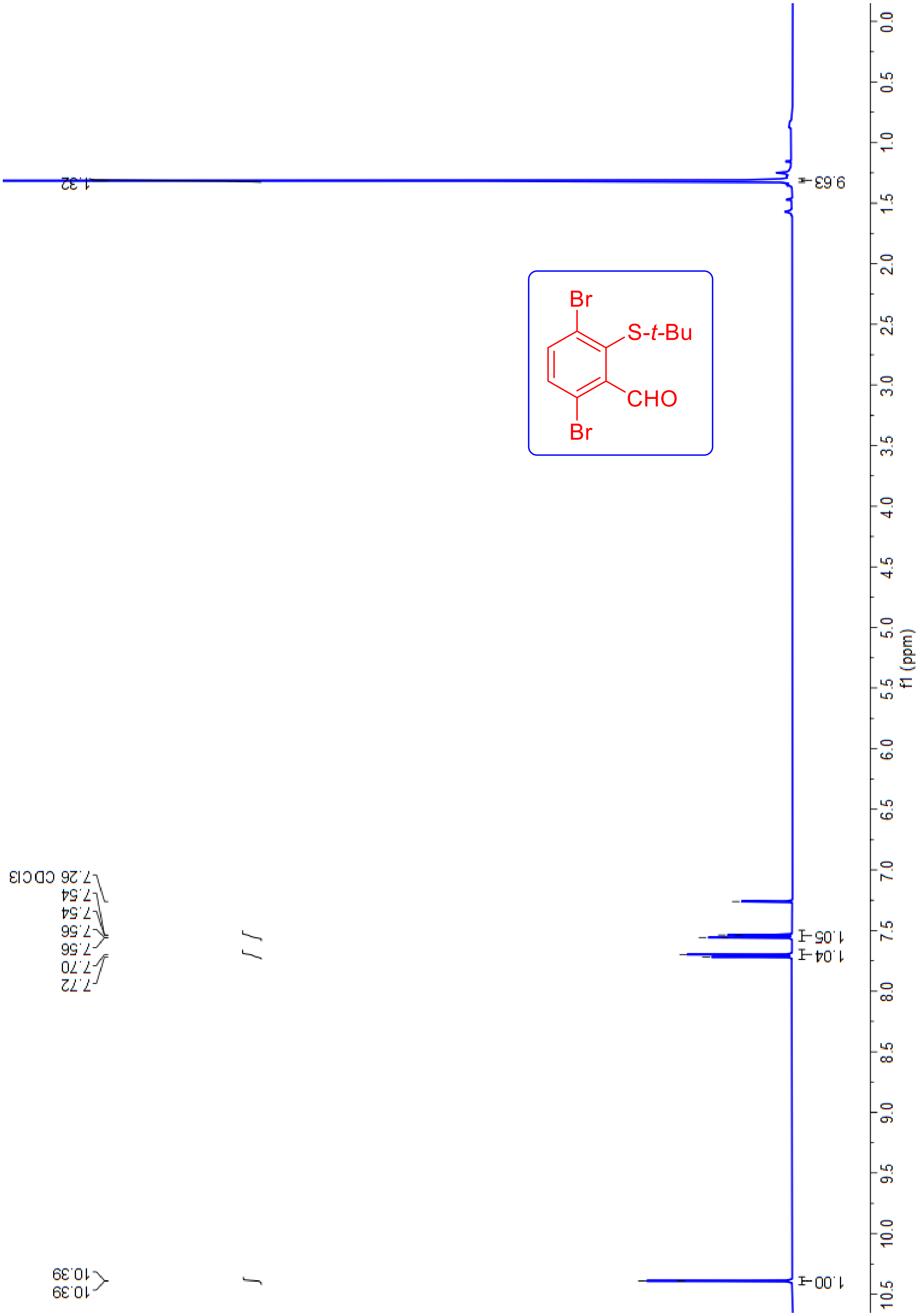

Figure S47: ^{1}H-NMR spectra of compound **3**.

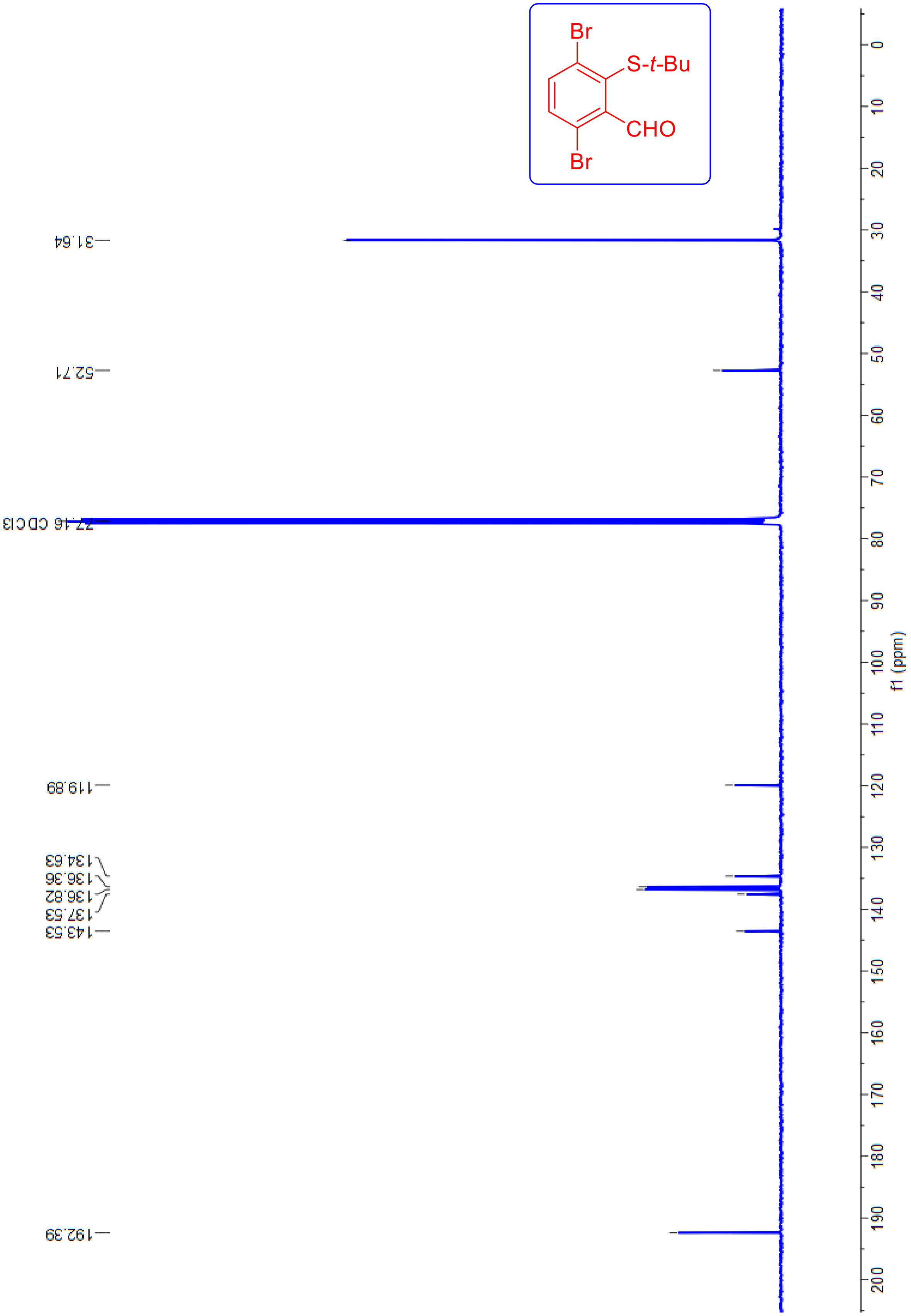

Figure S48: ^{13}C-NMR spectra of compound **3**.

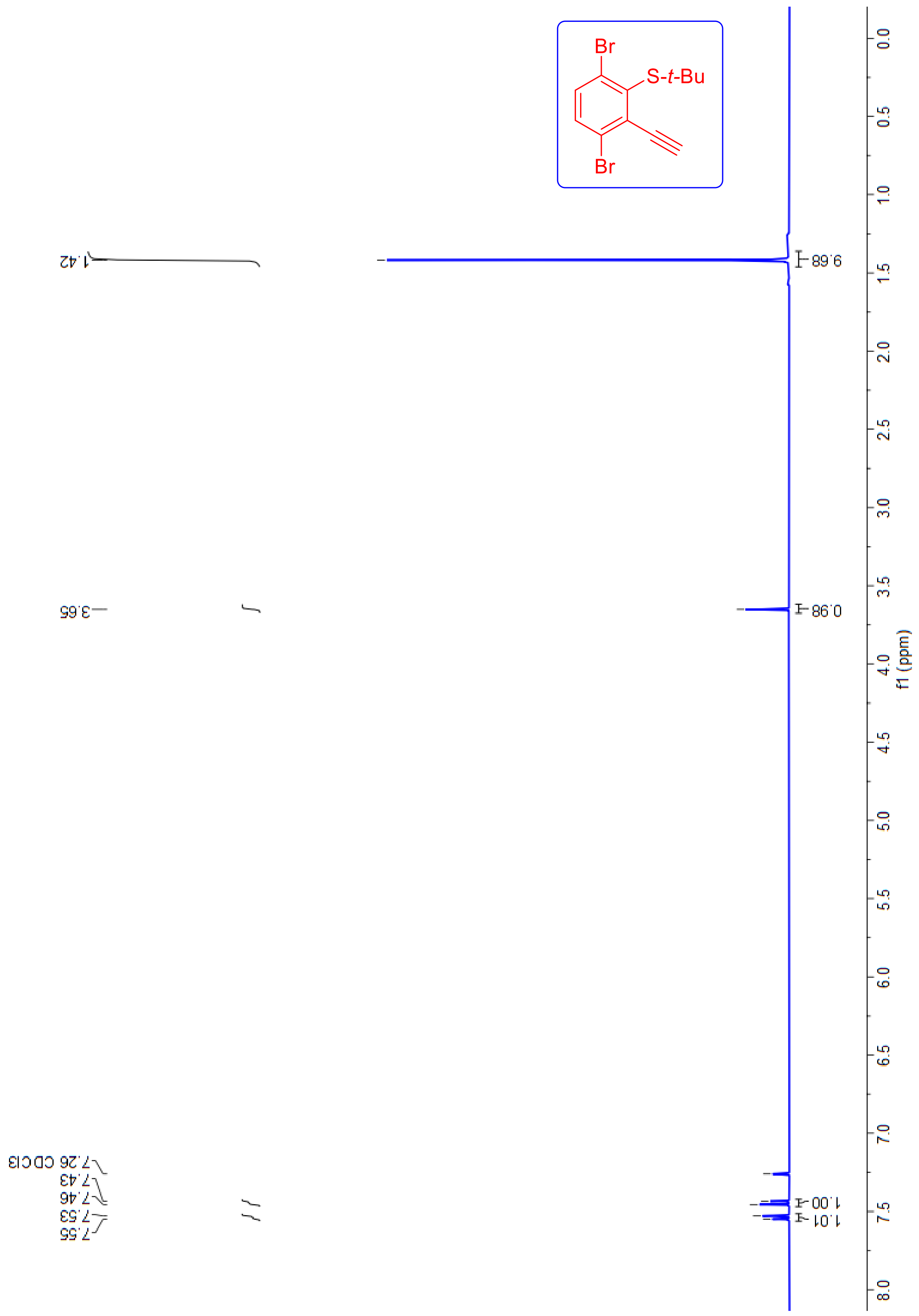

Figure S49: ^{1}H-NMR spectra of compound **4**.

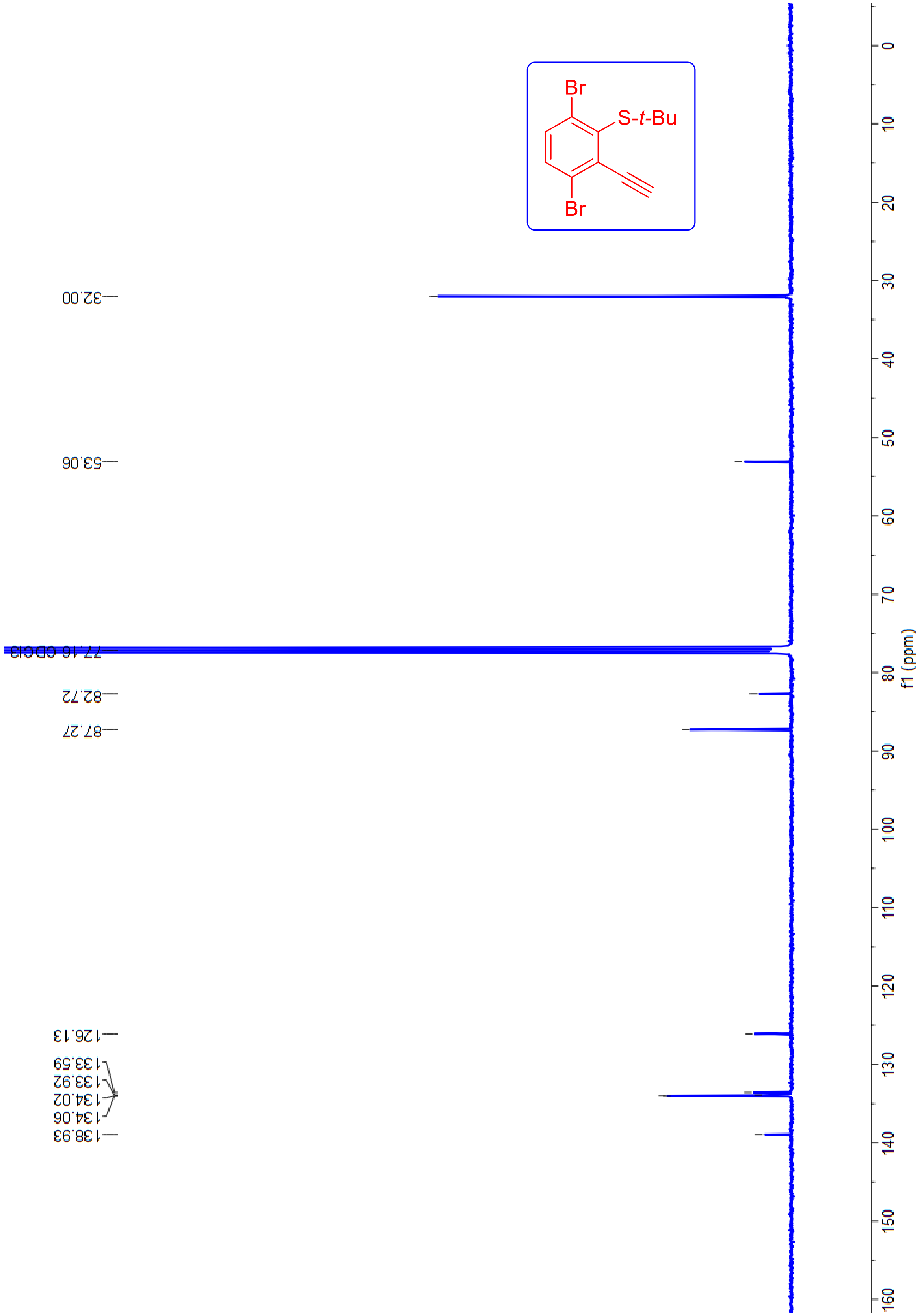

Figure S50: ^{13}C-NMR spectra of compound **4**.

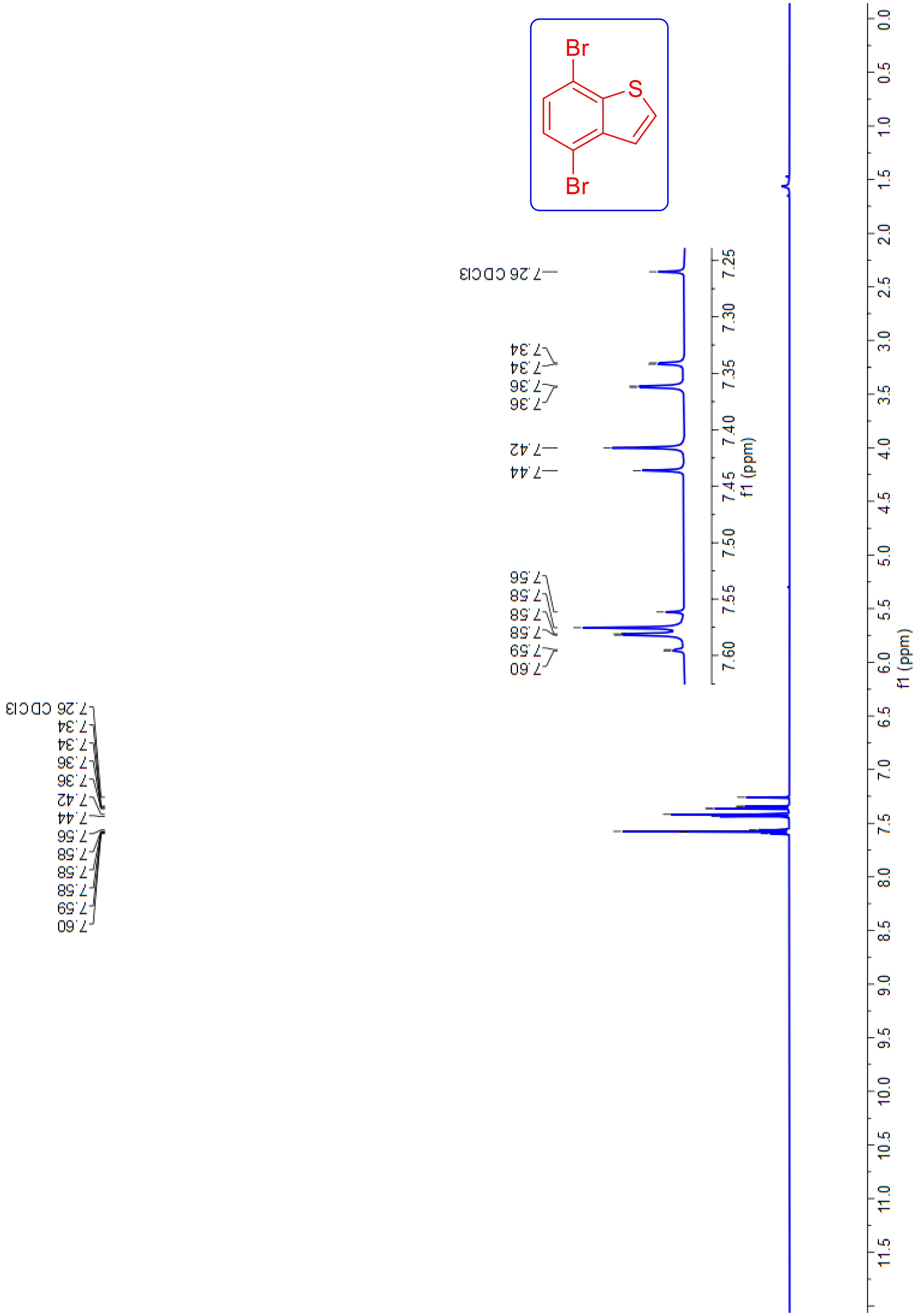

Figure S51: ^{1}H-NMR spectra of compound **5**.

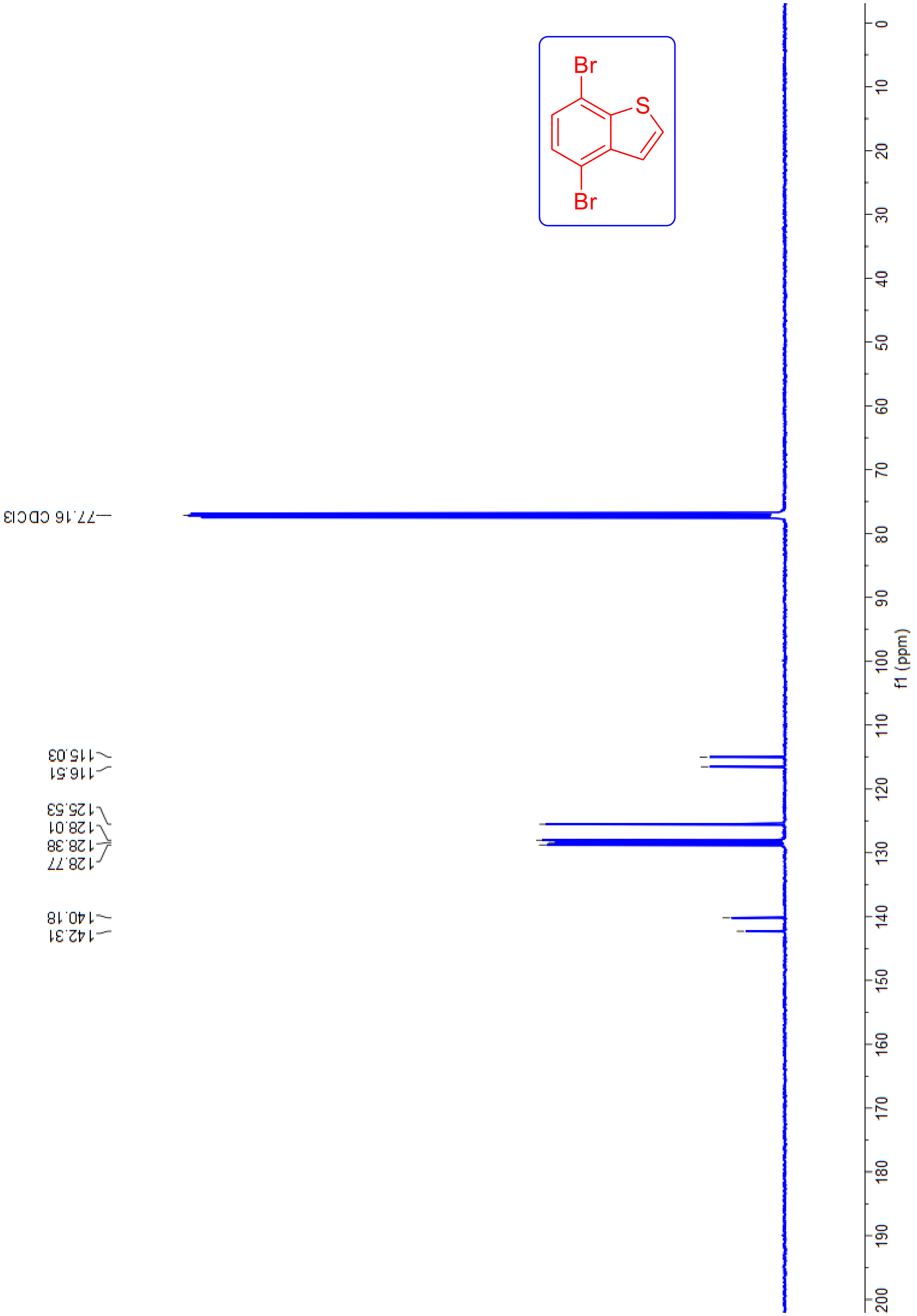

Figure S52: ^{13}C-NMR spectra of compound **5**.

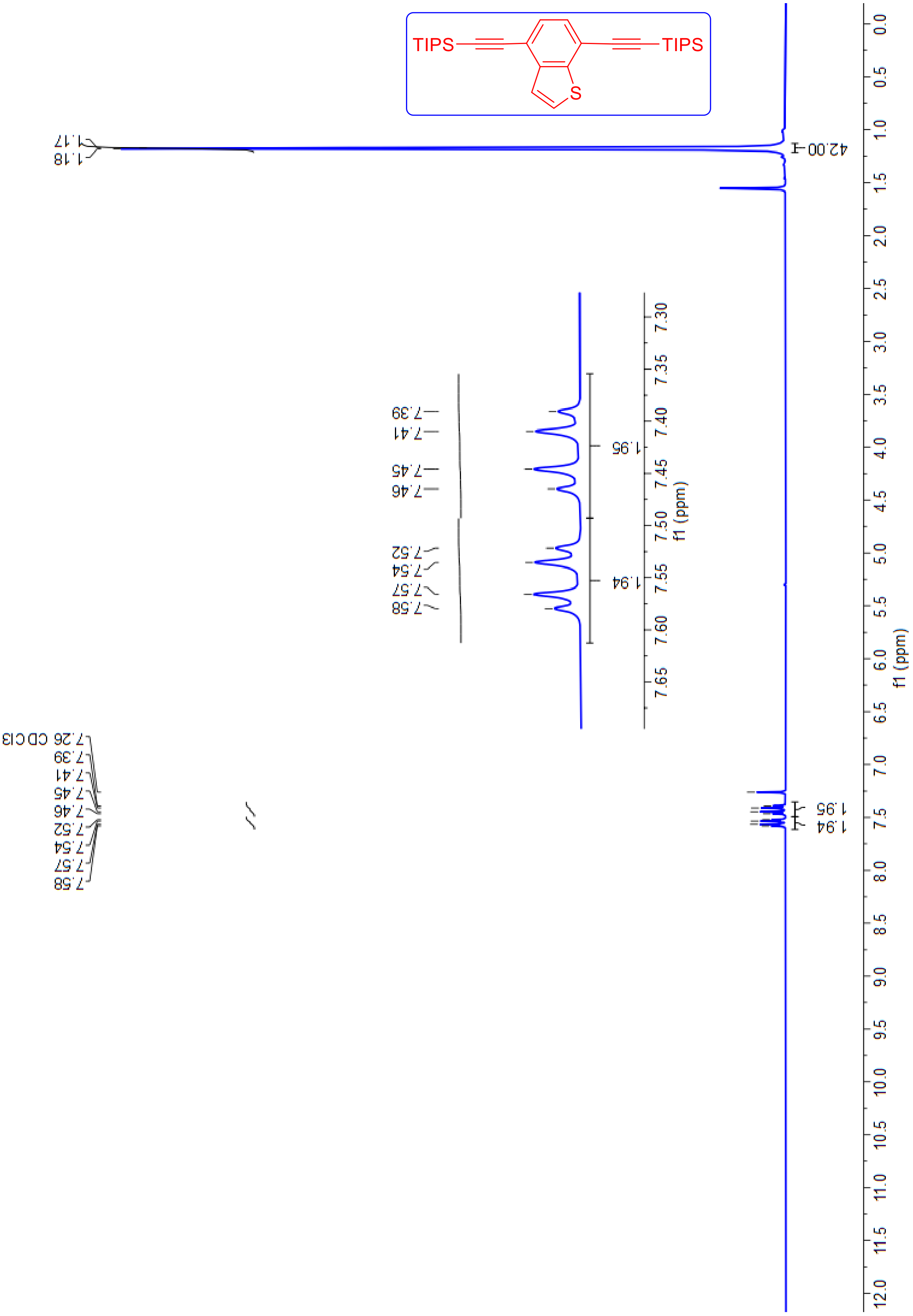

Figure S53: ^{1}H-NMR spectra of BT.

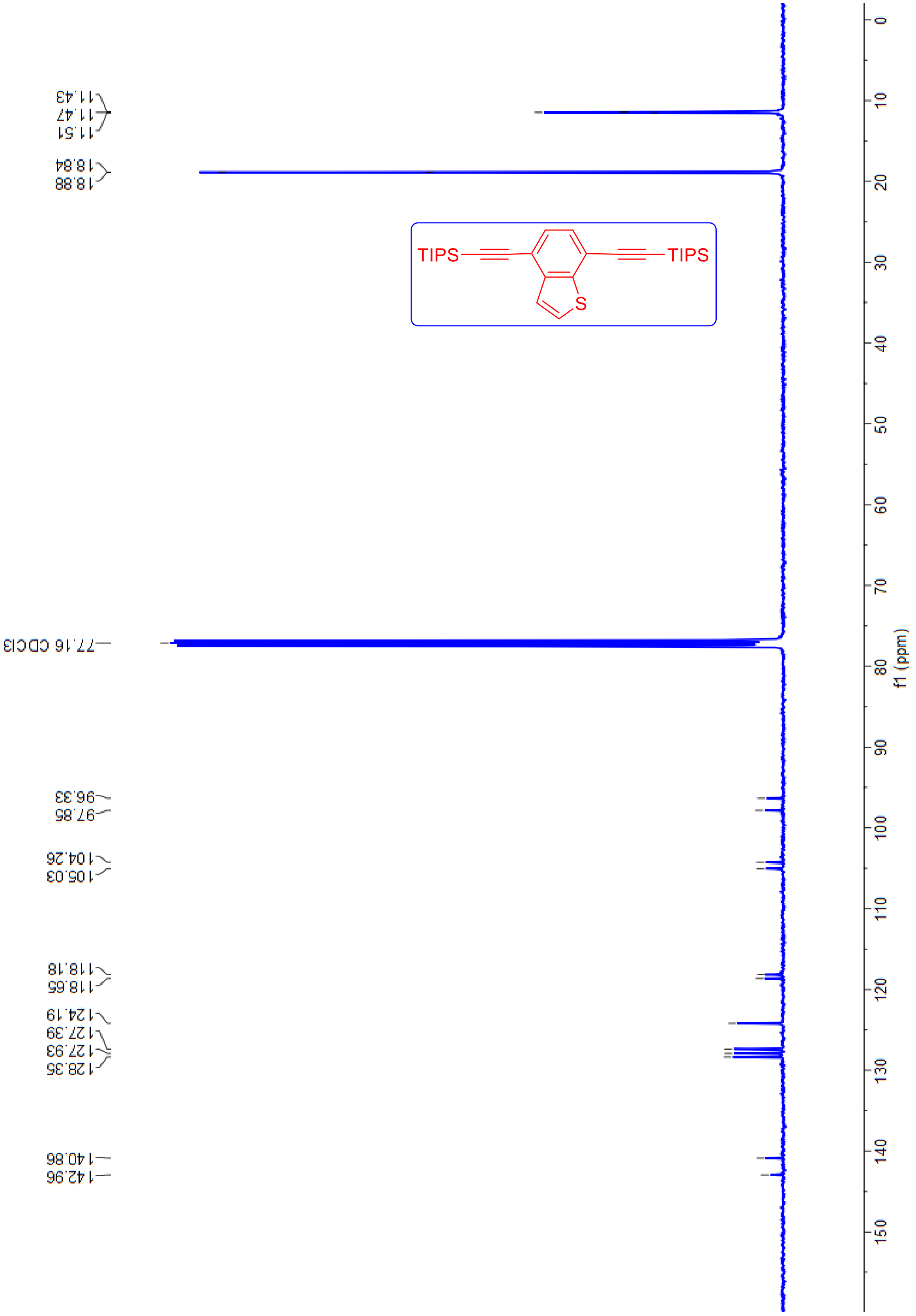

Figure S54: ^{13}C-NMR spectra of BT

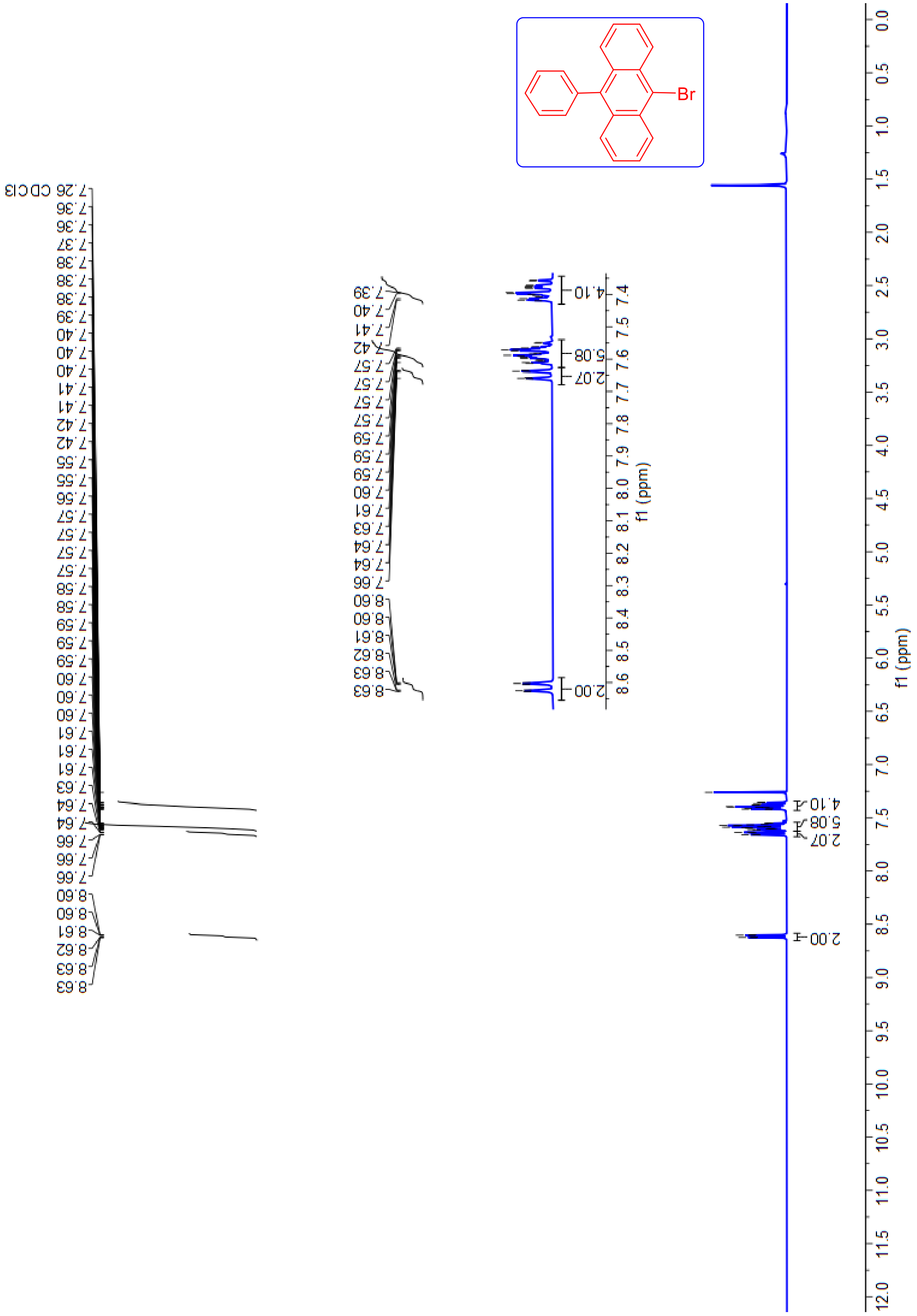

Figure S55: ^{1}H-NMR spectra of compound **19**.

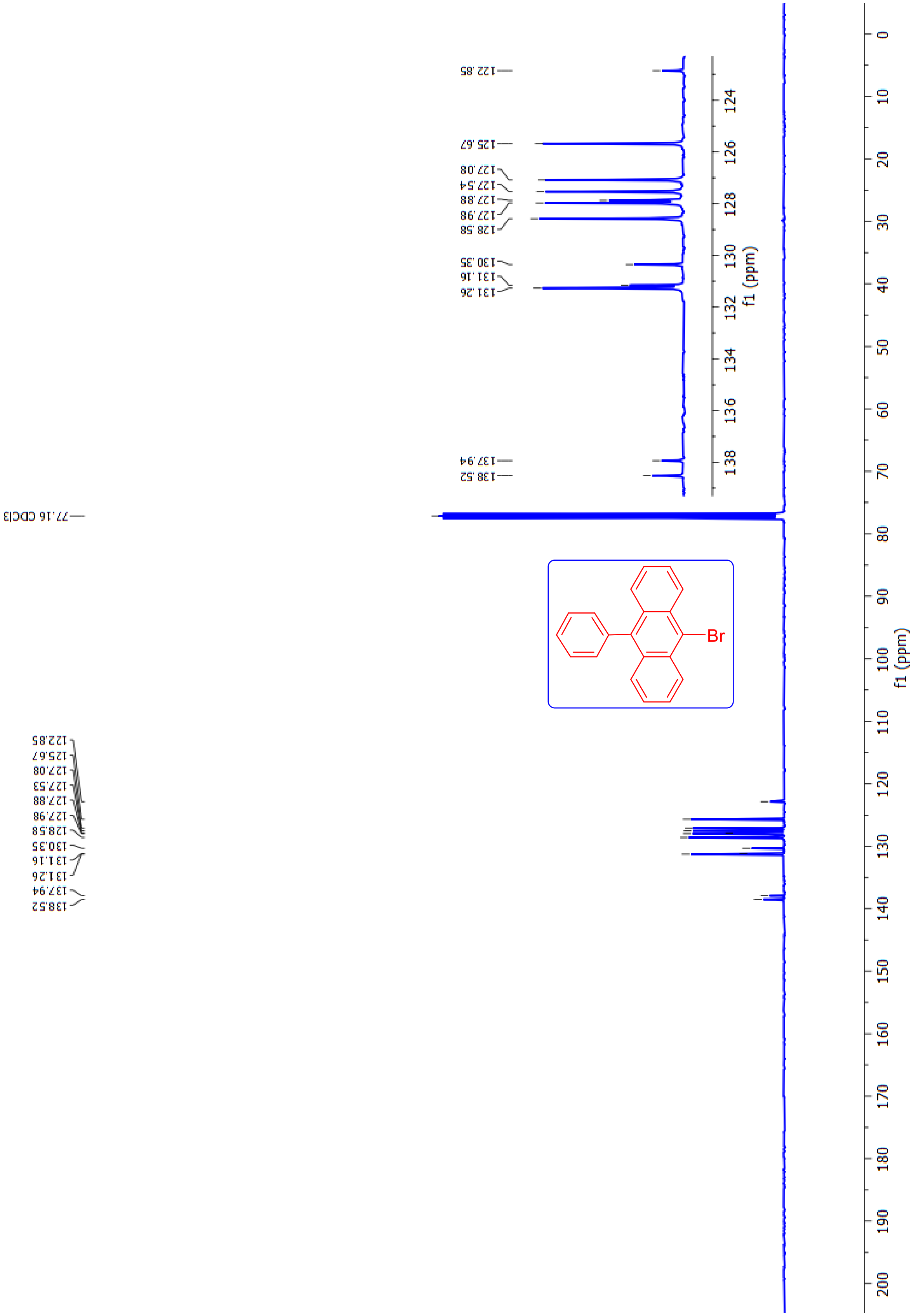

Figure S56: ^{13}C-NMR spectra of compound **19**.

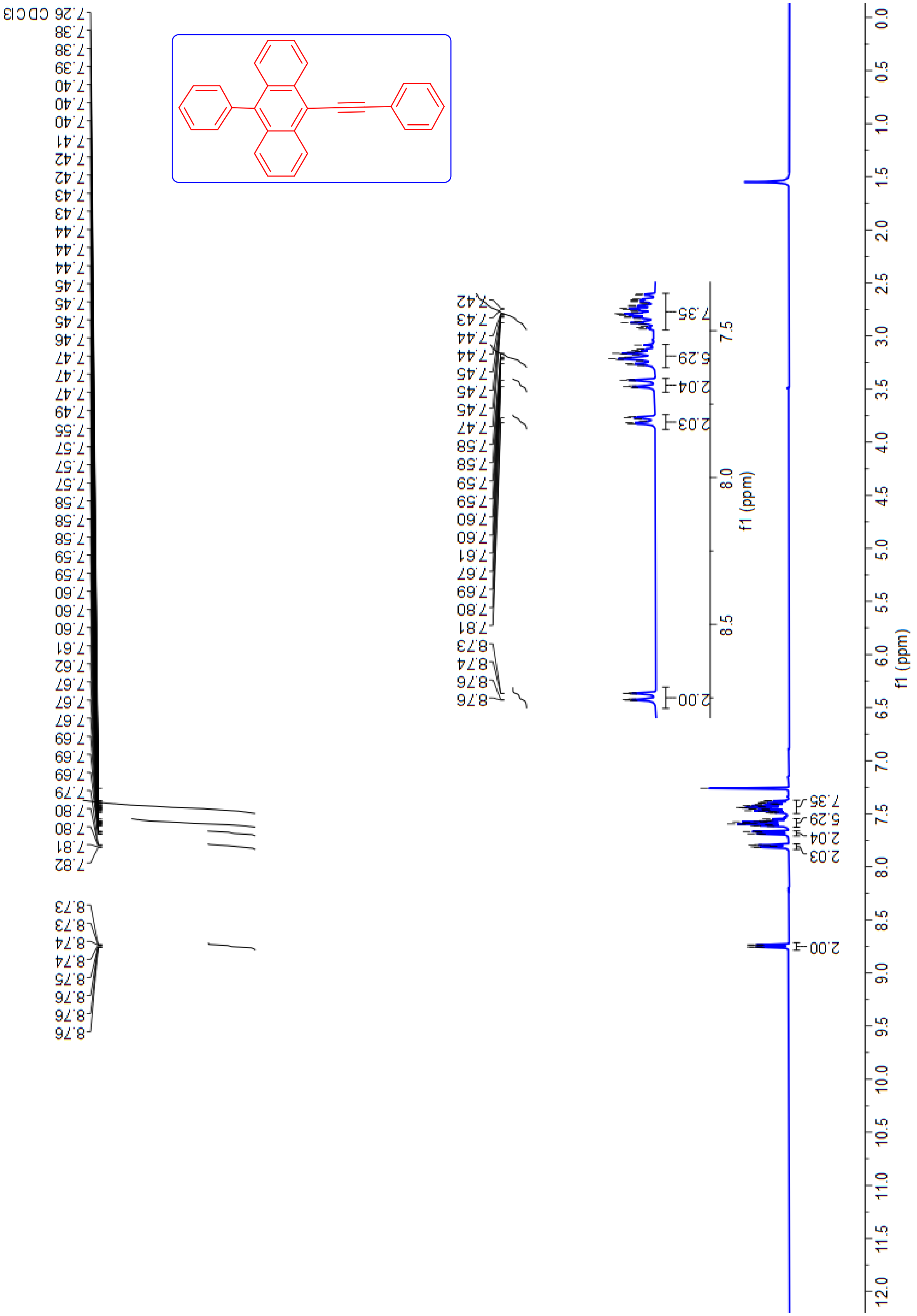

Figure S57: ^{1}H-NMR spectra of PPE-A.

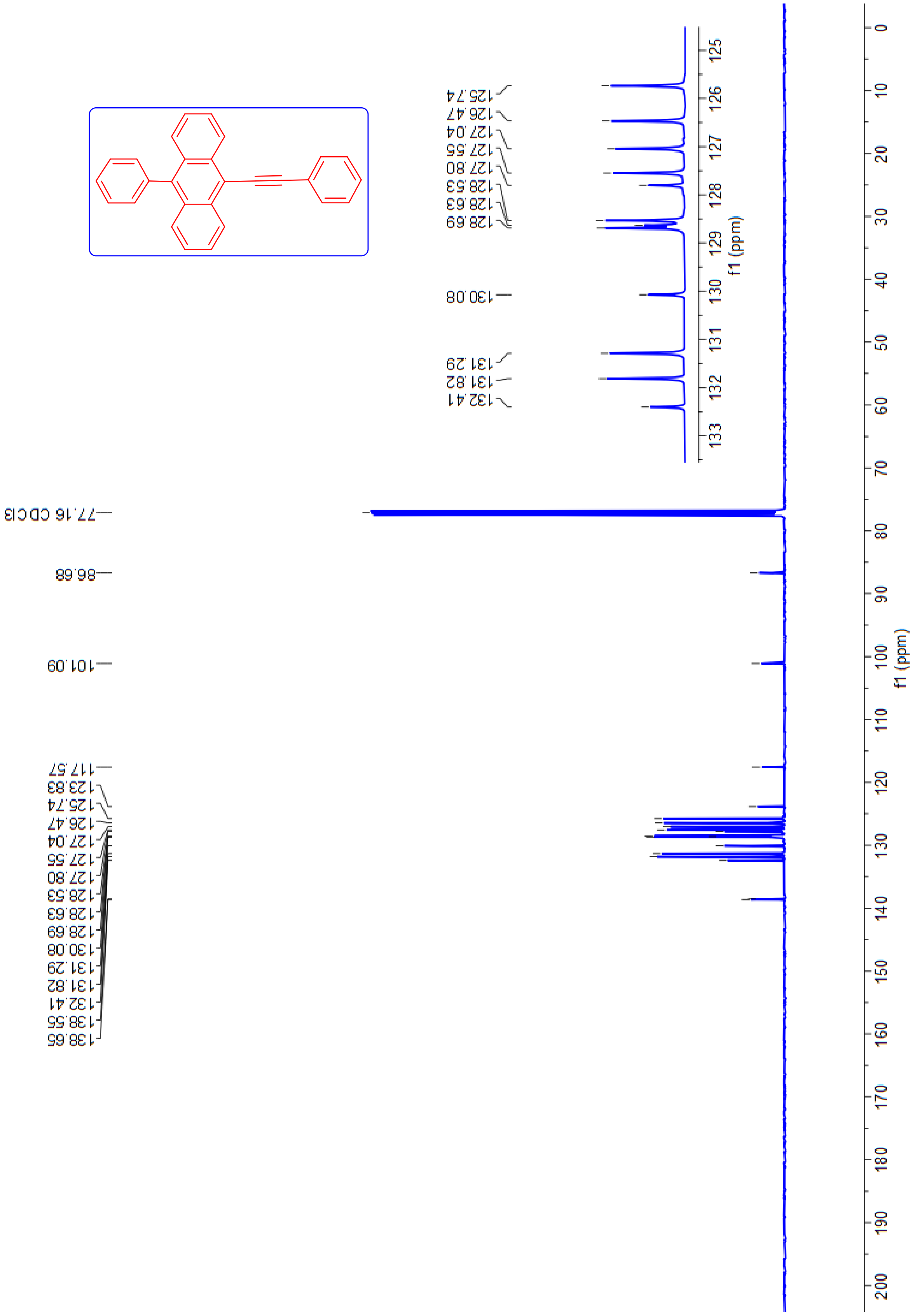

Figure S58: ^{13}C-NMR spectra of PPE-A

13. Kinetic Modelling

Kinetic modelling was done using MATLAB. The code is available to download at the Zenodo repository (http://zenodo.org) with DOI: 10.5281/zenodo.15266985. The differential equations describing the trimolecular system used for the modelling are described below:

$$\frac{d[^3Sen^*]}{dt} = k_{Ex}[Sen] - k_S[^3Sen^*] - k_{TET}[^3Sen^*][M] - k_{TET}[^3Sen^*][A] \quad (1)$$

$$\frac{d[^3M^*]}{dt} = k_{TET}[^3Sen^*][M] - 2k_{TTA}^{homo}[^3M^*]^2 - k_T^M[^3M^*] - k_{TET}^{M\to A}[^3M^*][A] - k_{TTA}^{hetero}[^3M^*][^3A^*] + k_{bTET}^{A\to M}[^3A^*][M] \quad (2)$$

$$\frac{d[^1M^*]}{dt} = k_{TTA}^{homo}[^3M^*]^2 - k_{FL}^M[^1M^*] \quad (3)$$

$$\frac{d[^3A]}{dt} = k_{TET}[^3Sen^*][A] + k_{TET}^{M\to A}[^3M^*][A] - 2k_{TTA}^{homo}[^3A^*]^2 - k_T^A[^3A^*] - k_{TTA}^{hetero}[^3M^*][^3A^*] - k_{bTET}^{A\to M}[^3A^*][M] \quad (4)$$

$$\frac{d[^1A^*]}{dt} = k_{TTA}^{homo}[^3A^*]^2 + k_{TTA}^{hetero}[^3M^*][^3A^*] - k_{FL}^A[^1A^*] \quad (5)$$

$$\frac{d[M]}{dt} = k_{FL}^M[^1M^*] - k_{TET}[^3Sen^*][M] + k_{TET}^{M\to A}[^3M^*][A] + k_{TTA}^{homo}[^3M^*]^2 + k_T^M[^3M^*] - k_{bTET}^{A\to M}[^3A^*][M] + k_{TTA}^{hetero}[^3M^*][^3A^*] \quad (6)$$

$$\frac{d[A]}{dt} = k_{FL}^A[^1A^*] - k_{TET}[^3Sen^*][A] - k_{TET}^{M\to A}[^3M^*][A] + k_{TTA}^{homo}[^3A^*]^2 + k_T^A[^3A^*] + k_{bTET}^{A\to M}[^3A^*][M] \quad (7)$$

$$\frac{d[Sen]}{dt} = -k_{Ex}[Sen] + k_S[^3Sen^*] + k_{TET}[^3Sen^*][M] + k_{TET}[^3Sen^*][A] \quad (8)$$

Where Sen refers to sensitizer, M to mediator, A to annihilator and [..] denotes concentration. k_{Ex} is the rate constant for excitation (s^{-1}) obtained from the excitation photon flux (photon s^{-1} cm^{-2}) and absorption cross-section (α, cm^2), $k_{Ex} = \alpha \times flux$. k_S is the rate constant for sensitizer triplet decay (s^{-1}), k_{TET} and $k_{TET}^{M\to A}$ are the bimolecular rate constants (s^{-1} M^{-1}) for triplet energy transfer from sensitizer and from mediator to annihilator, respectively. $k_{bTET}^{A\to M}$ is the bimolecular rate constant for back triplet energy transfer from annihilator to mediator estimated from the energy difference ($\Delta E = 0.26$ eV) between the mediator and annihilator triplet energies ($k_{bTET}^{A\to M} = k_{TET}^{M\to A} e^{\frac{-\Delta E}{k_bT}}$). k_{TTA}^{homo} and k_{TTA}^{hetero} are the bimolecular rate constants for homo and hetero TTA, respectively. k_{FL}^i is the rate constant of singlet decay (s^{-1}), and k_T^i the rate constant of triplet decay (s^{-1}), of compound i.

The rate constants, their values and how they were determined is summarized in Table S5 below.

Table S5: Values for rate constants used in the modelling and how they are determined.

Rate constant	Meaning	From	Value

k_{Ex}	Rate of excitation	Fixed to 0 for time resolved fits and to $k_{Ex} = \alpha \times flux$ for steady-state solutions	0 – 54568 s^{-1}
α	Absorption cross-section of sensitizer	Fixed, derived from molar absorptivity (7000 $M^{-1}cm^{-1}$)	2.6765 $\times10^{-17}$ cm^2
$flux$	Laser power in photons/s/cm^2	Fixed, measured with power meter.	0 – 2.0 $\times10^{21}$ photons/s/cm^2
k_S	Rate of triplet decay of sensitizer	Fixed, from TADF decay using TCSPC, Fig S31	1.6667 $\times10^{5}$
k_{TET}	Bimolecular triplet energy transfer rate constant from sensitizer to mediator/annihilator	Fixed, from Stern-Volmer quenching experiments Fig S30 and Fig S31	0.8 $\times10^{9}$ M^{-1} s^{-1}
k_{TTA}^{homo}	bimolecular rate constants for homo TTA	Fitting parameter in 2-component systems (Fig 4d & 4e). Fixed in 3-component model	1 $\times10^{9}$ M^{-1} s^{-1}
$k_{TET}^{M\to A}$	Bimolecular triplet energy transfer rate constant from mediator to annihilator	Manually varied starting with a reasonable diffusion limited value of 1$\times10^{9}$ $M^{-1}s^{-1}$	2 $\times10^{9}$ M^{-1} s^{-1}
k_{TTA}^{hetero}	bimolecular rate constants for hetero TTA	Manually varied as $y * k_{TTA}^{homo}$ to match UC emission kinetics in Figure 5a and S66	$2 * k_{TTA}^{homo}$
$k_{bTET}^{A\to M}$	Bimolecular triplet energy transfer rate constant from annihilator back to mediator	Fixed based on energy difference and forward rate constant: $k_{bTET}^{A\to M} = k_{TET}^{M\to A} e^{\frac{-\Delta E}{k_b T}}$	8 $\times10^{4}$ M^{-1} s^{-1}
k_{FL}^{A}	Rate constant of singlet decay (s^{-1}) of annihilator (both radiative and non-radiative)	Fixed, to the inverse of the fluorescence life-time determined with TCSPC Fig S35	5.13 $\times10^{8}$ s^{-1}
k_{FL}^{M}	Rate constant of singlet decay (s^{-1}) of mediator (both radiative and non-radiative)	Fixed, to the inverse of the fluorescence life-time determined with TCSPC Fig S36	5.26 $\times10^{8}$ s^{-1}
k_{T}^{A}	Rate constant of triplet decay (s^{-1}) of annihilator	Fixed during fit of k_{TTA}^{homo}, then recalculated as $k_T^A = \sqrt{I_{th}([Sens]2\alpha k_{TTA}^{homo})}$ k_T^A is then recalculated after the fit, and the fit re-run with the new value until k_T^A	5.15 $\times10^{3}$ s^{-1}

		converges. I_{th} determined in Fig S3, Table S2	
k_T^M	Rate constant of triplet decay (s^{-1}) of annihilator	As for k_T^A but I_{th} determined from Fig S6, Table S2	$6.4 \times 10^3\ s^{-1}$

We use the built in MATLAB function ode23s to solve the set of differential equations with kEx = 0 and an initial triplet sensitizer concentration estimated from the nsTA ground state bleach, to extract the population dynamics over time. The time range is divided into smaller ranges to ensure smoot solutions using ode23s. The same set of equations can be solved for the bimolecular systems by setting either the mediator or annihilator ground state concentration to 0 M. A least-square fitting routine using the MATLAB function fminsearch is employed to fit the bimolecular model to the corresponding nsTA traces of the annihilator and mediator triplet signals, respectively. The rate constant for homo TTA (k_{TTA}^{homo}) is used as fitting parameter and the triplet lifetime is calculated continuously from the experimentally determined threshold intensity and k_{TTA}^{homo} as described in the main text Equation 2. k_{TET}, k_S, and k_{FL}^{i} are determined from complimentary experiments, see Table S5. No other rate constants are required for the bimolecular fits. For the trimolecular models the same rate constants are used as obtained from the bimolecular fits, and values for $k_{TET}^{M\to A}$and k_{TTA}^{hetero} are roughly estimated manually to ensure the model best reproduces the experimental nsTA data.

For steady-state solutions we use the same set of equations and set them equal to 0, with the same rate constants as discussed above we use the built in MATLAB function fsolve to determine the steady-state concentrations of each species. The steady-state upconversion quantum yields of the annihilator (Φ_{UC}^{A}) and mediator (Φ_{UC}^{M}) are then calculated from the steady-state concentrations as per the equations below:

$$\Phi_{UC}^{A} = \frac{0.42 k_r [^1A^*]}{k_{Ex}[Sen]} \tag{9}$$

$$\Phi_{UC}^{M} = \frac{0.37 k_r [^1M^*]}{k_{Ex}[Sen]} \tag{10}$$

The prefactors 0.42 and 0.37 are the spin-factors determined for the bimolecular systems obtained by matching the modelled quantum yield to the experimentally obtained quantum yield.

13.1. Figures from kinetic modelling

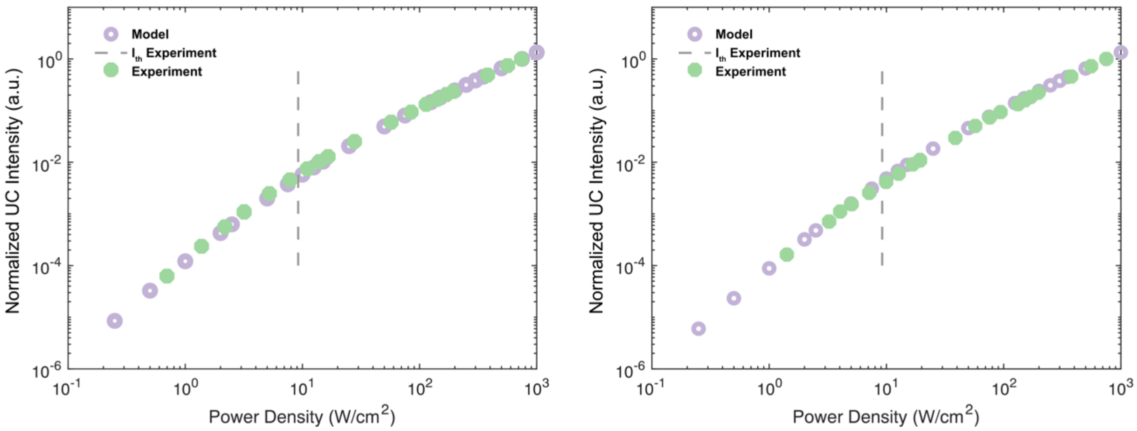

Figure S59: Experimental and modelled UC intensity dependence with 25 μM 4CzBN and 1mM Nap (left) or 1mMBT (right).

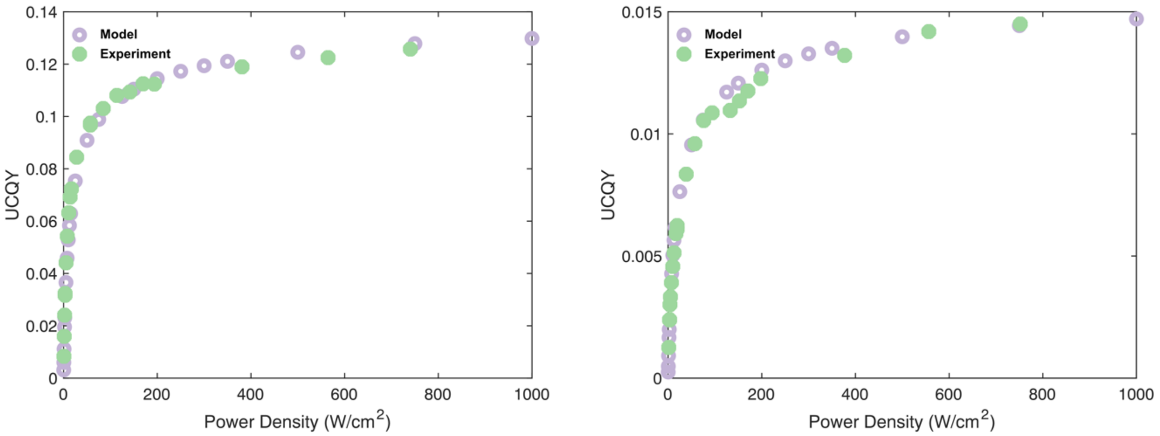

Figure S60: Experimental and modelled TTA-UC QYs with 25 μM 4CzBN and 1mM Nap (left) or 1mMBT (right) at different excitation power densities.

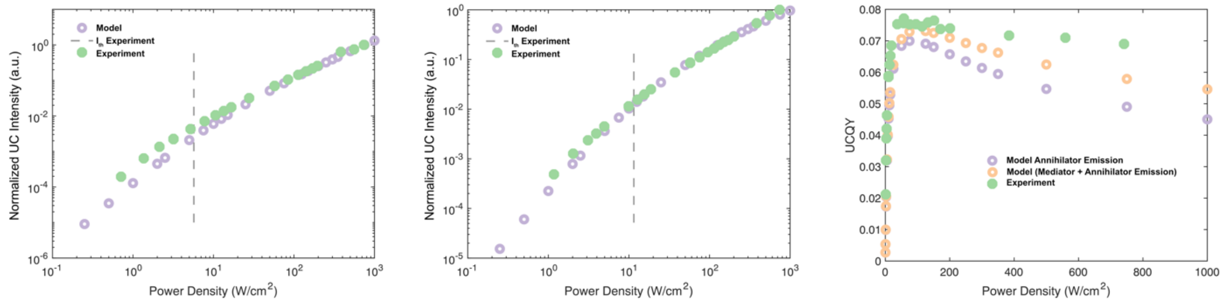

Figure S61: Experimental and modelled TTA-UC intensity dependence (left and center) and QYs (right) for 25 μM 4CzBN, 1mM mediator BT and 0.1 mM (left) or 0.01 mM (center and right) Nap at different excitation power densities

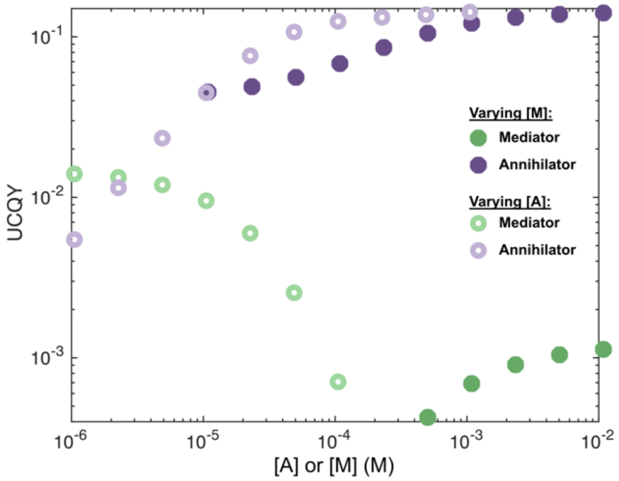

Figure S62: Modelled effect on the TTA-UC QY for annihilator and mediator emission, respectively, as a function of annihilator (open) and mediator (filled) concentration. For variations in annihilator concentration the mediator concentration is kept at 1 mM and for variations in mediator concentration the annihilator is kept at 0.1 mM. All rate constants are used as determined in the main manuscript and in the models above.

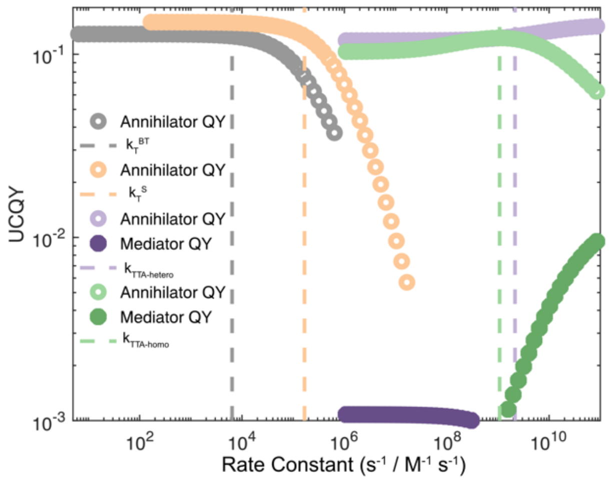

Figure S63: Modelled effect of the TTA-UC quantum yield for annihilator (open) and mediator (closed) emission, respectively, as a function of different rate constants. Dashed lines show the experimentally determined value for the rate constant in question for the current 25 μM 4CzBn/1mM BT/0.1mM Nap system.

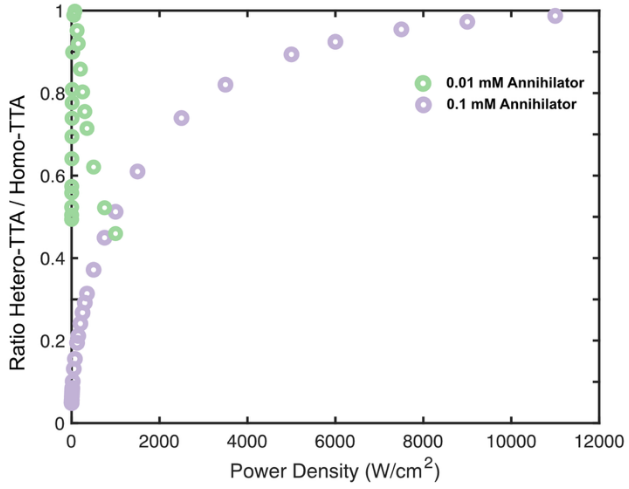

Figure S64: The ratio of hetero-TTA and the sum of annihilator and mediator homo-TTA as a function of excitation power density for low (0.01 mM, green) and high (0.1 mM, purple) concentrations of annihilator.

13.2. Regarding Mediator to Annihilator Singlet Energy Transfer

To assess the role of singlet energy transfer (SET) from mediator to annihilator, following mediator homo-TTA we performed front-face fluorescence measurement of 1 mM BT with 0.1 mM Nap, exciting at 310 nm, where BT absorbs ~94% of the absorbed photons, Figure S65. The observed fluorescence spectra can be reproduced by adding normalized BT and Nap emission spectra with 50/50 contribution. Taking into consideration the 7 times lower BT emission QY one would expect approximately 65/35 BT/Nap emission contribution. This could suggest up to 30% SET and/or reabsorption from BT to Nap. However, this is a rough estimate as it assumes that the BT QY in 1 mM solutions is the same as in dilute solutions, ignoring intrinsic BT reabsorption. Furthermore, SET cannot reproduce the UC kinetics adequately, as illustrated in Figure S66 as the modelled UC emission profile becomes too broad. Additionally, in the UC sample, BT S_1 states are not as efficiently formed, due to efficient TET from mediator to annihilator, and

even with a small proportion of SET taking place, it plays a minor role in the observed kinetics. For simplicity we thus exclude SET from the model for the discussion in the main text.

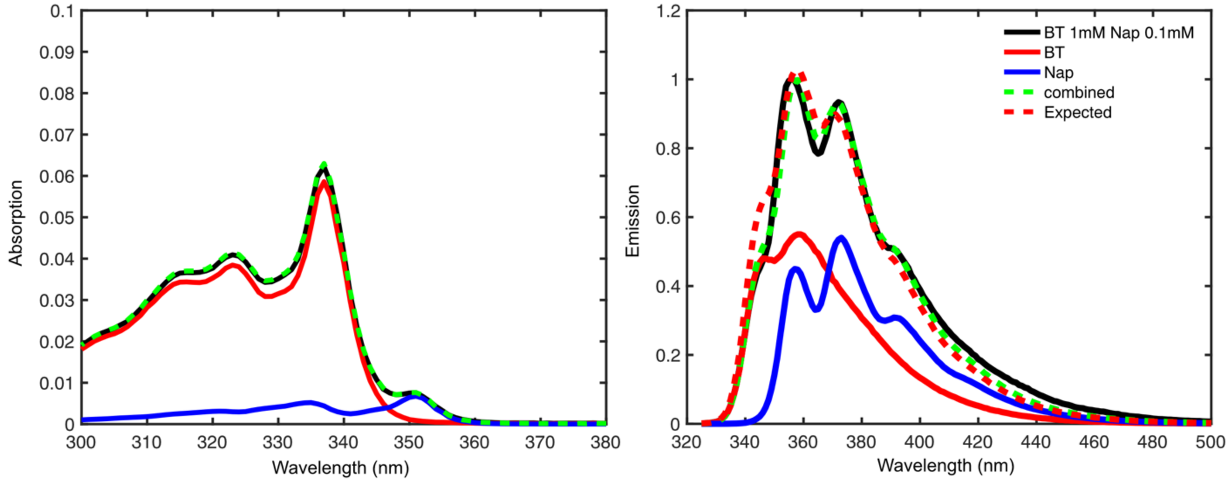

Figure 65. Left: Absorption spectra of samplse with: 1 mM BT and 0.1 mM Nap (black); 1 mM BT (red); 0.1 mM Nap (blue); and the fabricated spectra by adding the individual components together (green dashed). Right: Normalised emission spectra of 1 mM BT with0.1 mM Nap (black); 1 mM BT(red); 0.1 mM Nap (blue); and the combined emission spectra of adding 1:1 spectra of BT and Nap (green dashed); the expected spectra based on the ammount of absorption of BT and Nap at 310 nm, taking into account .the respective fluorescence quantum yields, and disregarding SET and reabsorption.

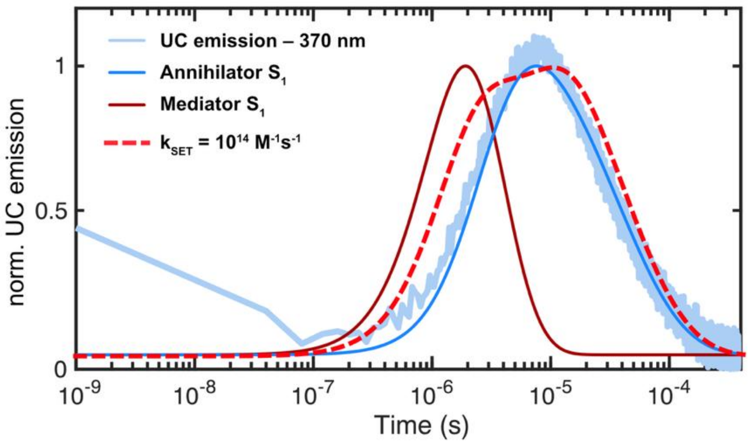

Figure S66. Kinetic trace of upconverted emission detected at 370 nm for a sample with 0.1 mM Nap, 1 mM BT, and 25 μM 4CzBN, excited at 410 nm with a pulse power of 2.3 mJ/pulse. Solid lines indicate normalized population dynamics of mediator and annihilator singlet states (S_1). Dashed line represents annihilator S_1 dynamics includingthe possibility for singlet energy transfer from mediator to annihilator wit ha rate constant of $1 \times 10^{14} M^{-1} s^{-1}$ and a hetero TTA rate (k_{TTA}^{hetero}) constant equal to the homo TTA rate constants (k_{TTA}^{homo}) for mediator and annihilator of $1 \times 10^{9} M^{-1} s^{-1}$.

14. References

1 G. R. Fulmer, A. J. M. Miller, N. H. Sherden, H. E. Gottlieb, A. Nudelman, B. M. Stoltz, J. E. Bercaw and K. I. Goldberg, *Organometallics*, 2010, **29**, 2176–2179.

2 R. J. Armstrong, W. Niwetmarin and V. K. Aggarwal, *Org. Lett.*, 2017, **19**, 2762–2765.

3 N. Harada, Y. Sasaki, M. Hosoyamada, N. Kimizuka and N. Yanai, *Angewandte Chemie International Edition*, 2021, **60**, 142–147.

4 T. Yamamoto, H. Katsuta, K. Toyota, T. Iwamoto and N. Morita, *Bulletin of the Chemical Society of Japan*, 2012, **85**, 613–623.

5 C. Würth, M. Grabolle, J. Pauli, M. Spieles and U. Resch-Genger, *Nature Protocols*, 2013, **8**, 1535–1550.
6 M. Levitus, *Methods and Applications in Fluorescence*, 2020, **8**, 033001.
7 A. Olesund, J. Johnsson, F. Edhborg, S. Ghasemi, K. Moth-Poulsen and B. Albinsson, *J. Am. Chem. Soc.*, 2022, **144**, 3706–3716.
8 D. Magde, R. Wong and P. G. Seybold, *Photochemistry and Photobiology*, 2002, **75**, 327–334.
9 A. Olesund, J. Johnsson, F. Edhborg, S. Ghasemi, K. Moth-Poulsen and B. Albinsson, *J. Am. Chem. Soc.*, 2022, **144**, 3706–3716.
10 Y. Zhou, F. N. Castellano, T. W. Schmidt and K. Hanson, *ACS Energy Lett.*, 2020, **5**, 2322–2326.
11 A. Olesund, V. Gray, J. Mårtensson and B. Albinsson, *J. Am. Chem. Soc.*, 2021, **143**, 5745–5754.
12 J. I. Day, K. N. Allen-Moyer and K. P. Cole, *J. Org. Chem.*, 2023, **88**, 4209–4223.
13 Z. Alassad, A. AboRaed, M. S. Mizrachi, M. H. Pérez-Temprano and A. Milo, *J. Am. Chem. Soc.*, 2022, **144**, 20672–20679.
14 S. Wu, L. A. Galán, M. Roux, F. Riobé, B. Le Guennic, Y. Guyot, T. Le Bahers, L. Micouin, O. Maury and E. Benedetti, *Inorg. Chem.*, 2021, **60**, 16194–16203.
15 Z. Huang, Z. Xu, T. Huang, V. Gray, K. Moth-Poulsen, T. Lian and M. L. Tang, *J. Am. Chem. Soc.*, 2020, **142**, 17581–17588.
16 V. Gray, A. Dreos, P. Erhart, B. Albinsson, K. Moth-Poulsen and M. Abrahamsson, *Phys. Chem. Chem. Phys.*, 2017, **19**, 10931–10939.
